# Fast and accurate sCMOS noise correction for fluorescence microscopy

**DOI:** 10.1038/s41467-019-13841-8

**Published:** 2020-01-03

**Authors:** Biagio Mandracchia, Xuanwen Hua, Changliang Guo, Jeonghwan Son, Tara Urner, Shu Jia

**Affiliations:** The Wallace H. Coulter Department of Biomedical Engineering, Georgia Institute of Technology and Emory University, Atlanta, GA USA

**Keywords:** Fluorescence imaging, Microscopy

## Abstract

The rapid development of scientific CMOS (sCMOS) technology has greatly advanced optical microscopy for biomedical research with superior sensitivity, resolution, field-of-view, and frame rates. However, for sCMOS sensors, the parallel charge-voltage conversion and different responsivity at each pixel induces extra readout and pattern noise compared to charge-coupled devices (CCD) and electron-multiplying CCD (EM-CCD) sensors. This can produce artifacts, deteriorate imaging capability, and hinder quantification of fluorescent signals, thereby compromising strategies to reduce photo-damage to live samples. Here, we propose a content-adaptive algorithm for the automatic correction of sCMOS-related noise (ACsN) for fluorescence microscopy. ACsN combines camera physics and layered sparse filtering to significantly reduce the most relevant noise sources in a sCMOS sensor while preserving the fine details of the signal. The method improves the camera performance, enabling fast, low-light and quantitative optical microscopy with video-rate denoising for a broad range of imaging conditions and modalities.

## Abstract (web summary)

Scientific complementary metal-oxide semiconductor (sCMOS) cameras have advanced the imaging field, but they often suffer from additional noise compared to CCD sensors. Here the authors present a content-adaptive algorithm for the automatic correction of sCMOS-related noise for fluorescence microscopy.

## Introduction

The accurate acquisition of diverse anatomical and dynamic traits within a cell that span spatiotemporal scales provides insights into the fundamentals of living organisms. In this context, scientific complementary metal-oxide semiconductor (sCMOS) cameras have rapidly been gaining popularity in optical microscopy for their higher frame rates, wider field-of-view, and lower electrical noise, compared to charge-coupled devices (CCD) or electron-multiplying CCDs (EM-CCD) cameras^1,2^.

Physically, both CCD and CMOS cameras accumulate a signal charge in each pixel proportional to the local illumination intensity. When the exposure is complete, a CCD camera transfers each pixel’s charge budget sequentially to a common output structure. This structure converts the charge to a voltage and sends it off-chip, so that most functions take place in the camera’s printed circuit board. Instead, in a CMOS imager, the charge-voltage conversion takes place in each pixel and most functions are integrated on the chip. Such difference in the readout technique has several implications in the capabilities and limitations of these two sensor architectures^3,4^.

To date, a new generation of sCMOS cameras approaches the imaging performance of a true low-light detector, with a low readout noise (1–2 e^-^) at extremely rapid readout rate (up to 560 MHz)^5^. However, the readout technique remains unchanged, and thereby individual pixels are still characterized by different offsets, variances and gains, so that they appear to flicker even when there are no expected incident photons^4,6^. The extra noise source, combined with other major sources such as readout and photon shot noise, reduces the image quality and impairs fast and quantitative imaging using sCMOS cameras^7,8^.

So far, various efforts have been made to minimize the influence of the noise sources correlated to acquisition devices, especially in low-light conditions^9–12^. However, the classic assumptions of white noise become invalid at low-photon counts for CMOS and sCMOS sensors^13^ (Supplementary Note 1). To fully address the CMOS-related noise, different methods have been implemented to estimate the detector’s response using either a camera calibration prior to acquisition^8,14,15^ or the statistical analysis of the processed data^16–18^. However, these methods do not effectively remove the camera noise in many practical cases, either because of a tradeoff between noise correction and detail preservation^15^ or the lack of a precise knowledge concerning the imaging system or the noise statistics^18,19^.

Here, we introduce a content-adaptive algorithm for the automatic correction of sCMOS-related noise (ACsN) for fluorescence microscopy. ACsN combines camera physics and layered sparse filtering to address the most relevant noise sources in a sCMOS sensor while preserving the fine details of the signal. In particular, contrary to other approaches, ACsN is based on a theoretical model that performs a joint estimation of the noise variance using frequency analysis, which results in a robust and efficient performance for input sequences with low-photon budgets. Furthermore, ACsN probes the intrinsic self-similarity in space and time of fluorescent specimens, achieving quantitative image restoration with substantially enhanced accuracy and runtime. Using this method, we have demonstrated significant improvements in both fluorescence microscopy images and their downstream analysis in a wide range of imaging conditions and modalities.

## Results

### ACsN algorithmic framework

**Fig. 1:**
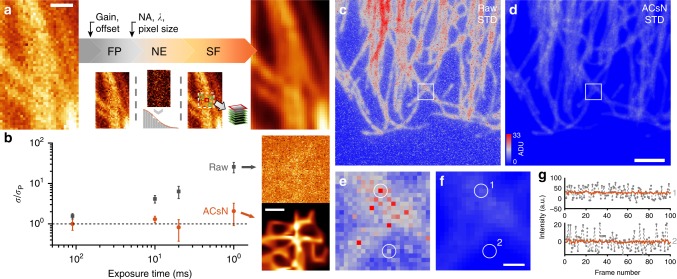
ACsN concept and performance. **a** Concept of the ACsN algorithm. The input image is scaled with the pixel gain and offset maps of the camera in order to remove the fixed-pattern noise (FP). Then, using the experimental parameters, the OTF boundary is calculated and used to produce a high-pass filtered image, from which the noise estimation (NE) is obtained. Finally, sparse filtering (SF) is performed to generate the denoised image. **b** Comparison of noise variations before (gray squares) and after (red circles) noise correction. All data were divided by the expected value for pure Poisson noise. The dashed line represents the ideal camera performance. To generate this plot three different sets of images of HeLa microtubules were used. The error bars represent the temporal standard deviation (STD) evaluated over 100 images. **c**, **d** Fluctuation maps, i.e., STD evaluated over 100 sCMOS images acquired at a 10-ms exposure time before (**c**) and after (**d**) ACsN denoising. Intensities are expressed in analog-to-digital units (ADU). **e**, **f** Zoomed-in images of the areas marked by the white squares in **c** and **d**, respectively. **g** Temporal fluctuation of the intensity values of the pixels corresponding to the circled areas (1 and 2) in **e** and **f**, respectively. The values from the original and denoised images are plotted in gray and red, respectively. Scale bars: 500 nm (**a**), 1 µm (**b**), 3 µm (**d**), 300 nm (**f**).

ACsN combines camera calibration, noise estimation and sparse filtering to correct the most relevant noise sources generated by a sCMOS camera (Fig. 1a and Supplementary Notes 1 and 2.1). In particular, ACsN first corrects the fixed-pattern noise using a map of the offset and gain of the sCMOS pixels. The presence of the fixed-pattern noise in sCMOS cameras generates in different pixels (*p*) a different number of photoelectrons from the same number of impinging photons (*S*_*p*_). This effect is proportional to the illumination level and can be modeled as a multiplicative factor *γ*_*p*_ applied to the parameter of the Poisson-distributed variable *S*_*p*_. At the same time, during the analog-to-digital (AD) conversion, the voltage produced by each pixel is read as the difference from a reference level, which represents the absence of light. In practice, this reference voltage is assigned a positive value that is responsible for a bias (*β*_*p*_) in the measured intensity values. Therefore, the acquisition of a sCMOS camera can be modeled by the equation:^20^

1 $$Z_p = \gamma _p{\mathrm{Pois}}\left\{ {S_p\left( \tau \right)} \right\} + N\left( {0,\sigma _R} \right) + \beta _p,$$

where *Z*_*p*_ is the value of the pixel *p*, *τ* the exposure time, and *N* (0, *σ*_*R*_) the Gaussian-distributed readout noise of mean *μ*_*R*_ = 0 and standard deviation *σ*_*R*_. Considering the practicality of fluorescence microscopy, in this model we have omitted the contribution of dark current, which can be disregarded for exposure times below 1 s, and the quantization noise due to the AD conversion, which is negligible compared to the readout noise^3,21^ (Supplementary Note 2.2).

Since the fixed-pattern noise depends only on the camera circuitry, *β*_*p*_ and *γ*_*p*_ can be estimated through a one-time calibration (see Methods). However, a careful assessment of both the Gaussian-distributed readout noise, *N*(0, *σ*_*R*_), and the fluctuation due to the Poisson-distributed photon shot noise, Pois{*S*_*p*_(*τ*)}, is necessary to obtain an accurate estimate of the underlying signal *S*_*p*_. To perform this assessment, we devised a noise model that allows for a joint estimation of the noise variance by analyzing the frequency response of the microscopy system. This is based on the fact that the Poisson distribution of the photon shot noise can be feasibly approximated by a Gaussian distribution when the photon flux is >3 photons per pixel^22^. In particular, the error introduced by approximating the Poisson variance, $\sigma _P^2$, with a Gaussian variance, $\sigma _G^2$, becomes <1% when the photon flux is more than 5 photons per pixel (Supplementary Note 2.3). Notably, the abovementioned conditions on the photon flux are usually satisfied for many applications in fluorescence microscopy^23,24^. Therefore, we consider the camera-related noise as the result of the sum of two independent Gaussian-distributed random variables, whose variance is $\sigma _N^2 = \sigma _R^2 + \sigma _G^2$. Such a distribution consists of a constant power spectral density, while the signals coming from the sample are contained within the optical transfer function (OTF)^25^. Therefore, we take advantage of the knowledge of the optical system to evaluate the pixel fluctuation outside the OTF, which is due to noise only, and then we use the value obtained to derive *σ*_*N*_ in the original image (Supplementary Note 2.3).

Next, the algorithm uses these noise statistics for a non-local assessment of the self-similarity of the sample and to perform collaborative sparse filtering on the input sequence. Unlike previous implementations of collaborative filtering, we adopted a layered approach that sequentially probes the image self-similarity in space and time in order to enhance noise correction without sacrificing accuracy and runtime. In brief, the filter decomposes the image in patches and sorts them into three-dimensional (3D) groups according to their similarity^26^. Then, it employs a 3D transform to process each group all at once. The denoising is performed by hard-thresholding and enhanced by the fact that, due to the similarity between the patches, the 3D transform results in an even sparser representation of the original patches, whereas the noise power spectrum remains constant^27^. Afterwards, the denoised patches are returned to their original locations to form an intermediate image. At this point, the collaborative filter is run a second time but replacing the hard-thresholding with a Wiener filter. The filter is performed using both the noisy and intermediate images and generates the final denoised image (Supplementary Note 2.4). It should be noted that the spatial variation of the noise across the image may affect the performance of the Wiener filter. However, this is considerably mitigated by the use of patch-based processing, which, compared to the whole image, enhances the intensity uniformity within individual patch groups, exhibiting a great stability against spatially variant noise^9^.

Finally, another collaborative filter is performed looking for similar patches also in the neighboring frames. This way, lingering noise can be further reduced taking advantage of the sample self-similarity in time while preserving the temporal resolution^18^ (Supplementary Note 2.5).

### Characterization of ACsN

Next, we characterized the performance of ACsN using both numerical and experimental data. Notably, ACsN collaborative filtering depends on the estimation of *σ*_*N*_, as well as on the choice of the parameters in the algorithm^28^, which were chosen in order to optimize both the noise correction and runtime (Supplementary Note 3.1). We observed that our strategy can significantly attenuate the detrimental effect of camera noise, avoiding loss of image resolution, especially in presence of highly spatially variant noise (Supplementary Note 3.2). Moreover, the camera noise can induce temporal fluctuations of the pixel values that are not related to the sample, thus affecting the quantitative analysis of time-lapse data. ACsN denoising reduces this effect by approximately one order of magnitude, with residual fluctuations comparable to that of an ideal camera (Fig. 1b–g and Supplementary Note 3.3). Furthermore, it should be noted that at low-photon counts, the sample’s details start to be comparable with the noise fluctuations and become harder to retrieve. Thus, the performance of image restoration is intrinsically related to the photon flux of the input image. Nonetheless, using both simulations and experimental data, we verified a robust ACsN noise correction at low-light levels down to 5–10 photons per pixel (Supplementary Note 3.4).

Furthermore, we validated the performance of ACsN under various sampling rates normally adopted for fluorescence microscopy. In practice, a sampling rate close to the Nyquist criterion represents a good tradeoff between signal to noise ratio (SNR) and detail preservation. Here, examining numerically and experimentally across a wide range of sampling rates, we demonstrated the viability of ACsN for low SNR with oversampling and no noticeable loss of signals with under-sampling (Supplementary Note 3.5).

Unlike natural images, fluorescent images of biological samples are highly specified, exhibiting precisely labeled molecular targets or structures in cells. Therefore, each fluorescent image usually features specific objects recurring across the field of view, which supplies sufficient non-local self-similarity to make the algorithm notably efficient for fluorescence microscopy. With numerical and experimental data, we characterized the dependence of the ACsN performance on the usage of self-similarity of an input image (Supplementary Note 3.6). Furthermore, as shown in the following, we quantitatively assessed a variety of non-biological and biological samples to verify the viability of the method, spanning various dimensionality, morphology, randomness and density, such as caliber targets, fluorescent particles, single molecules, microtubules, actin filaments, mitochondria, filopodia, lamellipodia, and small animals.

### Wide-field microscopy

Wide-field microscopy, especially total internal reflection fluorescence (TIRF) microscopy, is one of the most widely used techniques in cell imaging^29^. TIRF uses the phenomenon of total internal reflection of light at the glass/water interface in order to create an evanescent wave that propagates only for a few hundreds of nanometers across the coverslip. This allows the selective excitation of the fluorescent labels at the bottom of the sample (Supplementary Fig. 1a). However, in case of weak fluorescent emitters, low-light intensity or a short exposure time, sCMOS-related noise becomes severe and deteriorates image quality (Supplementary Fig. 1b). ACsN denoising can effectively reduce such contribution and recover the undistorted signals from the noise, allowing faster acquisition without compromising the underlying signal (Supplementary Fig. 1c, d).

**Fig. 2:**
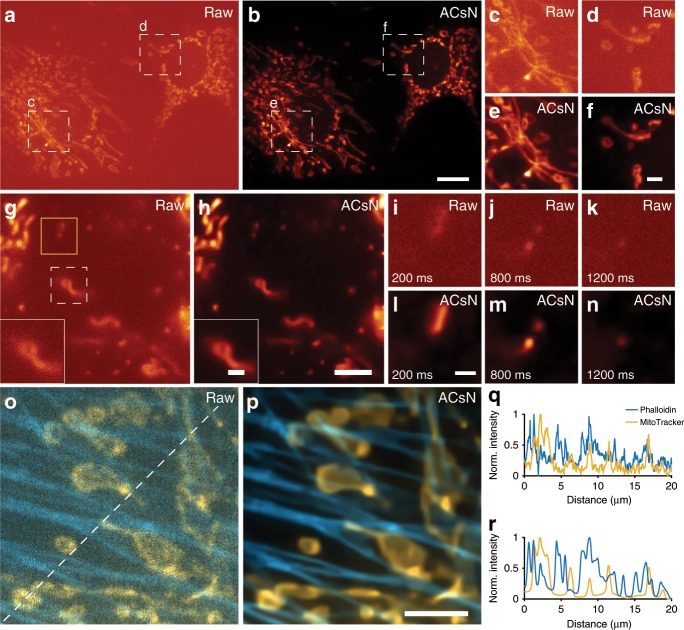
ACsN noise correction improves wide-field fluorescence microscopy. **a** Epi-fluorescence imaging of mitochondria in fixed bovine pulmonary artery endothelial (BPAE) cells at an exposure time of 1 ms. **b** The same image in **a** after ACsN denoising. **c**–**f** Zoomed-in images of the corresponding boxed regions in **a** and **b**. Quantitative results and analysis are reported in Supplementary Table 1. **g** Representative frame from a time-lapse of 100 images of mitochondria in live human embryonic kidney (HEK) cells recorded at 50 Hz (exposure time: 20 ms). **h** The corresponding representative frame of the image sequence (**g**) obtained after ACsN processing. The insets in **g** and **h** show zoomed-in images of the corresponding regions marked in the dashed white box in **g**. **i**–**n** Zoomed-in images of the corresponding regions marked in the solid yellow box in **g** at different time points of 200 ms (**i**, **l**), 800 ms (**j**, **m**), and 1200 ms (**k**, **n**). **o**, **p** Dual-color image, respectively, before (**o**) and after (**p**) ACsN denoising of F-actin (cyan) and mitochondria (orange) in fixed BPAE cells obtained by TIRF microscopy with an exposure time of 2 ms. **q**, **r** Cross-sectional intensity profiles of (**o**) and (**p**) along the corresponding dashed line in **o**, respectively, showing substantially denoised and better resolved cellular structures. Scale bars: 10 μm (**b**), 3 μm (**f**), 4 μm (**h**, **p**), 1 μm (**h**, inset) and (**l**).

We demonstrated ACsN denoising of wide-field microscopy in both epi-fluorescence and TIRF configurations using various fixed, live and multi-color sub-cellular samples, including microtubules (Fig. 1 and Supplementary Fig. 1), mitochondria (Fig. 2 and Supplementary Movies 1 and 2), and F-actin (Fig. 2). The use of ACsN can maintain the same image quality with a shorter exposure time (i.e., better temporal resolution) and a lower excitation level (i.e., less photo-damage). The performance is, thus, limited primarily by the photo-physics of the fluorescent emitters. Using quantitative metrics, we showed that the method can recover wide-field images with a photon budget two orders of magnitude lower with no loss of image quality (Supplementary Table 1).

### Deconvolution and light-field microscopy

Image deconvolution is widely used in optical microscopy, from the restoration of low-quality images to the improvement of super-resolution techniques^30^. However, noise can easily degrade the performance of many common algorithms by producing deconvolution artifacts. Instead, we observed a remarkable reduction of such artifacts in deconvolved images by employing ACsN denoising prior to different methods based on Richardson–Lucy algorithm^31^, machine learning^32^, and radial fluctuation^33^ (Supplementary Note 4.1). The enhancement of image restoration is reflected also by an improvement of the global image quality, evaluated using metrics such as the Resolution Scaled Pearson’s coefficient (RSP)^34^. For example, combining ACsN and radial fluctuation, we generated super-resolution images with a better RSP value at a temporal resolution up to two orders of magnitude higher than currently reported^33^ (Supplementary Fig. 2).

**Fig. 3:**
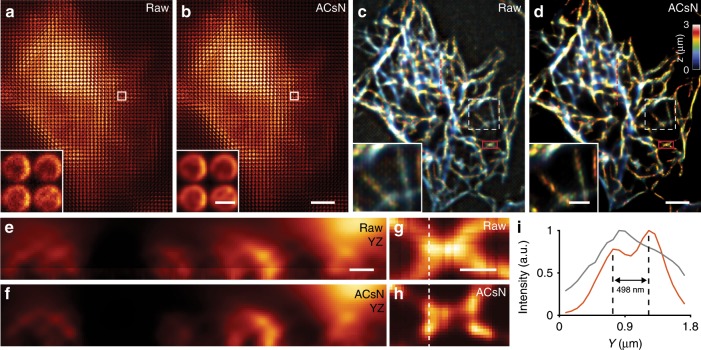
ACsN denoising improves the quality of 3D reconstruction in light-field microscopy. **a**, **b** Raw light-field images of microtubules in a HeLa cell before (**a**) and after (**b**) ACsN processing. Insets show the zoomed-in microlens images of the corresponding boxed regions, where noise has been substantially reduced as seen in **b**. **c**, **d** Three-dimensional (3D) reconstructed images obtained from **a** and **b**, respectively. The depth information is coded according to the color scale bar. Insets show the zoomed-in images of the corresponding white dashed boxed regions, where better image quality and improved 3D resolution are observed after ACsN denoising. **e**, **f** Cross-sections on the YZ plane corresponding to the red dashed lines in **c** and **d**, respectively, where microtubule structures are better resolved with reduced artifacts using ACsN. **g**, **h** Zoomed-in images of the red solid boxed regions in **c** and **d**, respectively, at *z* = 1.4 μm, where microtubule structures are better resolved using ACsN. **i** Cross-sectional profiles of (**g**, gray) and (**h**, red) corresponding to the white dashed lines in **g**, **h**, respectively. Filaments covered by non-fluorophore-associated background noise are resolved using ACsN. Scale bars: 8 µm (**b**, **d**), 800 nm (**b**, inset), 3 µm (**d**, inset), 1 µm (**e**, **g**).

Image deconvolution is also at the basis of three-dimensional reconstruction in light-field microscopy (LFM). LFM employs a microlens array in a microscopy system to obtain both the two-dimensional (2D) spatial and 2D angular information of the incident light, allowing for computational reconstruction of the full 3D volume of a specimen from a single camera frame^35^. However, the deconvolution-based reconstruction process is highly sensitive to the SNR, especially due to LFM’s wide-field, volumetric, and fast imaging scheme. For this reason, the use of ACsN to correct the noise in the raw images (Fig. 3a, b) results in clearly noticeable improvement in the 3D light-field reconstructions (Fig. 3c, d). Indeed, the presence of the noise leads to the miscalculation of the 3D object or the propagation of non-fluorophore-associated peaks. The former affects the sampling along the axial dimension and can result in an uneven axial resolution (Fig. 3e, f). The latter produces additional background that covers the fluorescence signal, impairing also the lateral resolution (Fig. 3g–i). Using ACsN, both deficiencies can be mitigated, resulting in substantially improved 3D volumetric rendering of cellular structures.

### Single-molecule localization microscopy

**Fig. 4:**
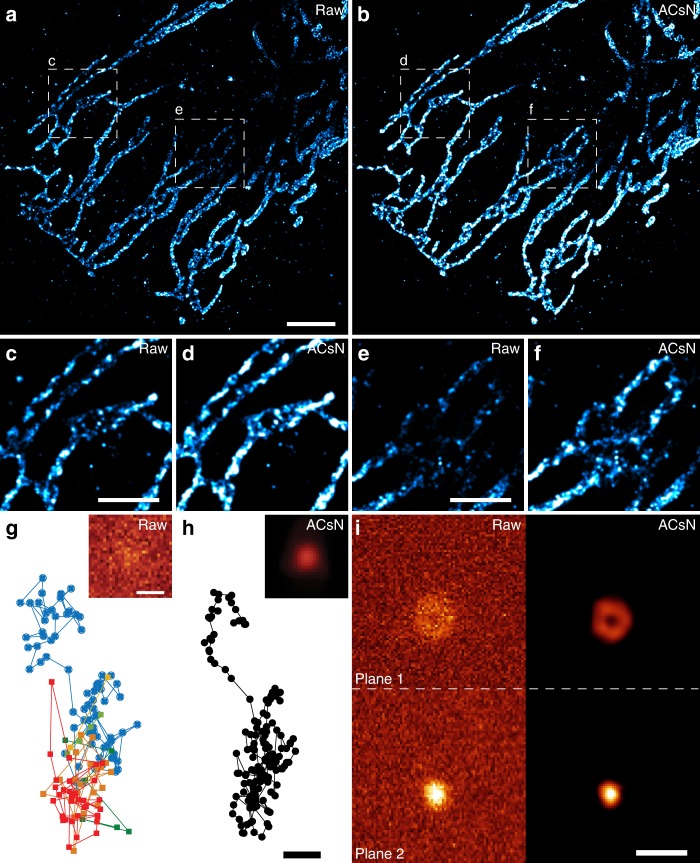
ACsN improves localization performance in STORM and single-particle tracking. **a** STORM image of mitochondria in a fixed HeLa cell (RSP: 0.81, RSE: 40.6). **b** STORM image reconstructed after ACsN denoising of raw single-molecule data of **a** (RSP: 0.85, RSE: 36.7). In both cases, 5000 single-molecule frames were used. Representative frames of the raw data before and after denoising are shown in Supplementary Fig. 3. Quantitative image analysis with NanoJ-SQUIRREL assessed an improvement of both RSP ( + 0.04) and RSE (−3.9) values in **b** compared to **a**. It is observed that the number of localizations in **b** is increased in comparison with **a**, which leads to a better contrast in the former and to the appearance of features not visible in the latter (**c**–**f**). **g** Single-particle tracking of a fluorescent bead recorded with a 1 ms exposure time. A representative frame is shown in the inset. Each color corresponds to one of the six different tracks detected. **h** Single-particle tracking of the same bead in **g** after ACsN denoising (inset). The improved SNR yields a better localization accuracy, which results in a single, smooth trajectory (black line). **i** Representative frame for biplane single-particle tracking at 1 kHz frame rate (exposure time: 1 ms) before (left) and after (right) ACsN denoising. Scale bars: 4 µm (**a**), 2 µm (**c**, **e**, **i**), 1 μm (**g**, inset), 250 nm (**h**).

To validate the feasibility of ACsN for single-molecule localization microscopy (SMLM)^36^, we performed STORM imaging of mitochondria in HeLa cells (Supplementary Fig. 3). The effect of sCMOS-related noise in single-molecule localization can be seen in two aspects: the presence of false negatives, due to the loss of weakly emitting molecules covered by noise (Supplementary Fig. 3c, d), and the presence of false positives, due to the hot pixels or simply the noise distribution (Supplementary Fig. 3e, f). Removing the noise from the raw single-molecule data allows for suppression of both types of localization errors, resulting in significantly improved STORM image quality and metrics such as the RSP and the Resolution Scaled Error (RSE)^34^ (Fig. 4a, b). Also, such improved efficiency of localization leads to a better contrast and the appearance of features not clearly visible in the reconstruction without denoising (Fig. 4c–f). Furthermore, the reduction of pixel fluctuations unrelated to the sample permits to obtain a map of the fluorophores’ blinking rate that can be used to alleviate the effects of imperfect labeling (Supplementary Fig. 4).

Like single-molecule imaging, the localization precision in single-particle tracking (SPT) is closely related to the number of photons detected. Therefore, one critical factor affecting the performance of SPT is the SNR of the image data^37^. We showed that ACsN can be used to minimize the localization errors responsible for misidentification of particles and erroneous trajectories (Fig. 4g, h and Supplementary Movie 3). This SNR improvement results in a better particle localization accuracy, i.e., a better estimation of the bead’s lateral displacement with sub-pixel sensitivity. This can be of great use also in biplane SPT, where the accuracy of the 3D tracking depends on the quality of the out-of-focus image^38^ (Fig. 4i, Supplementary Movie 4, and Supplementary Note 4.2).

### Fluorescence microscopy with low-cost CMOS cameras

**Fig. 5:**
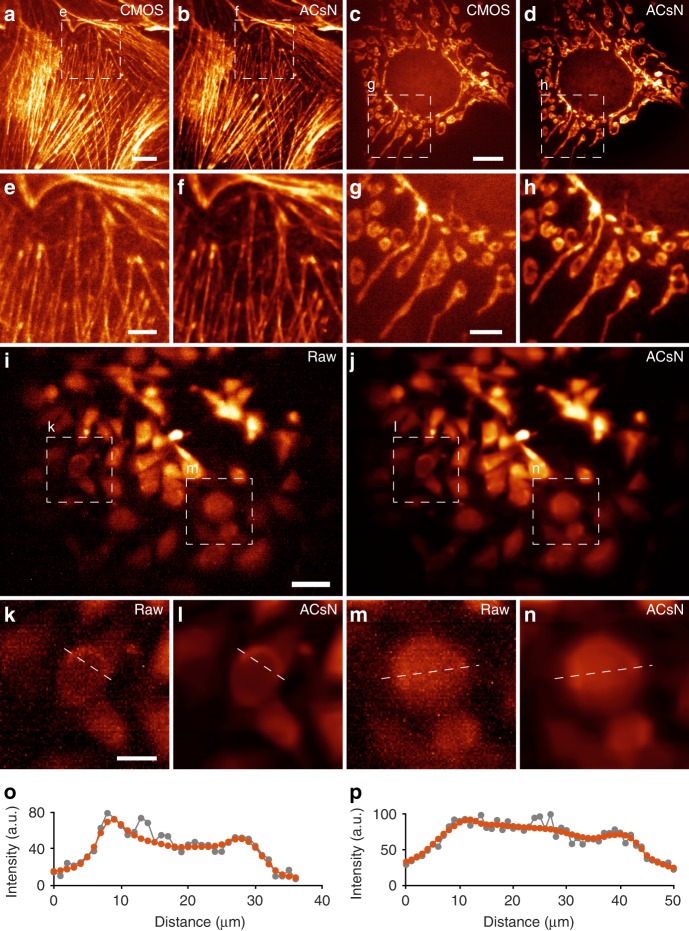
ACsN improves fluorescence microscopy with low-cost CMOS cameras. **a** TIRF image of F-actin in a fixed BPAE cell, taken at a frame rate of 38 Hz (exposure time: 26 ms). **b** The same image in **a** after ACsN denoising. **c** Epi-fluorescence imaging of mitochondria in a fixed bovine pulmonary artery endothelial (BPAE) cell, taken at a frame rate of 38 Hz (exposure time: 26 ms). **d** The same image in **c** after ACsN denoising. **e**–**h** Zoomed-in images corresponding to the boxed regions in **a**–**d**, showing the improvement of image quality after ACsN denoising. In particular, such improvement is comparable to the images taken with sCMOS sensors, as shown in Supplementary Figs. 5 and 6. **i**, **j** Images of GFP-stained calcein in live Adipocytes (lipocytes) taken with low-cost CMOS for miniaturized microscopy before (**i**) and after (**j**) ACsN denoising. The data were taken by immersing a miniscope in live-cell culture. **k**–**n** Zoomed-in images of the corresponding boxed regions in **i** and **j**. **o**, **p** Plots of the cross-sectional intensity profiles of cellular structures before (gray) and after (red) ACsN denoising along the dashed lines in **k**, **l** and **m**, **n**, respectively. Scale bars: 10 μm (**a**, **c**), 4 μm (**e**, **g**), 50 μm (**i**), 20 μm (**k**).

Recently, the advances of high-end industrial-grade CMOS cameras have sparked the interest of the scientific community at the possibility to approach the performance of sCMOS cameras at a more affordable price^39–42^. It has been shown that such CMOS cameras can be utilized for SMLM imaging^41,42^. However, the lower quantum efficiency and the higher readout noise limit the image quality and the general usability for quantitative biomedical research in many areas. Addressing the challenge with a proper denoising strategy would provide a critical and timely solution to transform the industrial-grade cameras for broader imaging applications. Here, we first implemented ACsN with a high-end industrial-grade camera for wide-field microscopy using both epi- and TIRF illumination (Fig. 5a–h). In both configurations, ACsN denoising substantially improved the image quality, achieving prominent agreement with the images obtained by the sCMOS camera (Supplementary Figs. 5 and 6, and Supplementary Table 2).

The single-photon-excitation-based miniaturized microscope, or miniscope, has been developed to perform wide-field calcium imaging in freely behaving animals^43–45^. The required miniaturization was achieved by replacing compound objective lenses with a gradient-index (GRIN) rod lens, which offers several advantages, including low cost, light weight, and relatively high-numerical aperture. These features of the miniscope enable minimally invasive imaging of a significant volume of the brain with a cellular-level resolution during complex behavioral, cognitive and emotional states^46–48^. However, the low-cost CMOS sensor (MT9V032C12STM, ON Semiconductor, price ~$15) currently adopted yields a poor image quality in order to obtain a relatively high imaging speed, which can be severely restrictive for broader applications in cell imaging. Here, we validated the feasibility of ACsN for the miniscope sensor by performing single-photon-excitation-based, wide-field imaging of GFP-stained calcein in live Adipocytes (Fig. 5i–p).

### Selective plane illumination microscopy

In contrast to wide-field microscopy, selective plane illumination microscopy (SPIM) illuminates the sample with a sheet of light perpendicular to the direction of observation. This avoids unnecessary illumination, permitting an unparalleled long-term imaging of dynamic biological specimens^49–51^. Lattice light-sheet microscopy (LLSM) further optimizes the optical system by illuminating the sample with multiple plane waves that sculpt a propagation-invariant optical lattice^52^. However, while new strategies are being investigated to deal with sample-related issues^53,54^, camera noise remains the most relevant limitation to SPIM and LLSM imaging capabilities due to their relatively low-background signal.

**Fig. 6:**
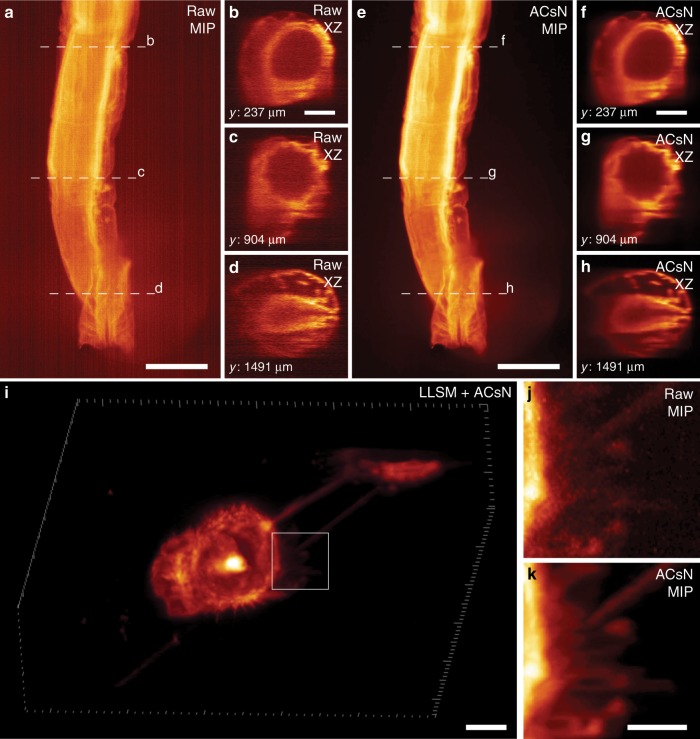
ACsN processing of volumetric data obtained with SPIM and LLSM. Maximum intensity projections (MIP) of SPIM images of a fluorescently labeled adult brine shrimp before (**a**) and after (**e**) ACsN denoising. Orthogonal views along the XZ plane of the raw (**b**–**d**) and denoised (**f**–**h**) volumetric scans at *y* = 237 mm (**b**, **f**), *y* = 904 mm (**c**, **g**), and *y* = 1491 mm (**d**, **h**). Slices along the XY and YZ plane have been provided in Supplementary Fig. 7. **i** Three-dimensional rendering of live human lung cancer cells (NCI-H1299 NSCLC) acquired with LLSM and processed with ACsN denoising. Zoomed-in images of the area corresponding to the white box in **i** before (**j**) and after (**k**) ACsN denoising. The corresponding time-lapse sequence has been provided in Supplementary Movies 6 and 7. MIP images and representative slices are depicted in Supplementary Figs. 9 and 10. Scale bars: 400 μm (**a**, **e**), 100 μm (**b**, **f**), 10 μm (**i**), 4 μm (**k**).

We first demonstrated that ACsN denoising can overcome this limitation by performing a SPIM volumetric scan of a fixed brine shrimp. Here, we enhanced the self-similarity using 3D sparse filtering along the scan direction. After ACsN processing, we observed that noise-canceling makes the sample’s details stand out better in each individual slice (Supplementary Fig. 7). In particular, the correction of the fixed-pattern noise is especially noticeable in the maximum intensity projection images (Fig. 6a, e and Supplementary Movie 5). In addition, it is remarkable to observe a clear improvement in the orthogonal cross-sections of the scanned volume (Fig. 6b–d, f–h), allowing for a better assessment of the sample’s 3D structures.

To validate ACsN processing for LLSM, we first imaged fixed skin cells stained for Keratin with EGFP at different exposure times (5, 10, and 20 ms) using a constant laser illumination power of 27 mW (measured at the back focal plane of the illumination objective). These images were acquired using the sample scan mode and, accordingly, the slices had to be deskewed to retrieve the original positions (see Methods). We performed such operation before ACsN denoising in order to utilize the self-similarity along *z* for 3D sparse filtering. We observed that the image quality can be well maintained by denoising even after a fourfold reduction of the exposure time (Supplementary Fig. 8 and Supplementary Table 3).

Furthermore, we demonstrated ACsN image restoration of time-lapse live-cell LLSM imaging. First, we imaged live human lung cancer cells (NCI-H1299 NSCLC) in the sample scan mode with intervals of 18.4 s over more than 30 min (Fig. 6i–k, Supplementary Fig. 9, and Supplementary Movies 6 and 7). As stated above, the sample scan mode requires deskewing of the volumetric slices, which increases the size of the dataset and, then, the processing complexity. In contrast to the previous case, however, for time-lapse imaging we were able to utilize the temporal self-similarity, which yields a more efficient noise correction compared to the volumetric one^55^. Therefore, we denoised the time-lapse volumetric scans by processing the corresponding temporal stacks of each individual slice. This way, ACsN could be used before deskewing, effectively preserving the denoising performance while saving the computational time (Supplementary Fig. 10). Next, we observed the movement of endogenous F-actin in live mouse embryonic fibroblasts using LLSM in the sheet scan mode (see Methods). Notably, this mode does not produce any shift between the slices, and the volumetric information can be retrieved without deskewing (Supplementary Fig. 11**)**. In particular, the movement of filopodia all around the cell can be observed with higher clarity after denoising (Supplementary Movie 8).

## Discussion

Nowadays, many imaging methods rely on computational analysis to extract additional information from digital images. However, even modest noise levels can introduce errors that propagate through the processing pipeline, deteriorating the quality of the final results. Here, we proposed a denoising method designed for fluorescence microscopy. This is based on a theoretical noise model that effectively considers multiple noise sources and allows for a joint estimation of the noise variance using high-frequency analysis. The algorithm is composed of three components intrinsically linked to each other and critical for the feasibility: camera calibration, noise estimation, and sparse filtering. The camera calibration removes the spatially correlated fixed-pattern noise and allows for a physics-based estimation of the white noise. Such estimation reveals the spatial fluctuation of the noise across the image, essential for the subsequent non-local sparse filtering process. Finally, the use of patch-based sparse filtering enhances intensity uniformity, facilitating the correction of spatially varying noise.

During the last years, patch-based algorithms have been extensively adopted in the processing of natural images. However, the performance of the existing methods can be severely affected by a low SNR^56^, hindering a real breakthrough in fluorescence microscopy^9^. On the contrary, our implementation has significantly advanced the approach by employing the inherent characteristics of the imaging system and the fluorescent biological samples. This improves ACsN noise correction by up to two orders of magnitude in terms of the mean square error (MSE) compared to general-purpose sparse filtering (Supplementary Note 5). Furthermore, compared to the existing approaches that address the noise correction of sCMOS cameras for wide-field microscopy^15^, ACsN denoising shows a sevenfold improvement in the MSE and up to two orders of magnitude improvement in runtime, mainly because of the new noise model and algorithmic scheme (Supplementary Notes 5 and 6).

Unlike other denoising methods for microscopy that were implemented for specific cases^15,57,58^, we demonstrated the broad applicability of ACsN by showing its performance in diverse experimental conditions, with different sensors, and for a wide range of applications (Supplementary Table 4). In addition, we have also demonstrated that the noise correction of sCMOS images can result in a major improvement of the downstream analysis. Finally, by processing time series of both fixed and live samples, we observed a substantial reduction of pixel fluctuations and, thus, of the measurement errors, allowing for accurate, quantitative study of time-lapse data. In this regard, users should be aware that such errors may not be completely removed, but we observed that they are reduced to the error level or lower of an ideal camera. This allows for an acceptable denoising accuracy even at low-light intensity, down to 5–10 photons per pixel. However, the determination of a minimum threshold for denoising reliability under a lower photon flux may vary depending on the validity of the noise model, as well as the camera, specimen or imaging technique used. For this reason, we recommend to calibrate and test the algorithm before applying it to any new type of data. To help with this task, ACsN also provides an evaluation of the restoration quality that allows users to identify images where denoising may not be accurate (Supplementary Note 7). Further guidelines for the usage of ACsN are provided in Supplementary Note 8.

Lastly, the algorithm is accessible for future developments to meet broader imaging conditions like the implementation of features to handle multidimensional spatiotemporal data, the use of GPU parallel processing, and the optimization of image restoration for low-cost sensors. We anticipate that this tool can be useful for any type of CMOS/sCMOS-based imaging where quantitative analysis, fast runtime, and low-photon count are desired.

## Methods

### Camera calibration

To calibrate the pixel-dependent offset of the CMOS cameras used in this work, we disabled the automatic pixel correction to avoid automatic replacement of hot pixels by the average of the neighboring pixels. Then, we recorded a series of dark images and calculated the temporal mean for each pixel. We used 10,000 frames for the ORCA-Flash-4.0 sCMOS (Hamamatsu Photonics) and PCO.Edge, and 5000 frames for the Grasshopper 3 CMOS camera (GS3-U3–51S5M-C, FLIR Imaging) and the Miniscope’s CMOS sensor (MT9V032C12STM, Aptina-On Semiconductor). The amplification gain was estimated from multiple sets recorded at different illumination intensities ranging from ~20 to 500 photons per pixel. The gain for each pixel was calculated using the relation:

2 $$g_i = \arg {\mathrm{min}}\mathop {\sum}\limits_{k = 1}^K {\left( {\left( {v_i^k - {\mathrm{var}}_i} \right) - g_i\left( {D_i^k - o_i} \right)} \right)^2} ,$$

where *K* is the total number of illumination levels acquired, *k* is the *k*th illumination sequence, $D_i^k$ stands for the mean count in analog-to-digital units (ADU) obtained from temporal averaging of all frames that are acquired during illumination sequence *k* in pixel *i*, *o*_*i*_, and var_*i*_ are the mean and variance values for pixel *i*, and $v_i^k$stands for the temporal variance of the ADU counts for illumination sequence *k* in pixel *i*^8^.

### Quality metrics

To quantify the quality of image restoration for wide-field images we used three popular metrics: mean square error (MSE), peak signal to noise ratio (PSNR), and structural similarity index (SSIM). The MSE is an element-wise difference between two input images, where the ideal value is zero. The MSE is computed by squaring the difference of corresponding pixels in each image *X* and *Y* and taking the mean of the squared differences:

3 $${\mathrm{MSE}}\left( {X,Y} \right) = \frac{1}{N}\mathop {\sum}\limits_{p = 1}^N {\left( {X_p - Y_p} \right)^2} .$$

The PSNR is derived from the MSE, and indicates the ratio of the maximum pixel intensity to the power of the distortion.

4 $${\mathrm{PSNR}}(X,Y) = 10 \cdot \log _{10}\left( {\frac{{{\mathrm{max}}(Y)^2}}{{{\mathrm{MSE}}(X,Y)}}} \right).$$

The SSIM metric is widely adopted in image processing to evaluate image fidelity from an objective point of view^59^. This index is an alternative to error summation methods (like SNR or MSE) and it is supposed to give more information about image distortion by the computation of local image structure, luminance, and contrast into a single local quality score. In this metric, structures are patterns of pixel intensities, especially among neighboring pixels, after normalizing for luminance and contrast:

5 $${\mathrm{SSIM}}(X,Y) = \frac{{\left( {2\mu _X\mu _Y} \right)\left( {2\sigma _{XY}} \right)}}{{\left( {\mu _X^2 + \mu _Y^2} \right)\left( {\sigma _X^2 + \sigma _Y^2} \right)}}$$

where *μ*_*X*_, *μ*_*Y*_, *σ*_*X*_, *σ*_*Y*_, and *σ*_*XY*_ are the local means, standard deviations, and cross-covariance for images *X* and *Y*. As the human visual system is good at perceiving structure, the SSIM quality metric agrees more closely with the subjective quality score.

To assess the quality improvement of image deconvolution and STORM reconstructions, we used NanoJ-SQUIRREL to evaluate the resolution scaled error (RSE) and the resolution scaled Pearson’s coefficient (RSP)^34^. The RSE is a metric describing the root mean square error between a reference image and the resolution- and intensity-scaled super-resolution image. It exhibits intensity-dependence and as such, it is sensitive to any non-linear intensity scaling. Lower values indicate better agreement. Instead, the RSP describes the Pearson correlation coefficient between the reference image and the resolution scaled image. This metric is independent of image intensity and normalized between −1 and 1, where 1 represents the ideal agreement.

### Wide-field epi-fluorescence, TIRF, and STORM imaging

All the epi-fluorescence, TIRF, and STORM acquisitions were performed on an inverted optical fluorescent microscope (Nikon Ti-U). A 647 nm laser (MPB) and a 405 nm laser (OBIS) were used to excite and switch the reporter fluorophores (Alexa 647). The lasers were coupled into an optical fiber (Thorlabs) and sent to the microscope. An oil-immersion objective (NA 1.45, 100x Nikon CFI-PLAN Apo Lambda, Nikon) was used to enable sub-cellular structure imaging. A lens with focal length of 20 cm was used to focus the laser beam in different incident angles. TIRF illumination was enabled to reduce the background introduced by the structures deep in the sample and enhance the contrast of the acquired frames. The emitted fluorescence was collected with a sCMOS camera (Hamamatsu ORCA 4.0 V3) at the right-side camera port and with an industrial-grade CMOS camera (GS3-U3–51S5M-C, FLIR Imaging) at the left-side camera port.

HeLa cells (ATCC, Manassas, VA) were plated on a 35 mm MatTek glass-bottom plate and incubated at 37°C with 5% CO_2_. After 16 h, the cells were fixed for 10 min at 37°C using 4% formaldehyde (Electron Microscopy Sciences) resolved in phosphate-buffered saline (PBS). The cells were then washed and incubated for an hour in the 1 mL blocking solution 1% (vol/vol) bovine serum albumin (BSA) (Santa Cruz Biotechnologies) and 0.25% (vol/vol) Triton X-100 prepared in PBS). A focus lock was used to stabilize the microscopic stage during the image acquisition. The infrared laser was separated by a beam splitter into two beams: one was reflected by the glass-oil interface between the objective lens and the microscope slide and then induced into a Thorlabs CMOS camera; the other was directed into the camera. The distance between two beam spots on the camera was therefore sensitive to the position of the stage (i.e., the distance between the objective lens and the microscope slide), and this information was fed back to a piezo actuator (Mad City Labs) by the STORM software to correct the axial drift of the stage.

Microtubules were stained with mouse anti-Tom20 (Santa Cruz Biotechnologies F10, SC-17764) for 2 h while gently shaking at room temperature. The second antibody was labeled with 1 mg/mL AlexaFluor 647-conjugated AffiniPure Goat Anti-Mouse IgG (Jackson ImmunoResearch), followed by a one-hour incubation at room temperature. A 5-min-each triple-washing step was conducted with PBS after each staining and labeling step. The cells were placed in imaging buffer (1 M Tris pH 8.0, 5 M NaCl, 1.0 N HCl, cyclooctane (COT), cysteamine (MEA), 50% glucose) before imaging.

HEK 293 (ATCC, CRL-1573) cells used for live-cell imaging were kindly provided by the Dahlman lab. Cells were cultured at 5% CO_2_ in Dulbecco’s modified Eagle’s medium (DMEM; Gibco) supplemented with 10% fetal bovine serum (FBS) and 1% penicillin-streptomycin. For mitochondrial imaging, cells were treated with MitotackerTM Green (Invitrogen) at 100 nM for 30 min.

Commercially available prepared slides (FluoCells slide #1, ThermoFisher) were used to image mitochondria and F-actin of bovine pulmonary artery endothelial (BPAE) cells. Mitochondria were labeled with red-fluorescent MitoTracker Red CMXRos and F-actin was stained using green-fluorescent Alexa Fluor 488 phalloidin.

In STORM image acquisition, the two lasers were activated simultaneously, which enabled stochastic photoswichable fluorescence emitted from the samples. The power of the 647 nm laser was set to 50 mW (measured on the coverglass surface) in order to allow the fluorescent molecules to switch on and off at a high rate for acquisition at a frame rate of 50 Hz.

### Light-field microscopy

We constructed a custom high-resolution light-field microscope (HR-LFM) on an epi-fluorescence microscope (Nikon Eclipse Ti-U) using a 100 × objective (Nikon CFI-PLAN 100×, 1.45 NA)^60^. The sample stage was controlled by a nano-positioning system (Prior). The samples were excited with 488-nm, 561-nm and 647-nm lasers (MPB Communications). The corresponding emitted fluorescence was collected using dichroic mirrors (respectively, T495lpxr, T560lpxr, and T660lpxr, Chroma) and emission filters (respectively ET525/50, Chroma; FF02-617/73, Semrock; ET700/75, Chroma). The microlens array (MLA), model S125-F30 by RPC Photonics, was aligned in a five-axis kinematic mount (K5X1, Thorlabs). The light field was imaged using a 1:1 relay lens (Nikon AF-S VR Micro-Nikkor 105 mm f/2.8G IF-ED) and recorded on a scientific complementary metal-oxide-semiconductor (sCMOS) camera (ORCA-Flash4.0 V3, Hamamatsu).

In the set-up, the MLA forms a defocused imaging relationship as 1/*a* + 1/*b* > 1/*f*_ml_, where *a* and *b* denote the distances to the native image plane and the camera sensor, respectively, and *f*_ml_ is the focal length of the MLA^60^. To reconstruct the volumetric data, the Fresnel propagation of light by the distances of *a* and *b*, i.e., a defocused point spread function (PSF), was established using the scalar diffraction theory. Specifically, the final intensity image *O*(**x**″) at the camera plane is described by $O\left( {{\mathbf{x}}^{\prime\prime} } \right) = {\int} {\left| {h\left( {{\mathbf{x}}^{\prime\prime} ,p} \right)^2} \right|} g\left( {\mathrm{p}} \right)dp$, where ${\mathbf{x}}^{\prime\prime} = \left( {x_1^{\prime\prime} ,x_2^{\prime\prime} } \right) \in R^2$ represents the coordinates $\left( {x_1^{\prime\prime} ,x_2^{\prime\prime} } \right)$ on the camera plane, and $p \in R^3$ is the position of a point source in a volume in the object domain, whose combined intensities are distributed according to *g*(*p*). $h\left( {{\mathbf{x}}^{\prime\prime} ,p} \right)$ represents the complex-valued PSF, which considers, sequentially, the light propagation through the high-NA objective, Fresnel propagation of light by the distance of *a*, modulation induced by the MLA, and another Fresnel propagation to the camera plane by the distance of *b*. In practice, considering the discrete model, $h\left( {{\mathbf{x}}^{\prime\prime} ,p} \right)$ is represented by the measurement matrix *H*, whose elements *h*_*kj*_ describe the projection of the light arriving at the pixel *O*(*j*) of the camera from the *k*th voxel *g*(*k*) in the object space. The volumetric information was then reconstructed employing the wave-optics model based on an inverse-problem deconvolution framework^60–62^.

### Single-particle tracking

We tracked the Brownian motion of 1-μm fluorescent beads (ThermoFisher T7280) in deionized water using a commercial microscope for single-molecule localization (Vutara 352, Bruker). We imaged the sample using a water immersion objective (60×, NA 1.2) and recorded the particle motion at a frame rate of 1 kHz (Hamamatsu ORCA 4.0 V3). To prepare the sample, we applied 5 µl of a diluted Tetraspeck bead suspension (1:100) on top of a clean microscope slide. We covered the sample with a coverslip and sealed it with nail polish.

### Miniaturized microscopy

We used a lab-built miniaturized imaging system based on the open-source miniscope protocol^63,64^. The illumination is provided by a 488 nm LED (LXML-PB01–0030, Lumileds), an excitation filter (FF01–480/40, Semrock), and a collimating lens (45549, Edmund Optics). The light from the sample is collected by a GRIN lens (0.5 NA, GT-IFRL-200-inf-50-NC, Grintech), reflected by a dichroic mirror (FF506-Di03, Semrock) and imaged by an aspheric lens (D-ZK3, Thorlabs) onto a CMOS sensor (MT9V032C12STM, ON Semiconductor).

### Selective plane illumination microscopy

For selective plane illumination microscopy, we used a commercial set-up (Zeiss Lightsheet Z.1). The system is equipped with dual PCO.Edge sCMOS cameras for multiview acquisition, four laser lines, and a CO_2_ incubator with temperature and humidity control. We used the system to image an adult brine shrimp or artemia (Carolina Bioscience) fixed in paraformaldehyde and stained with Eosin Y. We illuminated the sample with two 5 × / 0.1 NA objectives and detected the emission fluorescence with a 5 × / 0.16 NA. The detection zoom inside the microscope was set to 1.4×, so that the total magnification at the camera plane was 7×.

### Lattice light-sheet microscopy

Lattice light-sheet microscopy images were acquired using a 3i Lattice Light Sheet microscope. Here, lasers are individually expanded in the laser launch to 2.5 mm, collimated and aligned to be co-linear. All lines pass through an Acousto-Optic Tunable Filter (AOTF). Frequency modulation of the AOTF regulates the degree of higher order light that is transmitted, thus regulating the laser power input into the system. Once in the Lattice Light Sheet optical path, a set of cylindrical lenses expands the 2.5-mm input beam in X to 25 mm to uniformly illuminate a stripe on the spatial light modulator (SLM). The SLM is programmed to display binary images of user generated multi-Bessel patterns generating an optical lattice of Bessel beams. The Bessel beam is projected onto an annular mask, which filters the zeroth order, removes artifacts and lengthens the sheet. The mask is serially conjugate to Z and X galvo mirrors, as well as the rear pupil of the excitation objective, allowing the light sheet to be translated in y and z and to rapidly oscillate in x for the dithered mode of operation. The beam is focused through the illumination objective to create a pattern of the Bessel beams at the sample plane that is conjugate to the projection off of the SLM. This pattern is dithered by the X galvo to form the sheet of illumination that is observed by the sCMOS camera (ORCA-Flash4.0 v2, Hamamatsu), through the detection objective. The 25 × detection objective, in conjunction with the 500-mm tube lens, gives a 62.5 × magnification at the camera.

The volumetric data acquisition can be performed in two modalities: sample scan or sheet scan. In sample scan mode the stage moves while the light sheet and the objectives remain stationary. This mode allows to scan big areas but, since the objective is tilted at an angle with respect to the axis of stage movement, the scan produces a lateral offset between images from neighboring *z* planes. Therefore, these images have to be shifted (or deskewed) in post-processing to retrieve the original positions. In sheet scan mode, instead, the light sheet and objective are moved in tandem so that there is no offset between the volumetric slices and no deskewing operation is needed.

HaCaT keratinocytes were generously provided by Kowalczyk Lab at Emory University. They were cultured in DMEM (Corning, Tewksbury, MA) supplemented with 10% fetal bovine serum and 1% Antibiotic/ antimycotic. Cells grown on 5-mm coverslips were transfected according to manufacturer’s instruction with Viromer RED (OriGene, Rockville, MA). Briefly, plasmids were incubated with Viromer RED transfection reagent and buffer for 20 min at room temperature. This plasmid/reagent mix was then added to cells in culture dishes. Cells were then fixed 24 h after transfection with 4% PFA for 15 min. The mCherry-VAPB (human) plasmid construct was purchased from Addgene (Plasmid #108126).

Mouse embryonic fibroblasts (MEFs) were generously provided by Chan lab at Caltech. They were plated onto 5 mm matrigel-coated coverslips 24 h before experimentation, at about 60–70% confluence. Cells were grown in DMEM + 10% FBS. The day of the experiment, the medium was swapped with imaging medium (phenol red-free DMEM with HEPES, Gibco, Catalog #: 21063–029) along with SiRActin dye (diluted to a 1 µM concentration; Cytoskeleton Inc, Cat. # CY-SC001) at least 1 h before experimentation. Phenol red-free medium with SiRActin dye was used throughout the LLSM imaging experiment and was not washed out.

Human lung cancer cells (NCI-H1299 NSCLC, ATCC, Manassas, VA) expressing gd2PAL-Dendra2 (Bassell lab, Emory University) were cultured on glass coverslips in Roswell Park Memorial Institute (RPMI-1640) media supplemented with 10% fetal bovine serum and 100 units/mL of penicillin/streptomycin, and maintained at 37 °C and 5% CO_2_.

### Simulations

To simulate noisy fluorescence images, we have used two different freely available microtubules data sets^15,65^. In both cases, we have generated images based on the parameters: NA = 1.4, wavelength = 700 nm, and pixel size = 100 nm. The final images were generated by corrupting the signal with Poisson noise and then adding sCMOS-related noise as described by Liu et al.^15^.

### Reporting summary

Further information on research design is available in the Nature Research Reporting Summary linked to this article.

## Supplementary information

Supplementary Information

Description of Additional Supplementary Files

Supplementary Movie 1

Supplementary Movie 2

Supplementary Movie 3

Supplementary Movie 4

Supplementary Movie 5

Supplementary Movie 6

Supplementary Movie 7

Supplementary Movie 8

Supplementary Software

Reporting Summary

## Data Availability

The data sets generated and analyzed in this study are available from the corresponding author upon request. Unprocessed example data are included in the Supplementary Software.

## Code availability

The core package for ACsN is available as Supplementary Software. The code has been written in MATLAB (MathWorks) and has been tested with versions 2014b, 2016a, and 2018a. In MATLAB, the software can be run from the command line or using its graphical interface. A plugin to call ACsN directly from ImageJ is also available. To install the package, unzip the compressed folder and follow the instructions in the file readme.txt. For further information about the software, consult the help file (or in MATLAB type: help ACSN). The latest version of the software can be found at: https://github.com/ShuJiaLab/ACsN.
