# Supplementary material for: Fast and accurate sCMOS noise correction for fluorescence microscopy
Source: Nat Commun. 2020 Jan 3;11:94. doi: 10.1038/s41467-019-13841-8 (PMC6941997; doi:10.1038/s41467-019-13841-8)
Supplement: Supplementary file 1 — Supplementary Information [file 41467_2019_13841_MOESM1_ESM.pdf]

Supplementary Information

Fast and Accurate sCMOS Noise Correction for Fluorescence Microscopy

Mandracchia et al.

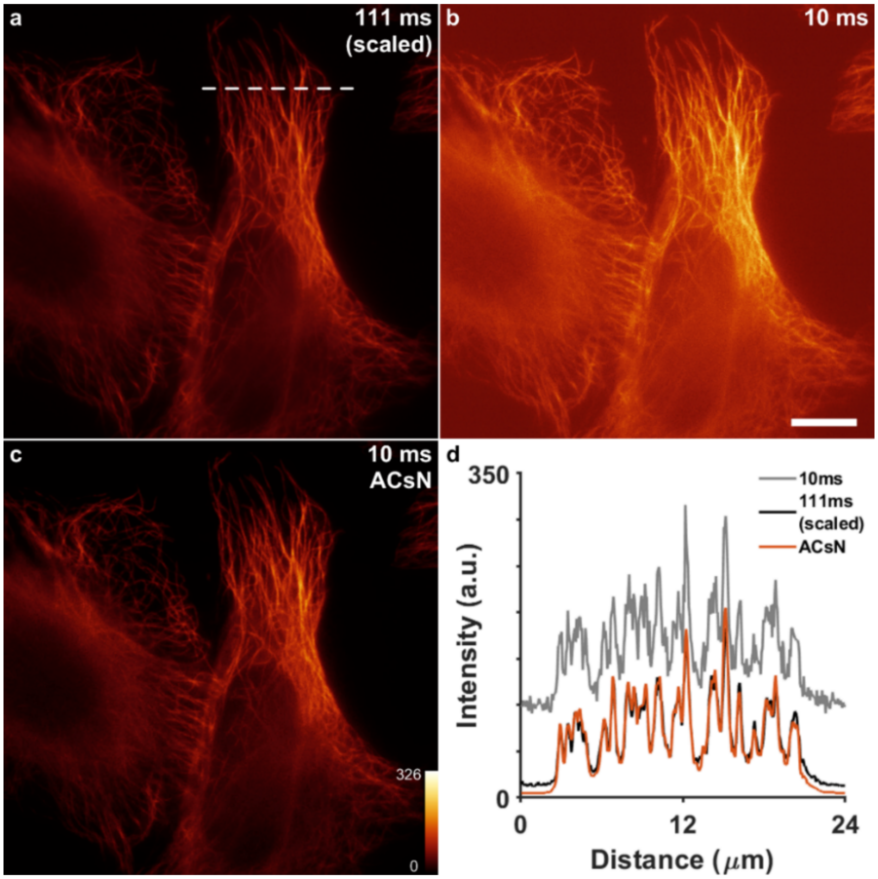

Supplementary Figure 1: ACsN cancels noise while retaining the correct intensity levels. (a) TIRF image of microtubules of fixed HeLa cells acquired with an exposure time of 111 ms. The image intensity has been scaled by 11.1 times for fair comparison with (b) and (c). (b) The same sample with the exposure time set to 10 ms. (c) Denoised image of (b) using ACsN. (d) Comparison of the cross-sectional profiles in (a-c) along the corresponding dashed line in (a). The profile of the ACsN-processed image (c) agrees well with the corresponding signals captured in (a) and shows further denoised background and thus improved SNR. More quantitative results and analysis are shown in Supplementary Table 1. Scale bar: 10 μm

Supplementary Table 1: Improvement of image quality for wide-field microscopy after ACsN denoising.

		Microtubules (Supplementary Figure 1)	Mitochondria (Figure 2a,b)
**SSIM**	Raw	0.47	0.11
	ACsN	0.70	0.92
**PSNR (dB)**	Raw	22.28	10.98
	ACsN	29.19	35.84

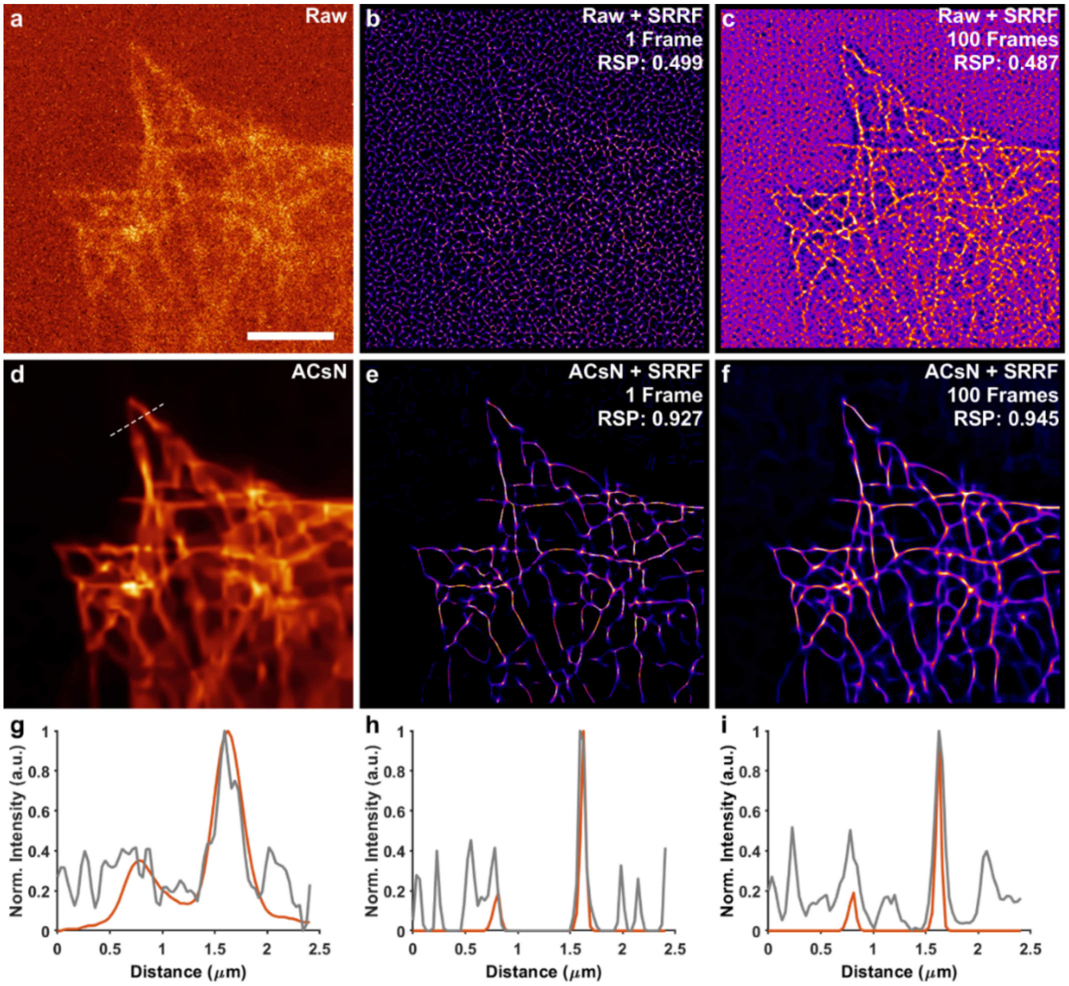

Supplementary Figure 2: ACsN processing improves the performance of SRRF, allowing fast super-resolution imaging. (a) TIRF image of microtubules aquired at a frame rate of 200 Hz (exposure time = 5 ms). (b,c) SRRF deconvolution obtained using 1 and 100 frames, respectively. (d) ACsN reconstruction of (a). (e,f) SRRF deconvolution obtained using 1 and 100 ACsN-processed frames, respectively. (g) Profile of two microtubule filaments in (a, gray) and (d, red) corresponding to the white dashed line in (d). (h-i) The same profiles measured in (b,e) and (c,f), respectively. Scale bar: 4 μm.

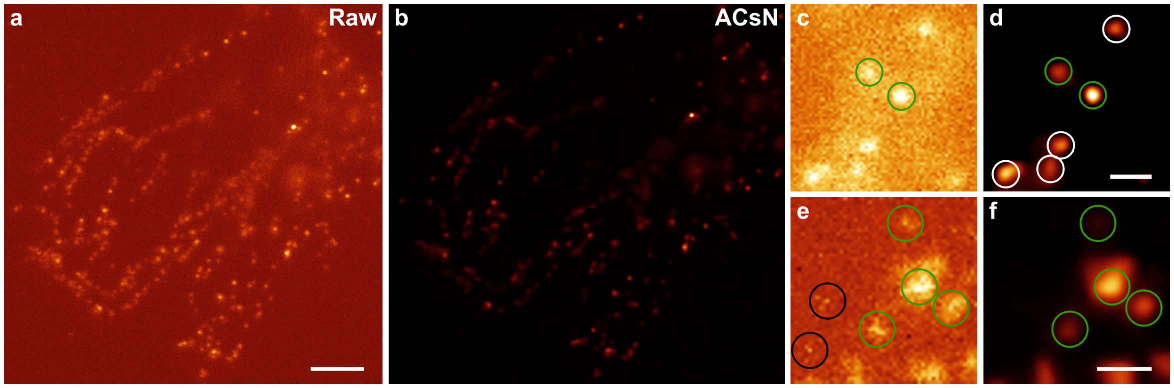

Supplementary Figure 3: ACsN improves localization performance in STORM. (a) A representative single-molecule frame of labeled mitochondria in a HeLa cell. (b) The same frame in (a) after ACsN processing. (c-d) The presence of the readout noise can alter the single molecule signal and produce less localization results (green circles) than expected (white circles). Image (c) was saturated for better visualization of signals covered by the noise. (e-f) Noise and hot pixels can produce patterns that could be misidentified as single molecules (black circles). Scale bars: 4 μm (a), 1 μm (d,f).

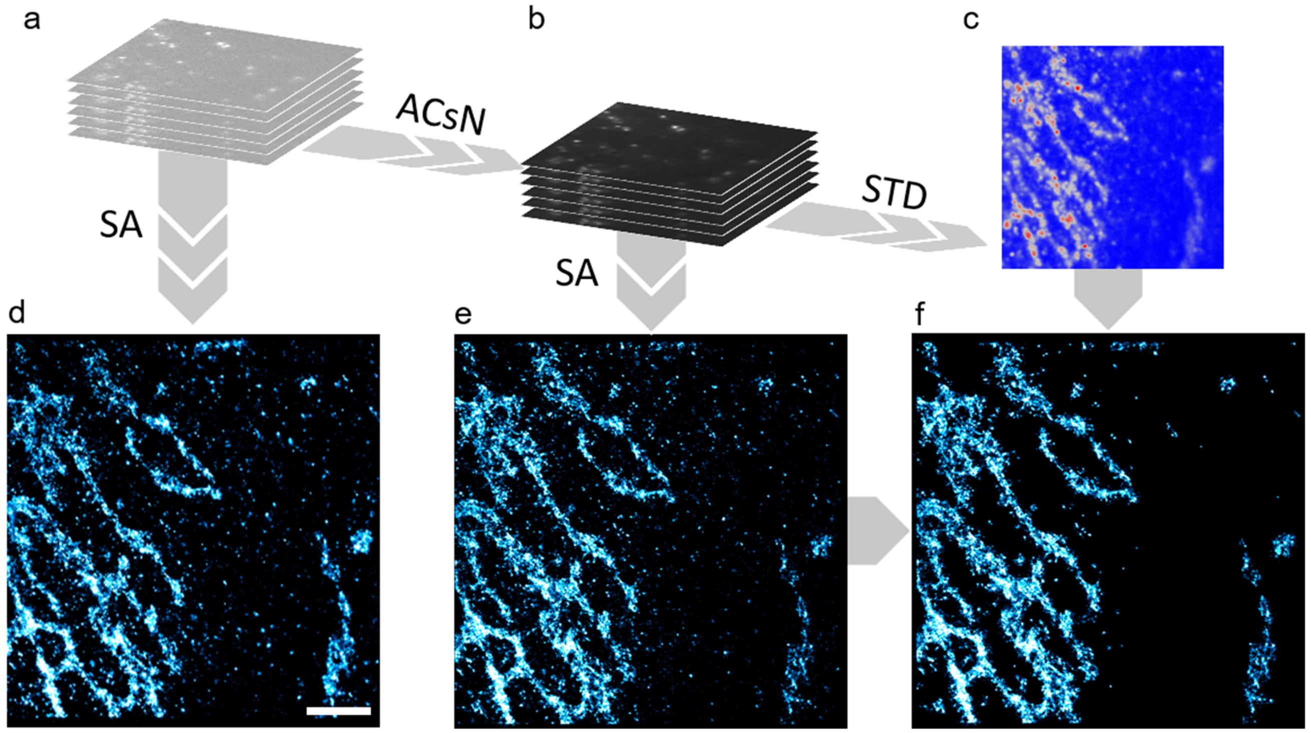

Supplementary Figure 4: The analysis of sample-related fluctuation can minimize the effects of imperfect labeling. A sequence of 30,000 single molecule frames of labeled mitochondria in a HeLa cell was acquired (a) and then processed with ACsN (b). (c) Map of the standard deviation of each pixel in the sequence in (b). After denoising, any pixel fluctuation non-related to the fluorescent molecules is cancelled so that the standard deviation map in (c) is related only to the blinking of fluorescent molecules. (d) The presence of unbound markers can generate a localization noise all over the image that denoising of the raw data cannot fully mitigate (e). However, using the standard deviation image in (c) to map the blinking rate and set a threshold across the field-of-view, it is feasible to suppress the localizations associated with nonspecific markers and thus produce a cleaner STORM image (f). STD: standard deviation of pixel intensity along the image sequence. SA: STORM analysis. Scale bar: 4 μm.

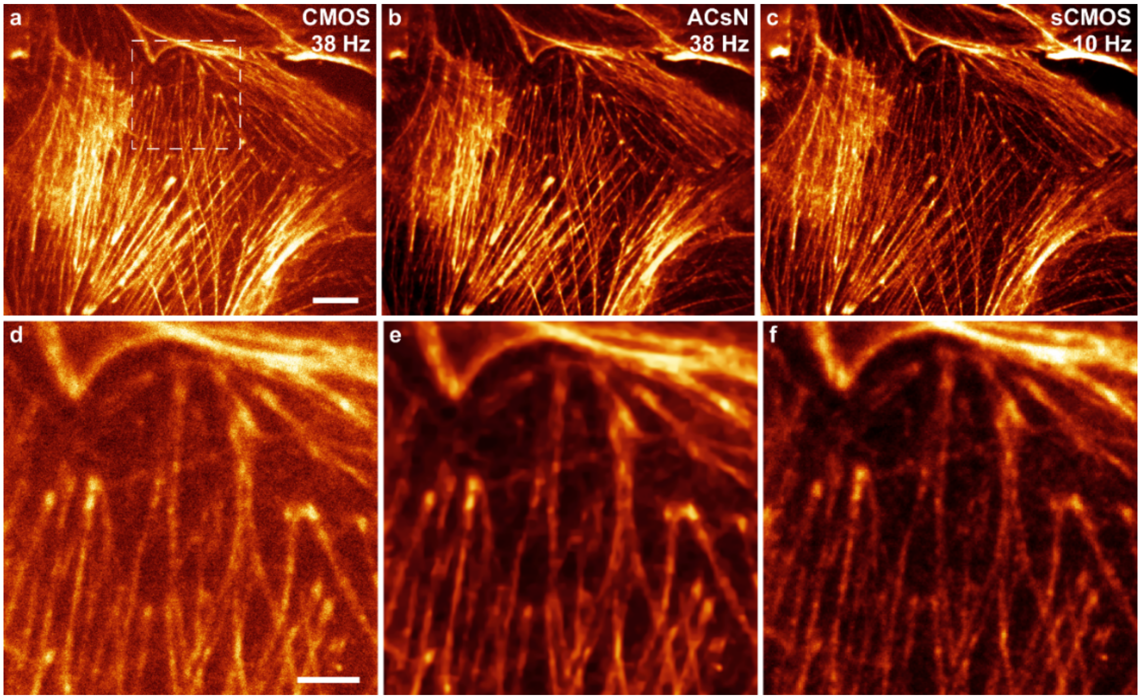

Supplementary Figure 5: TIRF microscopy with an industrial-grade CMOS camera. (a) TIRF image of F-actin in a fixed BPAE cell, taken at a frame rate of 38 Hz. (b) The same image in (a) after ACsN denoising. (c) The same cell in (a) imaged with a sCMOS camera as a reference, taken at a frame rate of 10 Hz. (d-f) Zoomed-in images of (a), (b), and (c), respectively, corresponding to the boxed region in (a). Scale bars: 10 μm (a), 4 μm (d).

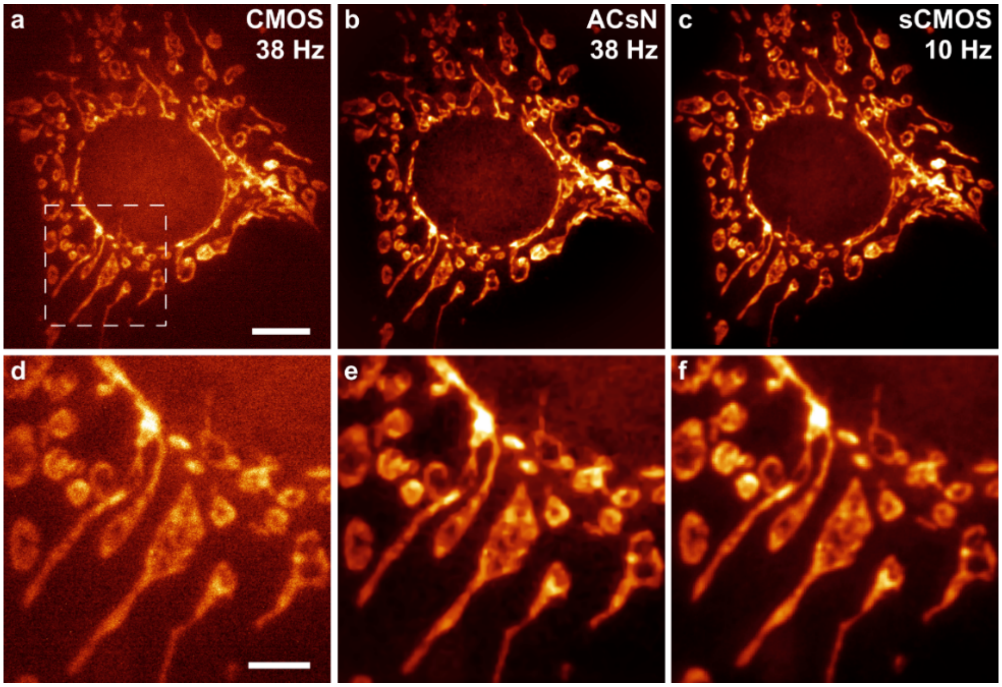

Supplementary Figure 6: Fluorescence microscopy with an industrial-grade CMOS camera. (a) Epi-fluorescence imaging of mitochondria in a fixed bovine pulmonary artery endothelial (BPAE) cell, taken at a frame rate of 38 Hz. (b) The same image in (a) after ACsN denoising. (c) The same cell in (a) imaged with a sCMOS camera as a reference, taken at a frame rate of 10 Hz. (d-f) Zoomed-in images of (a), (b), and (c), respectively, corresponding to the boxed region in (a). Scale bars: 10 μm (a), 4 μm (d).

Supplementary Table 2: Quality improvement of the raw and ACsN denoised images obtained with the industrial-grade CMOS camera with respect to the sCMOS reference.

		**Mitochondria (Supplementary Figure 6)**	**F-actin (Supplementary Figure 5)**
**SSIM**	Raw	0.32	0.15
	ACsN	0.80	0.63
**PSNR (dB)**	Raw	22.39	10.59
	ACsN	30.66	14.50

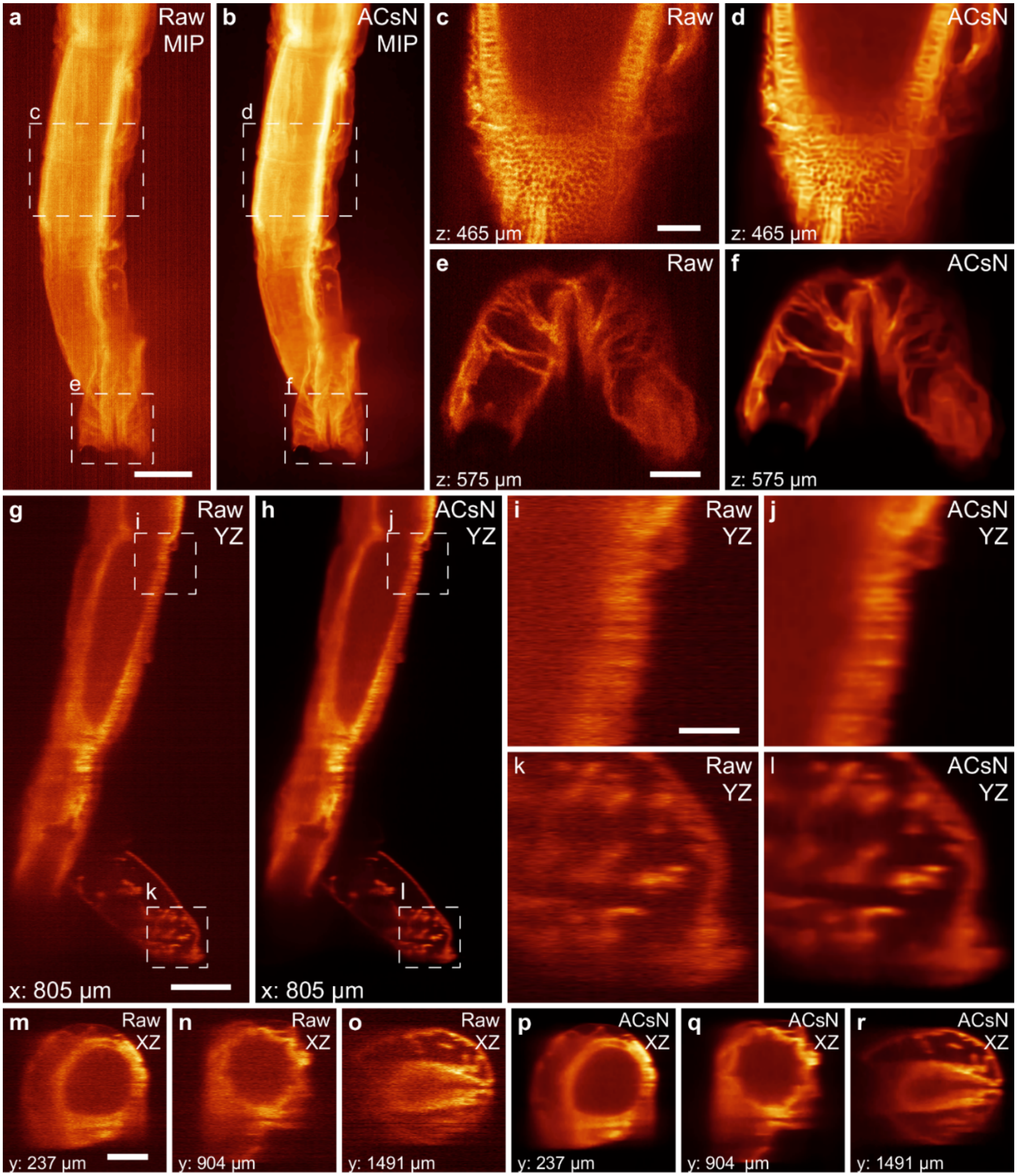

Supplementary Figure 7: ACsN processing of LSFM volumetric data obtained with a SPIM system. (a,b) Maximum intensity projections (MIP) of a fluorescently labeled adult brine shrimp before (a) and after (b) ACsN denoising. (c-f) Zoomed-in images of representative z-slices of the volumetric data corresponding to the positions marked by the dashed boxes in (a) and (b). (g,h) Orthogonal views along the YZ plane at x = 805 μm of both the raw (g) and the denoised (h) volumetric scans. (i-l) Zoomed-in images corresponding to the boxes in (g) and (h). Orthogonal views along the XZ plane of the raw (m-o) and denoised (p-r) volumetric scans at y = 237 μm (m,p), y = 904 μm (n,q), and y = 1491 μm (o,r). Scale bars: 200 μm (a,g), 50 μm (c,e,i), 100 μm (m).

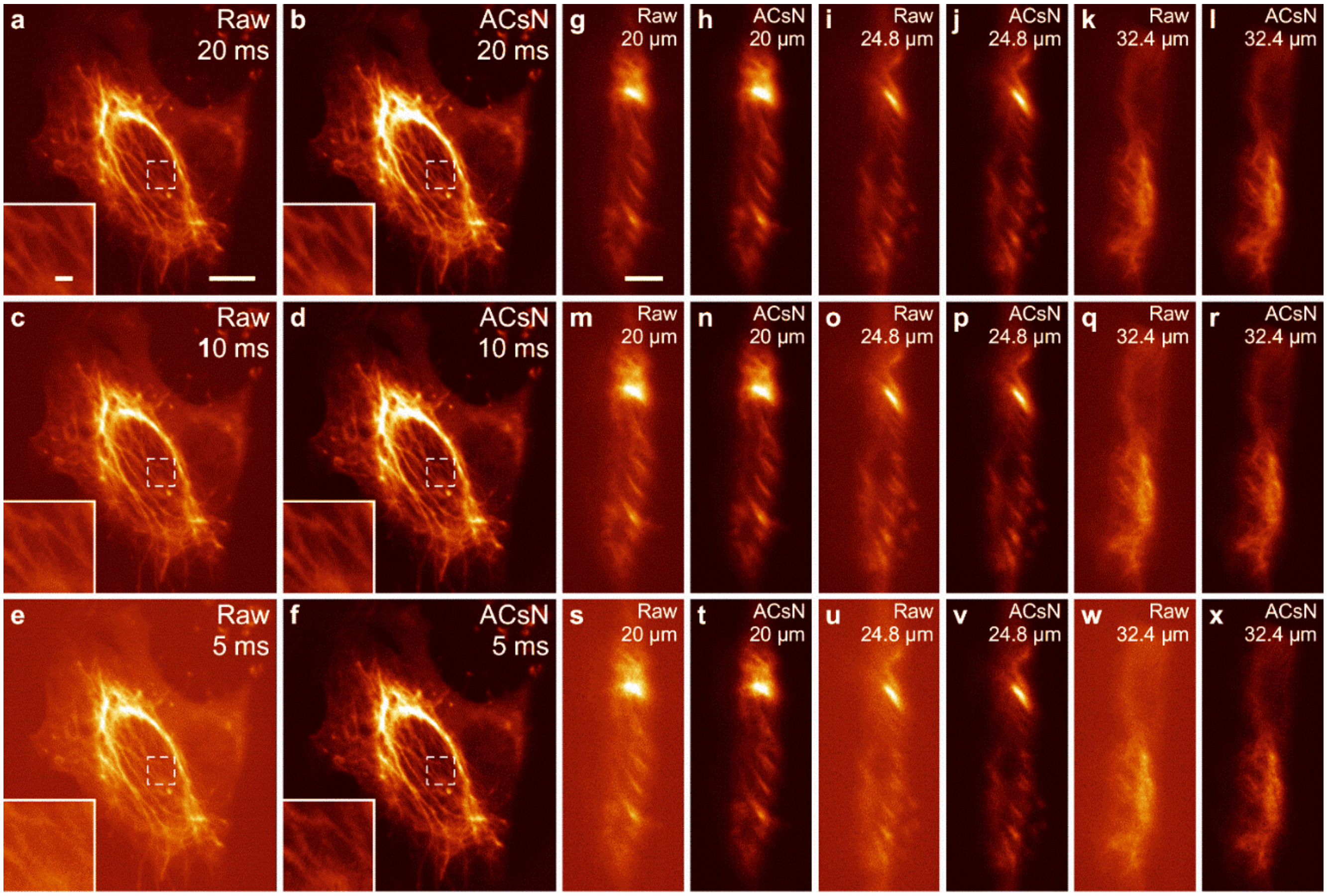

Supplementary Figure 8: ACsN processing of LLSM volumetric imaging of fixed skin cells. (a,c,e) Maximum intensity projections of the raw volumetric data acquired with an exposure time of 20 ms, 10 ms, and 5 ms, respectively. Insets show the zoomed-in images of the corresponding boxed regions. (b,d,f) Maximum intensity projections of the volumetric data in (a), (c), and (e), respectively, after ACsN processing. Insets show the zoomed-in images of the corresponding boxed regions. (g-l) Representative slices of the volumetric data acquired with the 20-ms exposure time before (g,i,k) and after (h,j,l) denoising at position 51 µm (g,h), 63 µm (i,j), and 81 µm (k,l). (m-r) Representative slices of the volumetric data acquired with the 10-ms exposure time before (m,o,q) and after (n,p,r) denoising at position 51 µm (m,n), 63 µm (o,p), and 81 µm (q,r). (s-x) Representative slices of the volumetric data acquired with the 5-ms exposure time before (s,u,w) and after (t,v,x) denoising at position 51 µm (s,t), 63 µm (u,v), and 81 µm (w,x). It should be noted that due to the illumination scheme of LLSM, slice's positions are skewed with respect to the axis normal to the coverslip and do not correspond to the actual z direction. Scale bars: 5 µm (a), 800 nm (a inset), 2 µm (g).

Supplementary Table 3: Improvement of image restoration for the volumetric scans shown in Supplementary Figure 7. The reported number are averages of the SSIM and PSNR values evaluated individually for each light-sheet slice. For the reference image, we used a volumetric scan of the same cell acquired with an exposure time of 20 ms and a laser power of 107 μW.

t_{ex} (ms)	SSIM		PSNR (dB)	
	Raw	ACsN	Raw	ACsN
**5**	0.21 ± 0.03	0.8 ± 0.2	13 ± 3	35 ± 4
**10**	0.38 ± 0.07	0.93 ± 0.07	20 ± 5	40 ± 3
**20**	0.61 ± 0.08	0.97 ± 0.03	26 ± 5	42 ± 3

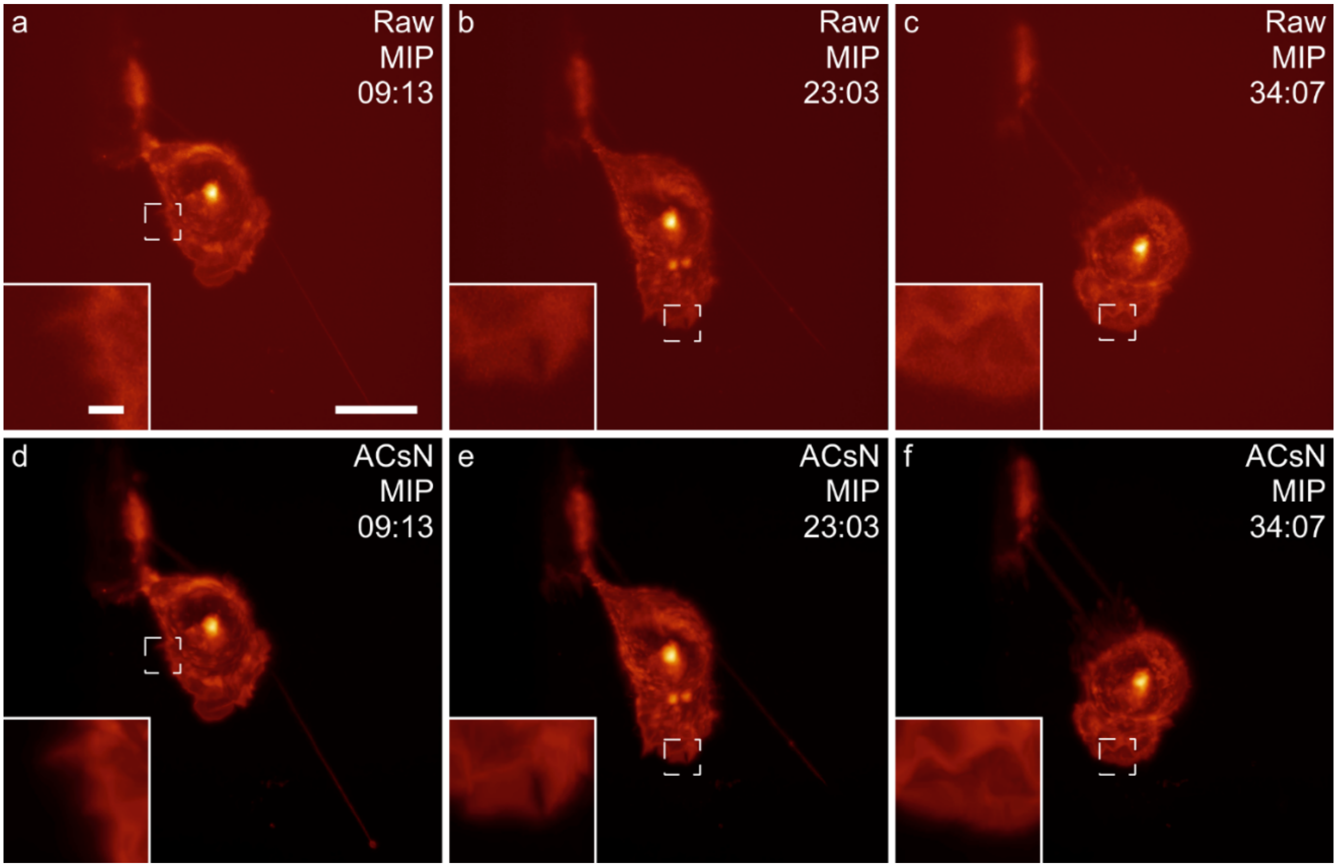

Supplementary Figure 9: ACsN processing of LLSM volumetric scans of a live NCI-H1299 NSCLC cells expressing Dendra2. (a-c) Maximum intensity projection (MIP) of the raw volumetric data at different time points. These volumetric data were acquired in the sample scanning modality. Each scan was composed by 101 slices with a spacing of 561 nm. The exposure time for each slice was 20 ms. (d-f) The same frames depicted in (a-c) obtained by MIP of ACsN-processed data. Here, ACsN utilized the time-lapse stacks of the individual slices for 3D grouping and image deskewing was performed after denoising. The comparison can be better visualized in Supplementary Movies 6 and 7. Scale bars: 20 μm (a), 2 μm (a inset).

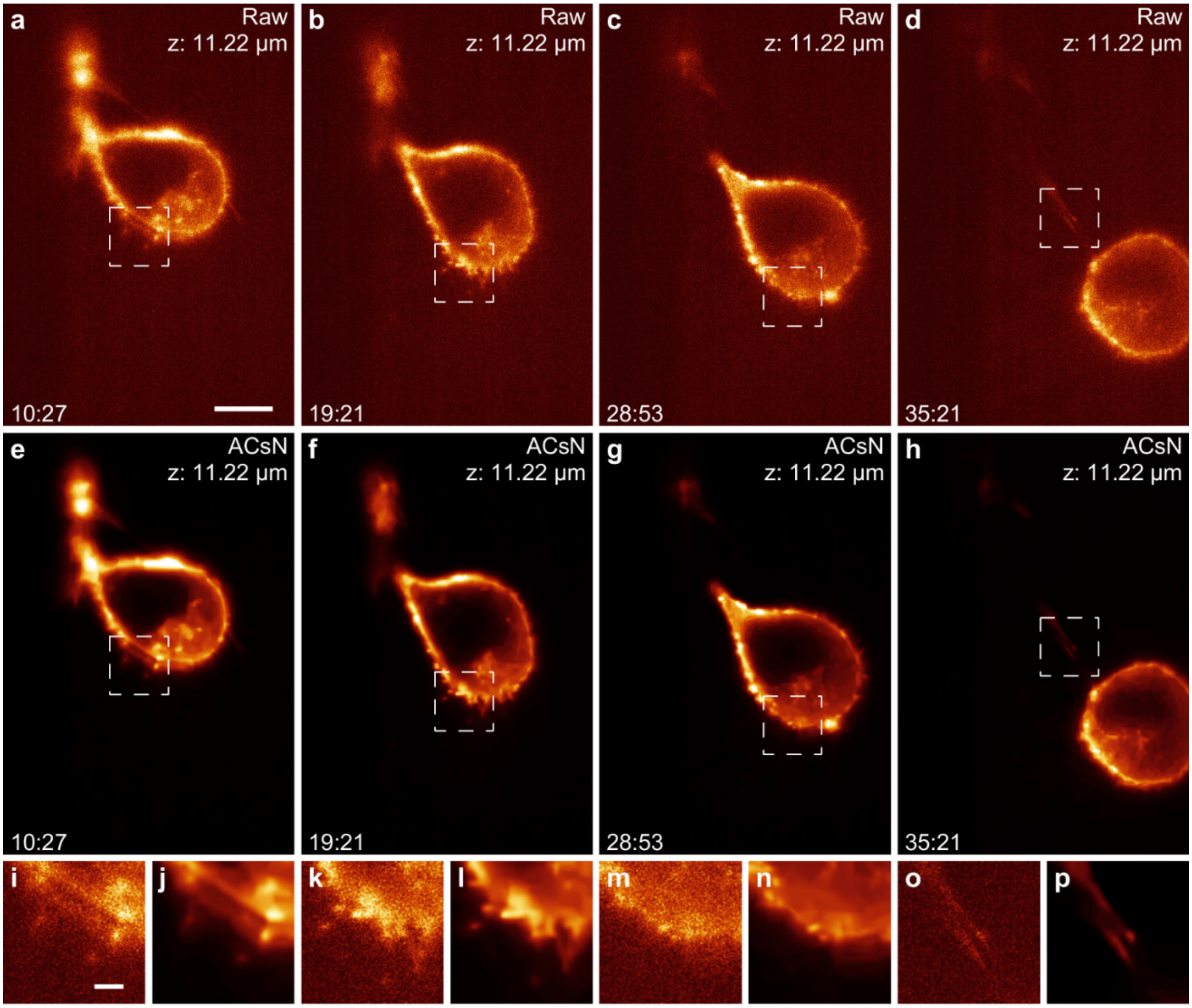

Supplementary Figure 10: ACsN processing of LLSM volumetric scans of a live NCI-H1299 NSCLC cells expressing Dendra2. (a-d) Representative z-slice of the raw volumetric data at different time points acquired with an exposure time of 20 ms. (e-h) The frames depicted in (a-d) after ACsN processing. (i-j) Zoom-ins of the area corresponding to the dashed square in (a) and (e), respectively. (k-l) Zoom-ins of the area corresponding to the dashed square in (b) and (f), respectively. (m-n) Zoom-ins of the area corresponding to the dashed square in (c) and (g), respectively. (o-p) Zoom-ins of the area corresponding to the dashed square in (d) and (h), respectively. Due to the illumination scheme of LLSM, slice's positions are skewed with respect to the axis normal to the coverslip and do not correspond to the actual z direction. These volumetric data were acquired in sample scan mode. Scale bars: 10 µm (a), 2 µm (i).

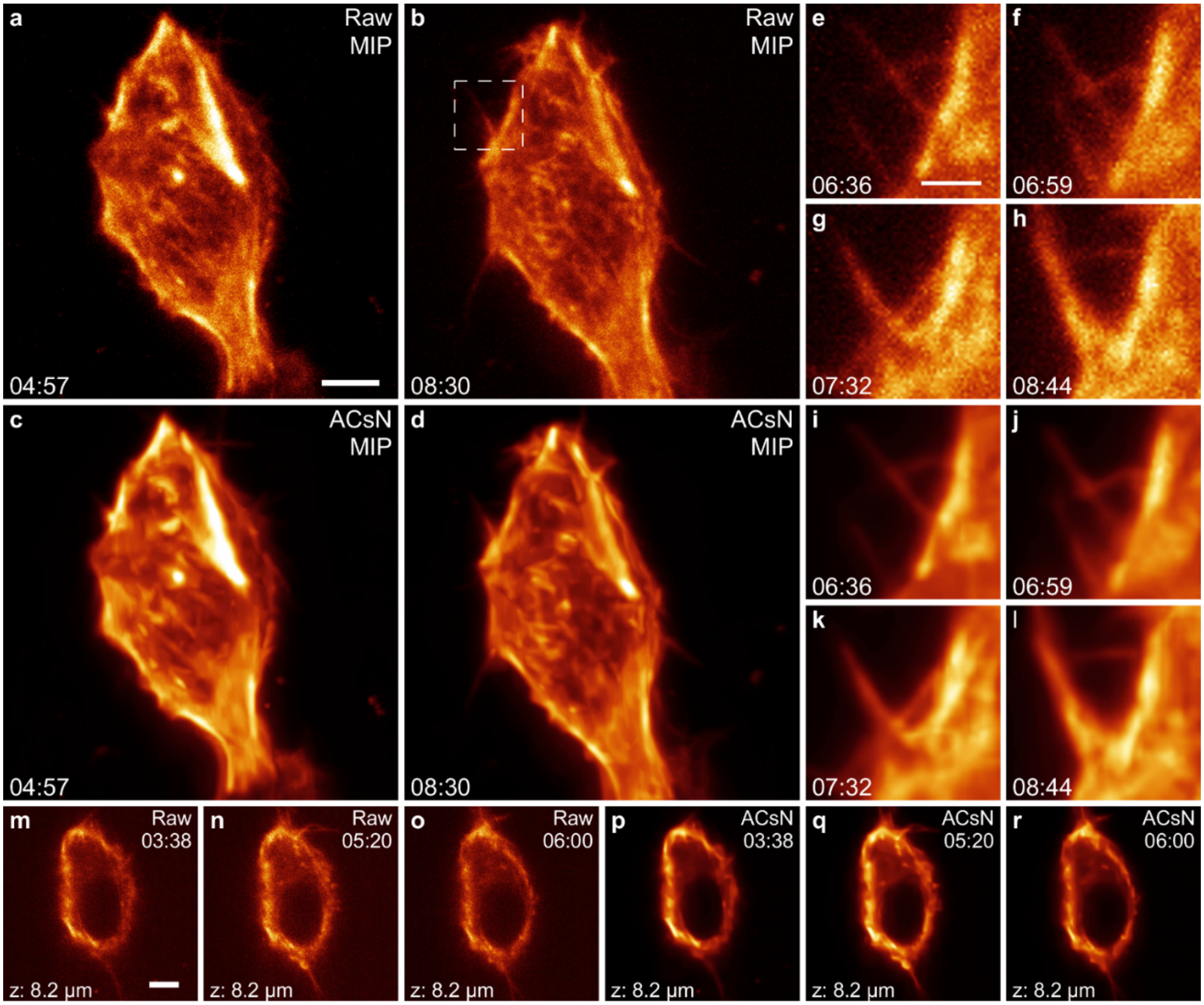

Supplementary Figure 11: ACsN processing of LLSM volumetric scans of a live mouse embryonic fibroblasts (MEFs). (a,b) Maximum intensity projections of the raw volumetric data at two representative time points acquired with an exposure time of 20 ms. (c,d) Maximum intensity projections of the volumetric data in (a) and (b) after ACsN processing of each frame. (e-h) Zoomed-in images of the area corresponding to the dashed square in (b) at different times. (i-l) Zoomed-in images of the same areas in (e-h) after ACsN denoising. (m-o) Representative slice of the volumetric data at different times before (m-o) and after (p-r) denoising. These volumetric data were acquired in the sheet scan mode. The comparison can be better visualized in Supplementary Movie 8. Scale bars: 5 µm (a,m), 2 µm (e).

Supplementary Table 4: List of the sCMOS and CMOS sensors used in this work with the corresponding applications reported in the manuscript.

Sensor model	Sensor Type	Readout noise	QE (max)	Applications
**Hamamatsu ORCA Flash v.3**	sCMOS	$0.8_{med}/1.4_{rms}$ e^- @ 30 Hz<br>$1.0_{med}/1.6_{rms}$ e^- @ 100 Hz	82%	Epi-fluorescence, TIRF, Light-field microscopy, Single particle tracking, STORM, Deconvolution
**Hamamatsu ORCA Flash v.2**	sCMOS	$0.8_{med}/1.4_{rms}$ e^- @ 30 Hz<br>$1.0_{med}/1.6_{rms}$ e^- @ 100 Hz	82%	Lattice light-sheet microscopy
**PCO.Edge**	sCMOS	$1.1_{med}/1.5_{rms}$ e^- @ 33 Hz<br>$1.5_{med}/1.7_{rms}$ e^- @ 100 Hz	60%	SPIM
**FLIR Imaging, GS3-U3-51S5M-C**	CMOS	1.6_{med} e^- [ref.1]	76%	Epi-fluorescence and TIRF microscopy
**ON Semiconductor, MT9V032C12STM**	CMOS	10_{rms} e^- [ref.2]	60%	Miniaturized microscopy

Supplementary Notes

Table of Contents

Supplementary Note 1. Physical principles behind sCMOS noise **16**

Supplementary Note 2. Automatic correction of sCMOS-related noise **18**

2.1 *Pseudo code* *18*

2.2 *Canceling fixed-pattern noise sources* *20*

2.3 *Noise estimation* *21*

2.4 *Sparse filtering* *23*

2.5 *Denoising of video sequences* *24*

Supplementary Note 3. Algorithm characterization **27**

3.1 *Parameters optimization* *27*

3.2 *Resolution* *28*

3.3 *Reduction of pixel fluctuation* *31*

3.4 *Dependency on the incoming photon flux* *33*

3.5 *Sampling rate* *37*

3.6 *Self-similarity of the input image* *40*

Supplementary Note 4. Additional application notes **43**

4.1 *Deconvolution microscopy* *43*

4.2 *Single-particle tracking* *48*

Supplementary Note 5. Comparison with the state-of-the-art **52**

Supplementary Note 6. Runtime **58**

Supplementary Note 7. No-reference assessment of image quality **59**

Supplementary Note 8. Discussion & Outlook **61**

References **65**

Supplementary Note 1. Physical principles behind sCMOS noise

Noise in a camera image is the aggregate spatial and temporal variations in the measured signal, assuming constant, uniform illumination. The total effective noise per pixel is the quadrature sum of different noise components. The most relevant ones are:

Dark Shot Noise (D_S). Dark current is a current that flows even when no photons are incident on the camera. It is a thermal phenomenon resulting from electrons spontaneously generated within the silicon chip (valence electrons are thermally excited into the conduction band). The number of dark electrons collected during the exposure is the dark shot noise, which does not depend on the signal level.

Photon Shot Noise (P_S). This is the statistical noise associated with the arrival of photons at the pixel. Since photon measurement obeys Poisson statistics, the photon shot noise is given by the standard deviation of Poisson distribution, σ_P, which is equal to the square root of the signal.

Readout Noise (R). This is the noise generated in producing the electronic signal, which results from the sensor design but also by the design of the camera electronics. CMOS sensors are silicon-based integrated circuits formed by a dense matrix of photo-diodes that convert photons into electronic charge[3]. This charge, generated by the interaction between photons and silicon atoms, is stored in a potential well. CMOS pixels perform charge-to-voltage conversion at each location using additional circuitry that increases readout noise and generates extra fixed-pattern noise sources compared to CCDs[4].

Fixed-pattern Noise. Given a uniform light source, different CMOS pixels will generate a different number of photoelectrons from the same number of impinging photons. This difference in pixel response is referred as photo-response non-uniformity or fixed-pattern noise, and is caused by variations in pixel geometry, substrate material and micro-lenses[5]. This is even more relevant in sCMOS sensors, where the increased signal capacity and much lower readout noise comes at the expenses of the fixed pattern noise due to pixel gain fluctuation[6].

The effect of fixed-pattern noise depends on the electronics of the camera, is proportional to the illumination level, and can be modeled by a multiplicative component, γ, and a bias, β.

Ideal camera. We consider an ideal camera as a device that achieves the maximum pixel sensitivity. This means that every photon is converted to a photoelectron, i.e. the quantum efficiency (QE) is 100%, every pixel voltage is digitized identically, i.e. $\gamma = 1$ and $\beta = R = 0$, and there is no dark current, i.e. $D_S = 0$.

The relation between the sensitivity and the quality of an image is given by the signal to noise ratio (SNR) as a function of the input photon number (S). The formula to calculate the SNR for a single pixel at various light intensities is:

$$\mathrm{SNR} = \frac{QE\cdot(\gamma\cdot S+\beta)}{\sqrt{QE\cdot(\gamma\cdot S+\beta+b)+(R)^2}} \quad (1)$$

where b is the signal background.

In the case of a perfect detector with an ideal signal, i.e. no background, this equation becomes:

$$\mathrm{SNR} = \sqrt{S} = \sigma_{\mathrm{P}}, \quad (2)$$

As mentioned above, $P_S = \sigma_{\mathrm{P}}$, which reminds us that even with an ideal camera, the signal is still affected by photon shot noise, which is intrinsic of photon statistics. However, even if noise cannot be completely eliminated from the measuring process, effort must be made in order to minimize the noise sources of the devices.

Here we present a software for the Automatic Correction of sCMOS-related Noise (ACsN) that combines camera calibration, noise estimation and sparse filtering in order to correct the most relevant noise sources generated by a sCMOS camera.

Supplementary Note 2. Automatic correction of sCMOS-related noise

2.1 Pseudo code

A pseudo-code of the algorithm is presented in Supplementary Table 5 and illustrated in Supplementary Figure 12. The algorithm uses the known parameters of the experimental setup to correct pixel-dependent signal fluctuation and to produce a precise estimation of the readout noise. Then, sparse filtering is used to remove the predicted noise through the enhancement of the self-similarity of the sample. The details of the architecture of the ACsN algorithm are described in the Supplementary Notes 2.2-2.5.

This results in a quantitative image restoration that permits to retrieve from noisy data the same information that would be otherwise obtained with a higher-intensity, slower imaging.

Supplementary Table 5: Pseudo-code of the ACsN algorithm.

LOAD
Load image
Load acquisition parameters: NA, lambda, pixel size
Load camera parameters: gain map and offset value
FIXED PATTERN NOISE REMOVAL
Remove offset
Adjust pixel value with camera gain map
NOISE ESTIMATION
Calculate OTF cutoff frequency
Produce high-pass filtered image
Estimate noise variation
SPARSE FILTERING
Perform grouping on input image
Hard thresholding
Perform grouping on thresholded image
Wiener filtering
Image quality check
↳ 3D Grouping and Video Processing

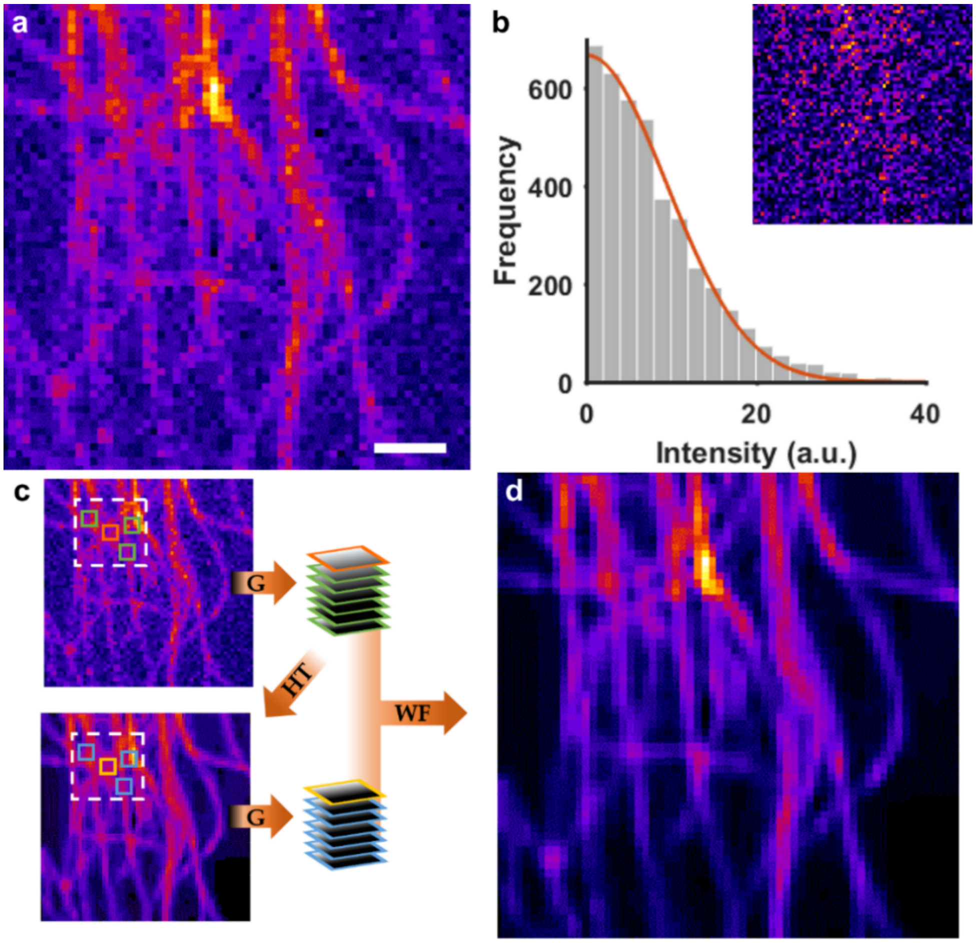

Supplementary Figure 12: Main steps of the ACsN algorithm. (a) The noisy input image is rescaled using offset and gain pixel maps. (b) The variation of the noise in the input image is estimated by the intensity distribution of the pixel values in a high-pass filtered image (inset). This image is generated setting the threshold of the filter at the OTF boundary. This way all the signal is filtered out of the image. The estimated noise variation is used as a parameter for sparse denoising in (c). (c) The input image is scanned in steps of 3 pixels. At each step, a reference patch is chosen (orange square) and the software selects up to 8 similar patches (green squares) within a certain neighborhood (dashed square). All these patches are then grouped (G) in 3D stacks. Each stack is filtered using 3D hard thresholding (HT) and aggregated to generate an intermediate image. Grouping (G) is performed again on the intermediate image to obtain new 3D stacks. Finally, Wiener filtering (WF) is applied to both sets of stacks to generate the final image (d). Scale bar: 10 pixels.

2.2 Canceling fixed-pattern noise sources

The number of photons $S_p(\tau)$ impinging the photo-diode p during a certain exposure time τ follows a Poisson distribution. If we suppose that an electron is generated for each absorbed photon, the number of electrons generated in the potential well is also Poisson distributed. In the ideal case, the voltage measured at the sensor output should be proportional to the collected charge: $V = Cq = C \cdot S_p(\tau)$, where C is the equivalent capacitance of the photo-diode and q the electric charge.

However, the presence of fixed-pattern noise in sCMOS cameras generates in different pixels a different number of photoelectrons from the same number of impinging photons. This effect is proportional to the illumination level and reflects the probability that a photon can be absorbed by the photo-diode. It is modeled as a multiplicative factor γ_p applied to the parameter of the Poisson distributed variable $S_p(\tau)$.

During the AD conversion, the voltage produced by each pixel is read as a difference from a reference level, which represents the absence of light. In practice, this reference voltage is assigned a positive value large enough that voltages below the reference will still correspond to positive values. This is responsible for a bias (β_p) in the measured intensity values, especially relevant at low light levels.

Finally, another kind of fixed-pattern noise is related to the fact that the readout for CMOS sensors is performed line by line. At a given time, all columns of one line are readout through the output column amplifiers. Differences from one column amplifier to another introduce a column fixed pattern, which is also proportional to the signal intensity and its contribution can be included in γ_p and β_p.

The acquisition of a sCMOS camera can be modeled, then, by the equation[7,8]:

$$Z_p = \gamma_p \text{Pois}\{S_p(\tau)\} + N(0, \sigma_R) + \beta_p, \quad (3)$$

where Z_p is the value of the pixel p and $N(\mu_R, \sigma_R)$ the Gaussian distributed readout noise of mean $\mu_R = 0$ and standard deviation σ_R. In this simplified model we have omitted the contribution of dark current, which can be neglected for exposure times below 1s, and the quantization noise due to the analog to digital conversion, which is negligible compared to readout noise[4,9].

Since fixed-pattern noise depends only on the camera circuitry, β_p and γ_p can be estimated through a one-time calibration. We have mapped the pixel-dependent offset β_p by averaging a series of dark frames while the gain γ_p was estimated from multiple sets recorded at different illumination intensities (see Methods).

Once both β_p and γ_p are available, the acquired image can be rescaled to remove the fixed-pattern noise component from the measurement:

$$M_p = \frac{Z_p - \beta_p}{\gamma_p}. \quad (4)$$

Clearly, M_p represents a mixture of the incoming photons from the sample and the randomly generated readout noise from the AD conversion. Thus, in order to obtain S_p, a careful

estimation of both the readout noise $N(0, \sigma_R)$ and the fluctuation due to photon shot noise $\text{Pois}\{S_p(\tau)\}$ is necessary in order to remove the noise efficiently while preserving the underlying signal.

2.3 Noise estimation

In the previous section, we modeled camera-related noise as the contribution of both readout noise and photon shot noise. It is important to notice that these two contributions have different statistics. In fact, the former is a Gaussian white noise, while the latter follows a Poisson distribution. However, the Poisson distribution becomes a good approximation of the Gaussian distribution when the photon flux (λ_P) is greater than 3 photons per pixel[10] (see Supplementary Figure 13a) and the error committed by approximating σ_P with a Gaussian standard deviation $\sigma_G$is less than 1% when the photon flux is more than 5 photons per pixel (see Supplementary Figure 13b).

Notably, the abovementioned conditions on the photon flux are usually satisfied for many applications in fluorescence microscopy. Therefore, we consider the camera-related noise as the result of the sum of two independent Gaussian-distributed random variables, which, by theory, is still a Gaussian-distributed random variable:

$$N(\mu_N, \sigma_N) = N(0, \sigma_R) + N(\mu_P, \sigma_G) = N\left(\mu_P, \sqrt{\sigma_R^2 + \sigma_G^2}\right). \quad (5)$$

Thus, we model the camera noise as a white Gaussian noise, which means that it generates a pattern of Gaussian distributed intensities in each frame and has a constant power spectral density (PSD). In particular, the constant PSD implies that the noise is present at every frequency and cannot be simply removed by band-pass filters in the Fourier space.

An alternative solution is to adopt patch-based denoising techniques, which rely on the self-similarity of the sample in space or time[11]. Here, in order to untangle the contribution of the actual signal from the camera noise in M_p, we evaluate the self-similarity based on the sparsity in the Fourier space.

However, an accurate estimation of the noise variation, σ_N, is fundamental to retain the fidelity of the reconstruction, both in terms of quantitativity of the recovered intensity values and resolution of the image. To this end, we use a few known instrumental parameters, i.e. numerical aperture (NA), emission wavelength and pixel size, to calculate the radius of the optical transfer function (OTF) of the system. Given that most of the signal will be contained by the OTF[12,13], we use a high-pass filter to isolate the high frequencies of the image, where the noise contribution is separated by the statistics of the photoelectrons. This way, it is feasible to characterize the noise and obtain a precise estimation of σ_N.

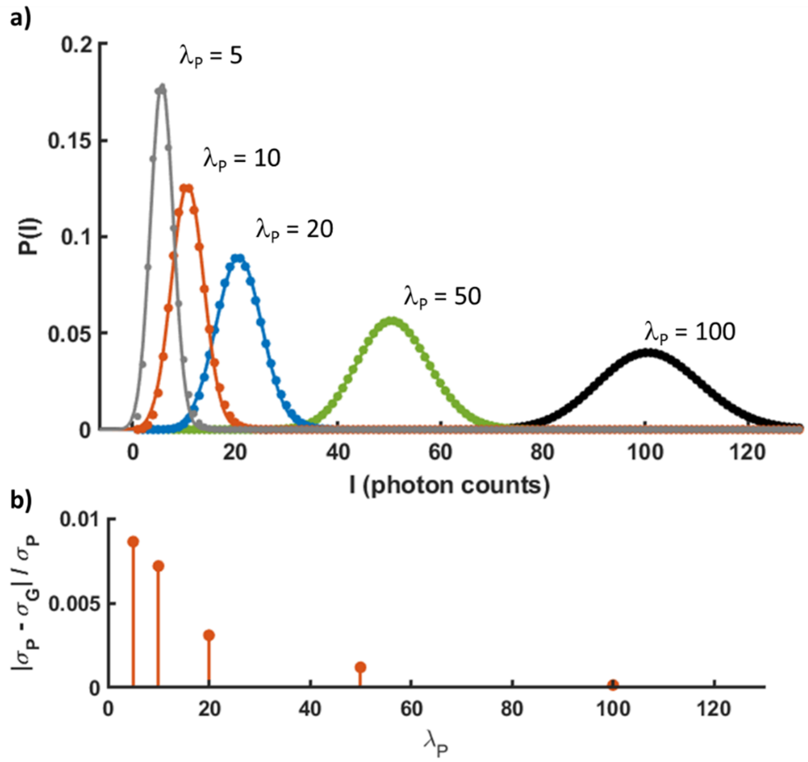

Supplementary Figure 13: Gaussian approximation of a Poisson distribution. (a) Gaussian fitting (solid lines) of Poisson-distributed data (dots) for different values of photon flux λ_P. In all cases $\chi^2 > 0.99$. (b) The relative error between the Poisson and Gaussian standard deviations for $\lambda_P \geq 5$ is less than 1%.

If we call *H(f)* the modulation transfer function (MTF) of the system, we have that:

$$\sigma_N^2 = \int_0^\infty N_0 |H(f)|^2 \mathrm{d}f, \tag{6}$$

where N_0 is a constant value that represents the noise power per unit bandwidth.

In practice, white noise cannot be fully recorded because every camera acts as a low pass filter with cutoff frequency $\bar{f}$ and, thereby, its MTF will be:

$$H(f) = \begin{cases} 1, & f \leq \bar{f} \\ 0, & f > \bar{f} \end{cases} \tag{7}$$

and the exact variance will be:

$$\sigma_N^2 = \int_0^{\bar{f}} N_0 |H(f)|^2 \mathrm{d}f. \tag{8}$$

Now, if we apply a high pass filter with cutoff frequency f_c equal to that of the optical system to the input noisy image, we will obtain another image with:

$$\sigma_c^2 = \int_{f_c}^{\bar{f}} N_0 |H(f)|^2 \mathrm{d}f. \tag{9}$$

From Supplementary Equations (7) and (8) we can obtain:

$$\frac{\sigma_N^2}{\sigma_c^2} = \frac{\int_0^{\bar{f}} N_0 |H(f)|^2 \mathrm{d}f}{\int_{fc}^{\bar{f}} N_0 |H(f)|^2 \mathrm{d}f} \tag{10}$$

Finally, substituting Supplementary Equation (6) in (9) and solving the integrals, we have that:

$$\sigma_N = \sqrt{\frac{\bar{f}}{(\bar{f}-f_C)}}\sigma_C. \tag{11}$$

Aberrations in the microscope system might alter the effective OTF radius, which makes the use of the theoretical value a conservative approach. However, this does not affect the estimation of σ_N as long as the PSD of the noise remains constant with the frequency, as mentioned earlier in this section.

2.4 Sparse filtering

Once the standard deviation of the noise is known, we proceed to filter it out. To do so, we use sparse filtering to take advantage of the self-similarity of the sample[14]. The process can be divided into three main parts: grouping, collaborative filtering and aggregation.

The grouping uses the block-matching concept to find similar patches for each reference patch. It groups a reference patch with the similar patches into a 3D array[15,16]. Specifically, the algorithm chooses a $k \times k$ patch (P) in the noisy image and searches an $m \times m$ area for other similar patches (Q). Such similarity is assessed by the normalized square distance in L_2:

$$d(P,Q) = \frac{\|P-Q\|_2^2}{k^2} \tag{12}$$

Then, the similar patches are stacked in a 3D group, $\mathcal{P}[P]$, up to a maximum of 8. This is repeated with different reference patches until all the noisy images are stacked in 3D groups.

At this point, collaborative filtering employs a 3D transform, τ_{3D}^h, to filter the coefficients of each group all at once. Due to the similarity between the patches, the 3D transform results in even sparser representation of the original patches than the 2D transforms whereas the noise still has a constant power spectrum[17]. Collaborative filtering can be expressed by the formula:

$$\mathcal{P}^h(P) = {\tau_{3D}^h}^{-1}\left(\gamma\left(\tau_{3D}^h(\mathcal{P}[P])\right)\right), \tag{13}$$

where γ is a hard-thresholding operator with threshold $\lambda_\gamma \sigma_N$:

$$\gamma(x) = \begin{cases} 0 & |x| \leq \lambda_\gamma \sigma_N \\ x & \text{otherwise} \end{cases} \tag{14}$$

where σ_N is the previously determined noise variation (see Supplementary Note 2.3), and λ_γ a multiplicative factor.

Once the collaborative filtering produces all the patch estimates, the aggregation procedure returns the denoised patches to their original locations. During the grouping part, one patch can be assigned to more than one group. This implies that pixels can have more than one estimation. The final estimate is computed as a weighted average of all overlapping pixels. The

weights are defined so that homogeneous patches are prioritized over the patches containing edges and corners:

$$w_P = \begin{cases} (N_P)^{-1} & \text{if } N_P \geq 1 \\ 1 & \text{otherwise} \end{cases} \tag{15}$$

where N_P is the number of non-zero coefficients in the 3D block after hard-thresholding.

Finally, the image obtained after this process is used as an intermediate denoised image for a second run of sparse filtering. This second-time process is the same as the first one with the only difference being the use of Wiener filtering on both noisy and intermediate images instead of hard-thresholding:

$$\mathcal{P}^w(P) = {\tau_{3D}^{w}}^{-1}(c_P \cdot \tau_{3D}^{w}(\mathcal{P}[P])), \tag{16}$$

where the Wiener coefficient is:

$$c_P = \frac{\left|\tau_{3D}^{w}(\mathcal{P}[P])\right|^2}{\left|\tau_{3D}^{w}(\mathcal{P}[P])\right|^2 + \sigma_N^2}. \tag{17}$$

It is important to note how both collaborative filtering steps depend on the estimation of σ_N and how, thus, the accuracy of this estimation affects the effectiveness of the denoising process. In particular, the threshold $\lambda_\gamma \sigma_N$ is one of the most important parameters in terms of denoising performance[18].

2.5 Denoising of video sequences

In case of noisy video sequences, ACsN evaluates the image quality improvement and has the option to perform a further sparse filtering step, referred to as sparse video filtering[19,20]. This is similar to the first sparse filtering operation with the difference being that during the grouping task the algorithm looks for similar patches also in the neighboring frames. This means that the search area for similar patches this time is not an $m \times m$ square but an $m \times m \times t$ spatio-temporal volume. This way, lingering noise can be further reduced taking advantage of the sample self-similarity, not only in space, but also in time.

The spatiotemporal correction is performed first along the time direction and then across the space. The similar patches are searched in a three-dimensional sequence of blocks built following a specific trajectory, which is supposed to follow the motion in the scene. Indeed, to maximize the temporal correlation, during video filtering the search window is shifted according to a motion estimation of the reference patch in time. This is performed using a motion estimator with the sequence down-sampled by a factor of two and computing the motion trajectories using a fast diamond search[21]. The trajectory of a block is defined by the sequence of the most similar blocks in the neighboring t frames. Note that down-sampling increases the signal-to-noise ratio, making the motion estimation less impaired by noise[20].

It is important to notice that the motion estimation is performed over an area of $q \times q$ pixels for each frame, where q is chosen in order to alleviate the sampling requirements in relation to the dynamics while retaining a low runtime by parallelizing the processing of each area.

Nonetheless, it is also important to notice that ACsN achieves its maximum efficiency if the number of patches in each group is maximized. This implies that the optimal temporal sampling should assure the presence of a moving feature within the each area during at least 4-8 frames.

The additional denoising operation roughly doubles the processing time (see Supplementary Note 6), so it is important that it is run only when needed. Moreover, the improvement of video filtering becomes relevant only for very noisy images. For this reason, after the first sparse filtering step is performed, the improvement is evaluated by measuring the peak signal-to-noise ratio (PSNR) of each frame. In this evaluation, the average between each frame and its 5 nearest neighbors is used as reference image. If the PSNR, on average, is lower than an empirical threshold, the video filtering is performed. We have found that this is most likely to happen when the value of PSNR falls below 35, which proved to be an optimal threshold in order to reach a satisfying compromise between speed and image quality (see Supplementary Figure 14).

Finally, it should be noted that video filtering involves only an extension of the search window during the grouping phase and does not involve any time averaging, so that the temporal resolution is preserved.

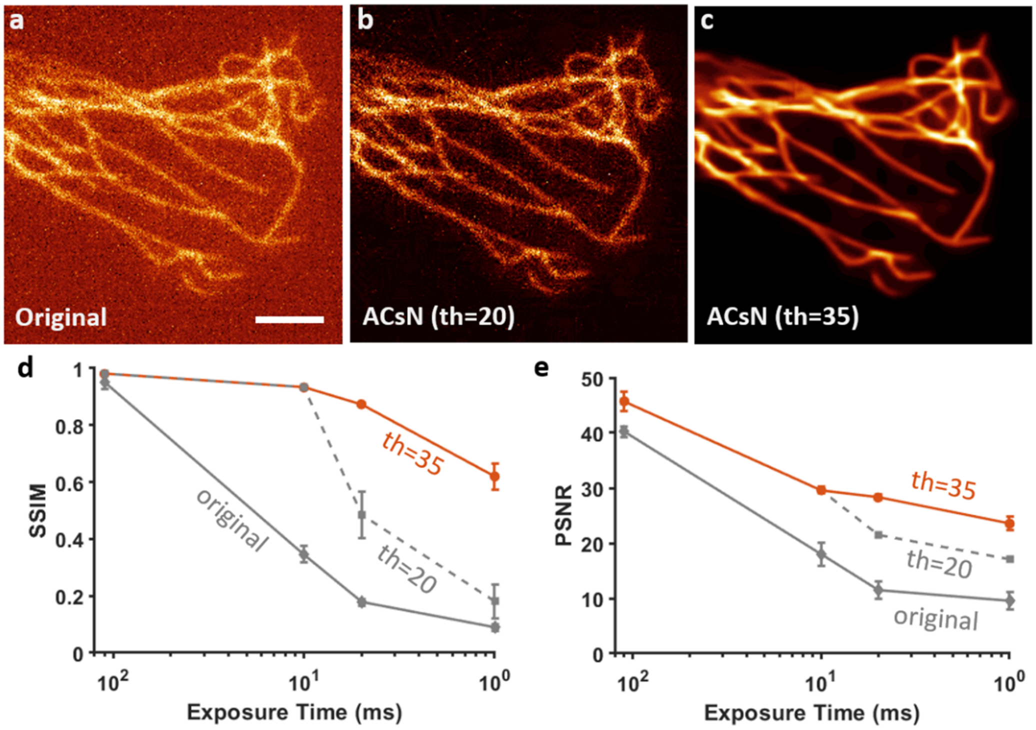

Supplementary Figure 14: Video processing improves the quality of image restoration. (a) Image of microtubules in a HeLa cell. This is a representative frame of a sequence of 100 frames recorded with an exposure time of 5 ms. (b,c) Denoised images of the frame in (a) obtained setting the PSNR threshold for video denoising to 20 (b) and 35 (c). (d,e) Both structural symmetry index measurement (SSIM) and peak signal-to-noise ratio (PSNR) show a significant improvement at exposure times lower than 10 ms. Each data point represents the average SSIM and PSNR calculated over four series of 100 microtubule images of HeLa cells and the error bars represent the respective standard deviation. Each dataset was acquired with different exposure times, namely: 111 ms, 10 ms, 5 ms and 1 ms. Each series was processed with ACsN using different thresholds for video processing. For more details about the calculation and meaning of SSIM and PSNR, please refer to the Methods section in the main text.

Supplementary Note 3. Algorithm characterization

3.1 Parameters optimization

The performance of ACsN was tested on both simulated and experimental data. The input parameters that are given to the program are related to both the camera and optical systems. In the former case, camera gain and offset matrices are needed. These matrices are pre-determined experimentally for each camera (see Methods in the main text). In the latter case, the input parameters are the nominal values for the numerical aperture, wavelength and pixel size in the image plane (i.e. physical pixel size divided by magnification).

The other parameters used in the program are fixed and have been chosen in order to maximize both accuracy and speed. The width and height of the patches, are fixed to $k = 8$ pixels, while the width and height of the neighborhood of the reference patch, in which other similar patches are searched, are set to $m = 19$ pixels. According to Maggioni et al.[20], for video denoising we set the number of neighboring frames of the spatio-temporal search volume to $t = 9$. The size of each area for motion estimation during video denoising was set by default to $q = 200$ pixels, but it can be customized by the user. The maximum number of the patches in each 3D group can affect the processing-time of the algorithm, so it was limited to 8 patches per group.

The minimum distance under which two blocks are assumed similar is highly dependent on σ_N, see Supplementary Equation (12). However, for $\sigma_N < 40$, it has been shown that the optimal value is 2500[18]. Furthermore, as stated above, λ_γ is another critical parameter for the denoising performance together with σ_N. Their product defines the threshold under which the coefficients of image in transform basis are set to zero. We have tested different values of λ_γ and found that the algorithm gives the best performance with $\lambda_\gamma = 4.0$ (see Supplementary Table 6).

Supplementary Table 6: Optimal value of the parameter λ_γ calculated using the simulated image shown in Supplementary Figure 12. The optimal λ_γ was chosen as the one that maximizes both the peak signal-to-noise ratio (PSNR) and the Structural similarity index (SSIM) for the restored image. The maximum values are marked in red.

	**SNR (before denoising)**					
	0.5		1		2	
λ_γ	PSNR (dB)	SSIM	PSNR (dB)	SSIM	PSNR (dB)	SSIM
**2.7**	33.41	0.705	31.88	0.742	31.11	0.792
**4.0**	33.71	0.712	32.70	0.804	32.09	0.864
**5.4**	33.53	0.674	32.61	0.779	32.15	0.856

3.2 Resolution

A deficiency of many existing denoising algorithms is that less-than-ideal filtering leads to the loss of fine details in the image. For example, when using low-pass filtering, a thorough noise correction can come at the expenses of resolution[22]. This is because some of the high frequencies of the sample can be eliminated together with the noise frequencies.

To avoid such issues, ACsN uses self-similarity to sort noise from the features of the samples. We have tested it by comparing the image resolution before and after noise correction in both simulated and experimental data. The results confirmed that the algorithm does not induce any loss of resolution (see Supplementary Figures 15-17).

We used Gaussian fitting to evaluate the width of the point-spread function (PSF) and detected no additional blurring after to the denoising process. For fair comparison between

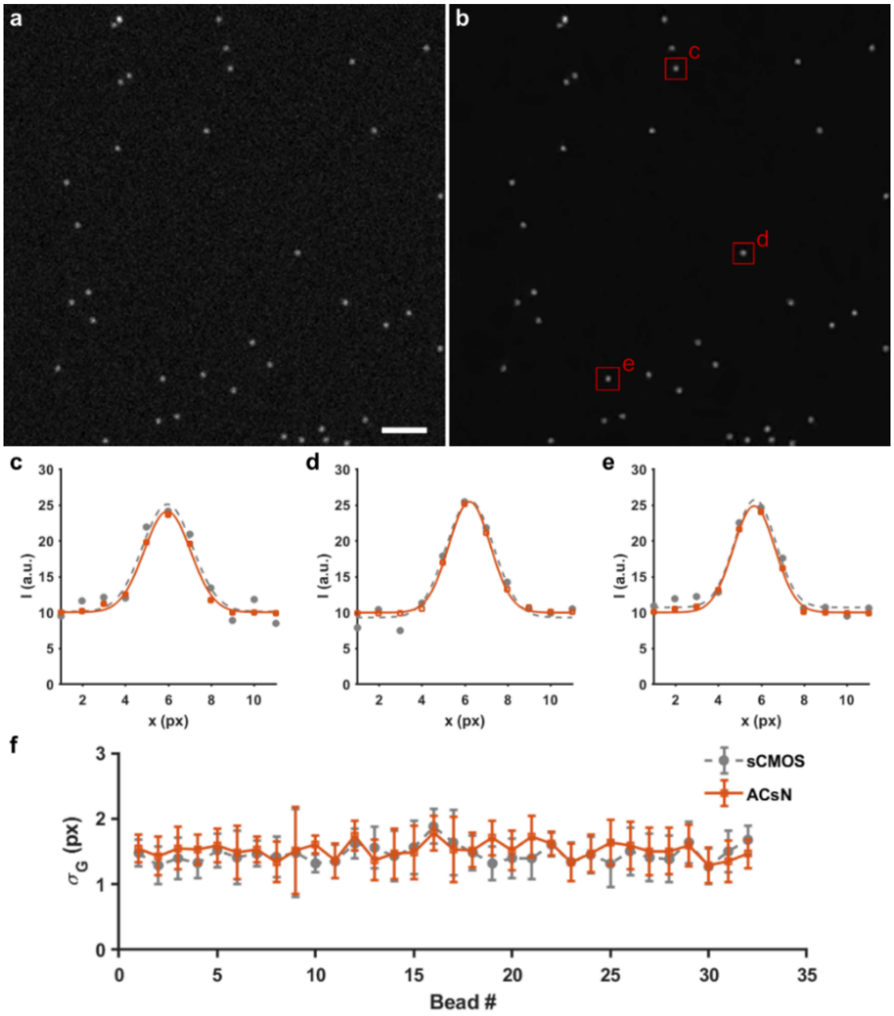

Supplementary Figure 15: Simulations show no loss of resolution in denoised images. (a) Simulated noisy sCMOS image of fluorescent beads. The image size is 128x128 pixels. (b) Denoised image using ACsN. (c-e) Intensity profiles of the beads marked in (b) obtained by averaging over the vertical dimension of each bead image. Gray and red dots represent the values obtained by images (a) and (b), respectively. For each set of points, the corresponding Gaussian fit is plotted. (f) Plot of the standard deviation (σ_G) of the bead profiles obtained by Gaussian fitting in both (a) and (b). The error bars represent the confidence range of the fitting algorithm. Scale bar: 25 pixels.

noisy and corrected data, the intensity profiles were generated by averaging over the vertical dimension of each bead image.

It is important to notice that both the simulations and the experimental images used in the resolution assessment are strongly non-uniform and, therefore, feature a highly spatially-variant noise. However, this did not affect either the performance of ACsN nor the final resolution of the denoised images.

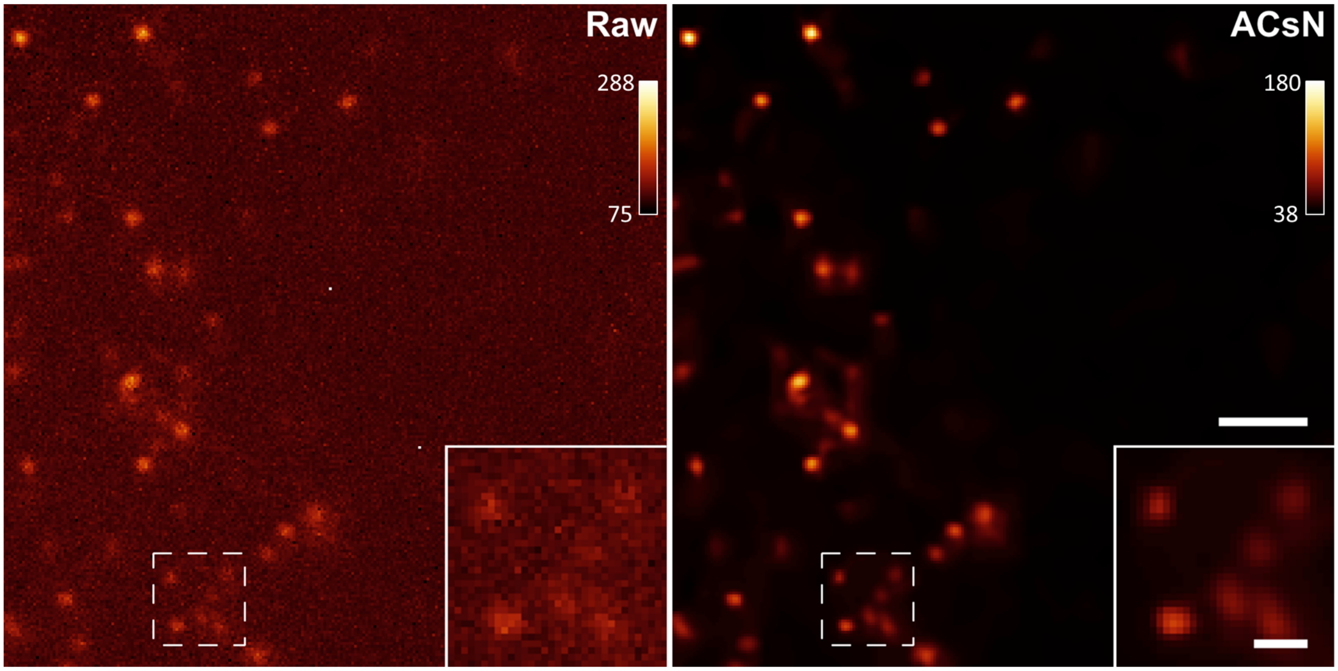

Supplementary Figure 16: Resolution preservation in single molecules frames. Representative single molecule sCMOS image of Alexa Fluor 647 before (left) and after (right) denoising with ACsN. It can be noted that the sample is strongly spatially-variant. However, such non-uniformity of the image does not affect the performance of ACsN and, thus, resolution of the image. Scale bar: 1µm, 500 nm (inset).

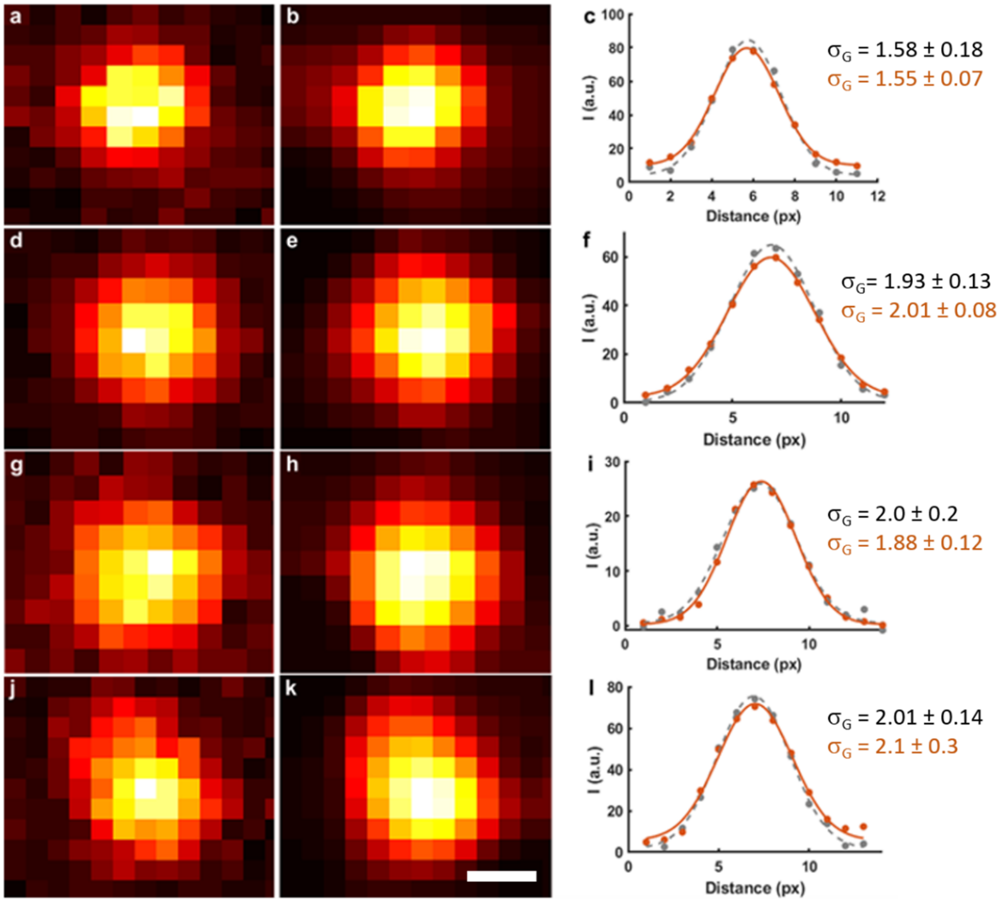

Supplementary Figure 17: Experimental images of single molecules. (a,d,g,j) Noisy sCMOS images of Alexa Fluor 647. (b,e,h,k) The corresponding single-molecule images after denoising with ACsN. (c,f,i,l) Intensity profiles generated by averaging over the vertical dimension of each molecule image. Gray and red dots represent the values obtained by images before and after ACsN processing, respectively. For each set of points, the corresponding Gaussian fit is plotted and the relative standard deviation (σ_G) for both raw (in black) and denoised (in red) images is reported on the side. Scale bar: 200 nm.

3.3 Reduction of pixel fluctuation

A key feature of the camera noise is that it induces fluctuations at every pixel over time. This effect can become prohibitive for many imaging applications at low light levels and detrimental for quantitative analysis of time-lapse data. An effective noise correction approach should address this issue to maximally reduce pixel fluctuations that are not related to the sample.

The fluctuation of each pixel, σ, can be measured as the standard deviation of its value in time. The standard deviation map of time-lapse sCMOS frames before and after noise correction using ACsN shows a substantial reduction of the temporal fluctuation of the pixel values. This reduction is especially prominent in the background pixels, where no signal is expected. Residual fluctuation is clearly separated from the background by the algorithm and can be observed in correspondence of the sample. However, this residual fluctuation can be more than one order of magnitude weaker than the fluctuation observed in the raw images.

Recalling that the signal of an ideal camera is still affected by fluctuations due to the photon shot noise (see Supplementary Note 1), we have compared the pixel fluctuation of our sCMOS camera after ACsN processing with the expected value of an ideal one, $\sigma_P = \sqrt{S}$ (see Supplementary Figure 18). The signal, S, is obtained for each image by averaging 100 frames acquired at low frame rate (9 Hz).

As expected, the magnitude of fluctuation in the raw images increases with the frame rate, and, in turn, with the predominance of sCMOS-related noise due to the low intensity of the fluorescence signal (see gray squares in Supplementary Figure 18c, f and i). However, after ACsN processing, the magnitude of fluctuation is visibly lowered and becomes comparable to the expected values of an ideal camera, $\sigma/\sigma_P \sim 1$ (see red circles in Supplementary Figure 18c, f and i). In some cases, when the video filtering is active, the denoising performance can be improved so that it can achieve even a supra-ideal performance because both readout and photon noise contributions have been corrected in the algorithm ($\sigma/\sigma_P < 1$).

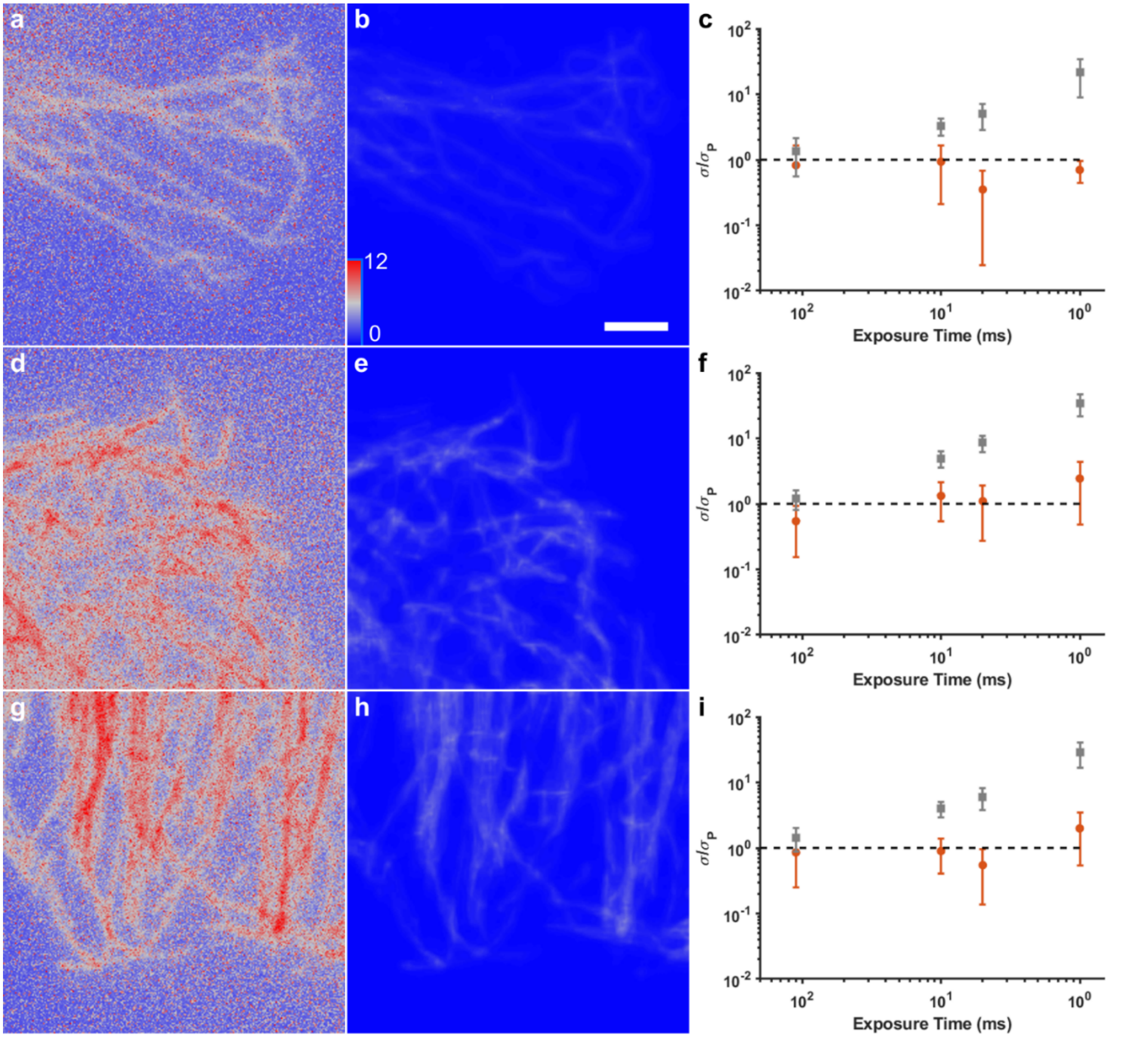

Supplementary Figure 18: Pixel fluctuation after ACsN processing is comparable to that of an ideal camera. (a,d,g) Fluctuation maps calculated over 100 frames of three different images of microtubules acquired with 1-ms exposure time. (b,e,h) Fluctuation maps of the same image stacks after ACsN processing. (c,f,i) Pixel fluctuation due to camera noise in raw images becomes more and more relevant with the frame rate (gray squares). This effect is strongly mitigated by ACsN processing (red circles), which maintains pixel fluctuation closer to the value contributed by only the photon shot noise, i.e. as in the ideal case (dashed black line). Points and error bars in the plots represent respectively the mean and standard deviation of the σ/σ_P ratio calculated over fluctuation maps corresponding to different exposure times. In some cases, such as when the video filtering is active, ACsN shows an improved denoising efficiency, resulting in the presence of values of $\sigma/\sigma_P < 1$. Scale bar: 3 µm.

3.4 Dependency on the incoming photon flux

The challenge for denoising in cell imaging is being able to obtain a thorough correction of the noise while preserving sample's details and photon count. Therefore, the performance of noise correction is intrinsically related to the photon flux of the input image. Indeed, at low photon counts, such details start to be comparable to the noise levels and, most of all, the fluctuations of the photon shot noise become significant.

In this section, we assessed the dependence of the denoising performance on the intensity of the emission light and the corresponding improvement of the image quality and SNR of ACsN image restoration. First, we assessed the performance of image restoration using simulations in order to observe the robustness of ACsN with the two main sources of camera noise, i.e. readout noise and photon noise.

We used four simulated sequences of fluorescent Siemens stars to predict the ACsN performance with different photon fluxes. The maximum number of photons for each sequence was 5, 10, 15, and 20 photons per pixel (see Supplementary Figure 19a). In all the cases, we observed a good noise correction of the mixed Poisson-Gaussian noise (see Supplementary Figure 19b). In particular, the average quality of the denoised images shows few variations down to ~10 photons per pixel or less, with some loss of quality only when the photon flux is less than 5 photons per pixel (see Supplementary Figure 19c-d).

Using the standard caliber target and a Hamamatsu ORCA Flash 4.0 v3 sCMOS camera, we observed a similar trend with respect to the simulations, with a valid performance of ACsN down to ~10 photons per pixel (see Supplementary Figure 20). In addition, similar results were observed using biological samples and the same camera (see Supplementary Figure 21).

Thus, we see a robust noise correction down to ~10 photons per pixel in both simulations and experimental data. In particular, simulations show a good correction of the photon noise within this intensity range, supporting the validity of our model. When the photon flux is very low (~3 photons per pixel), the Poisson distribution diverges from its Gaussian approximation and the denoising could fail to correct the photon shot noise. At the same time, the relative variation of readout noise and photon shot noise in the raw data is such that, when the photon flux is <10 photons per pixel per frame, the SNR becomes very low. For example, the intrinsic error of the signal given by the photon shot noise is $\sigma_P = \sqrt{S}$ >30%, as described in Supplementary Equation (2) in Supplementary Note 2. In addition, for most sCMOS cameras the readout noise is stronger at high frame rates (typically at 100 Hz), so that the image degradation at a short exposure time is a result of both lower photon budget (i.e. stronger noise influence) and worse camera performance.

Therefore, when the photon budget is low, the details of the underlying structure of the sample are largely submerged by the noise, making it challenging to generate a representation where the signal is sparser than noisy pixels, and hindering further image restoration (see Supplementary Note 3.5). For this reason, and since a realistic sample is not uniform, we consider a median flux of 10 photons per pixel as the practical limit for ACsN denoising applications.

It should be noted that the emission photon flux in fluorescence microscopy can vary depending on both the power of the excitation light and the specific fluorophore observed. Different examples of the performance of ACsN in many techniques of fluorescence microscopy are shown in the Results section in the main text.

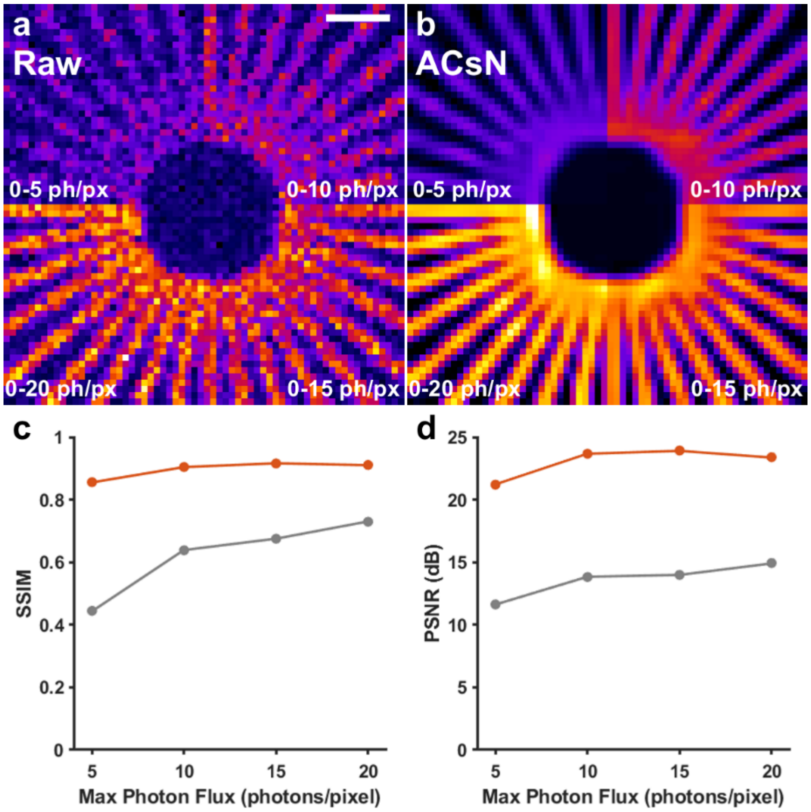

Supplementary Figure 19: ACsN image restoration of simulated data. Representative frames of 4 sequences of 100 simulations of a fluorescent Siemens star in presence of mixed Poisson-Gaussian noise before (a) and after (b) ACsN denoising. The maximum photon flux for each sequence (clockwise from the top left quadrant to the bottom left quadrant) is 5, 10, 15, and 20 photons per pixel, respectively. Plots of the average SSIM (c) and PSNR (d) calculated for each sequence of the simulated data before (gray) and after (red) ACsN denoising. The error bars represent the corresponding standard deviation and are smaller than the size of the markers. Scale bar: 1 μm.

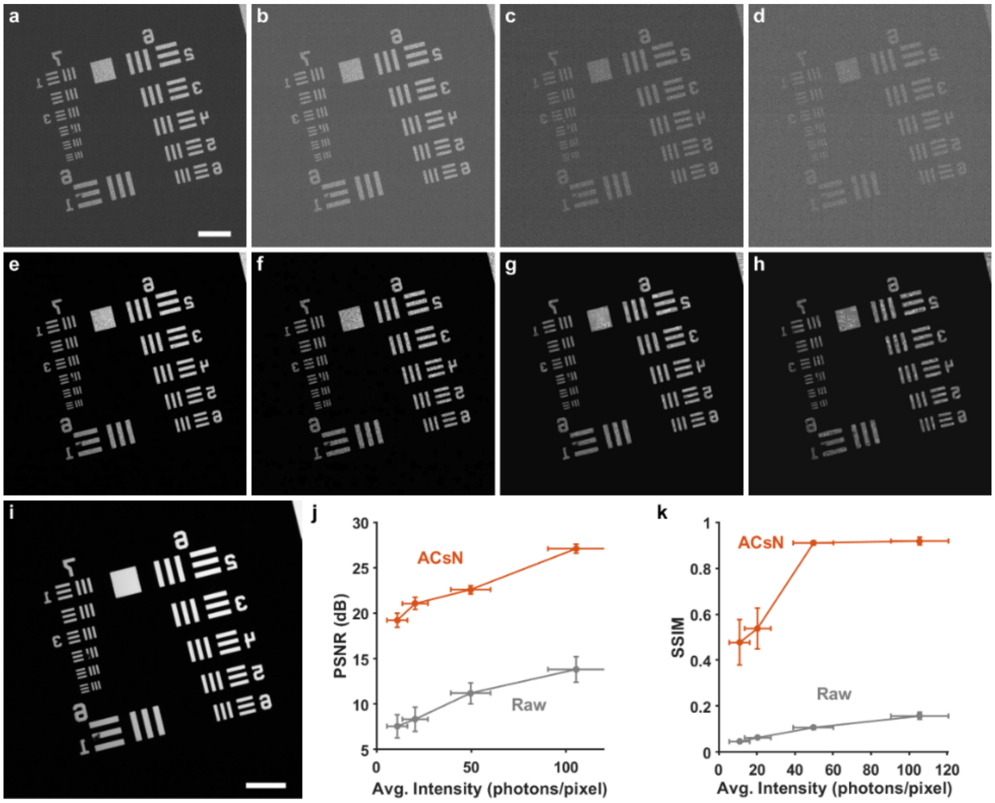

Supplementary Figure 20: ACsN image restoration of caliber structures at varying light levels. (a-d) Illustrative frames of four sequences of 50 images of a fluorescent USAF target. Each sequence was acquired with the same illumination intensity but at a different exposure time: 10 ms (a), 5 ms (b), 2 ms (c), and 1 ms (d). (e-h) The corresponding denoised images of (a-d) using ACsN, respectively. (i) Reference frame used as the ground truth. This frame was obtained by averaging 50 frames acquired with a 100-ms exposure time. (j,k) Plots of the PSNR and the SSIM as a function of the emission intensity. For fair comparison, the emission intensity was evaluated as the average intensity calculated only in the bright (fluorescent) areas of the USAF target and after offset and gain correction. The vertical error bars represent the standard deviation of the PSNR (j) and the SSIM (k). The horizontal error bars represent the standard deviation of the evaluation of the emission intensity. Scale bars: 50 μm.

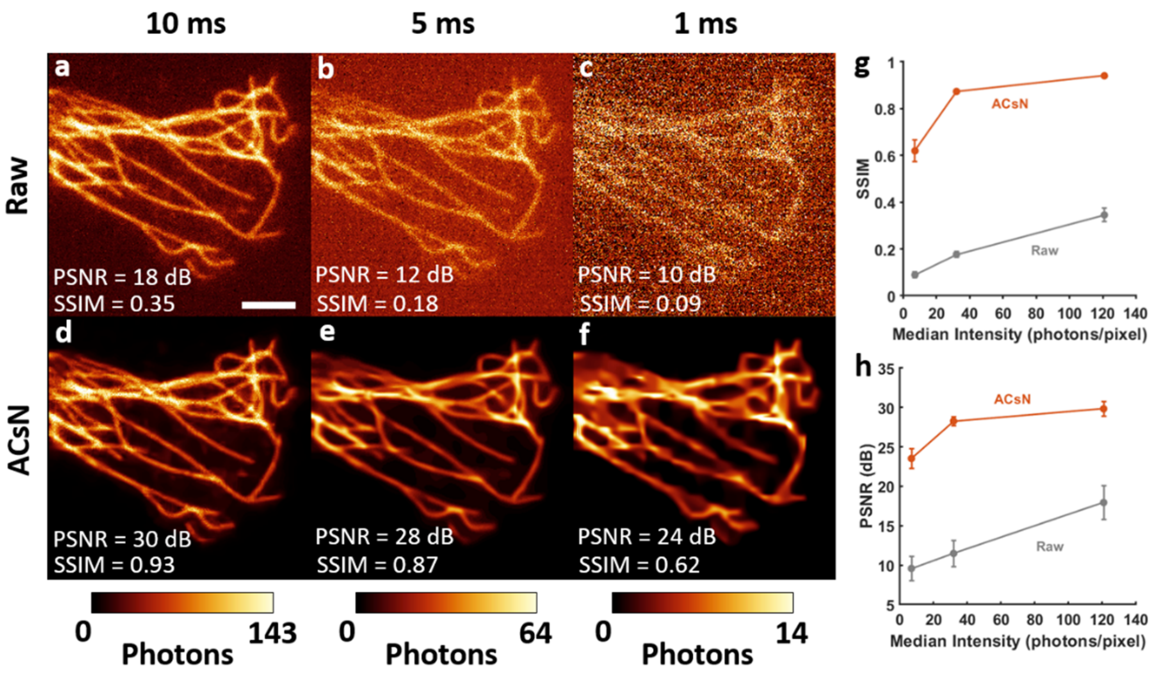

Supplementary Figure 21: ACsN image restoration of biological samples at varying light levels. Three sequences of 100 images of microtubules of HeLa cells were recorded using TIRF microscopy with the same illumination intensity but at different exposure times: 10 ms (a), 5 ms (b), and 1 ms (c). Each series was processed with ACsN. A representative frame for each sequence is shown in (d-f), respectively. It can be observed that noticeable improvement in image quality can be recovered at low light levels (e.g. <15 photons per pixel in (c)), despite the loss of some fine details. (g, h) The plots of the SSIM (g) and the PSNR (h) as a function of photon counts per pixel. Each point represents the average value obtained from each sequence of 100 images and each error bar represents the corresponding standard deviation. The quantitative measurement shows substantial improvement in image quality using ACsN, as well as the influence of the photon flux in the restoration, consistent with the observations in (d-f). Scale bar: 3 μm.

3.5 Sampling rate

In a diffraction-limited optical system, the sampling frequency should be strictly higher than the Nyquist frequency[23], which means that the pixel size of the image should be preferably selected to be no more than half of the system resolution. However, this is not always the case, e.g. when a wider field-of-view is preferred to an optimal resolution. In this section, we investigated the performance of the ACsN algorithm under various sampling rates. We define the sampling rate as the ratio between the system resolution, given by the Rayleigh criterion ($0.61\lambda/NA$) and the effective pixel size of the image (physical sensor pixel size divided by the magnification), so that the minimum sampling rate according to the Nyquist criterion should be greater than 2.

Using numerical simulations, we found that ACsN could tolerate a wide range of sampling rates from 1.2 to 2.9 (see Supplementary Figure 22a-f). Over-sampling can improve the self-similarity in the image. However, it also lowers the photon flux per pixel, thus decreasing the SNR of the original frames, consequently enhancing the influence of the noise (see 3.4 Dependency on the incoming photon flux). Thus, the denoising performance is a balance between the improvements in self-similarity and the loss of SNR (see Supplementary Figure 22g). On the other hand, the correction performance gradually decreases also when the image is significantly under-sampled, i.e. sampling rate less than 1.5 (see Supplementary Figure 22h). This is because with under-sampling the observed object tends to a delta function. As a result, it has a less sparse representation in the transform domain and, frequency-wise, it becomes more and more similar to a noisy pixel. However, this effect is mitigated by an improvement

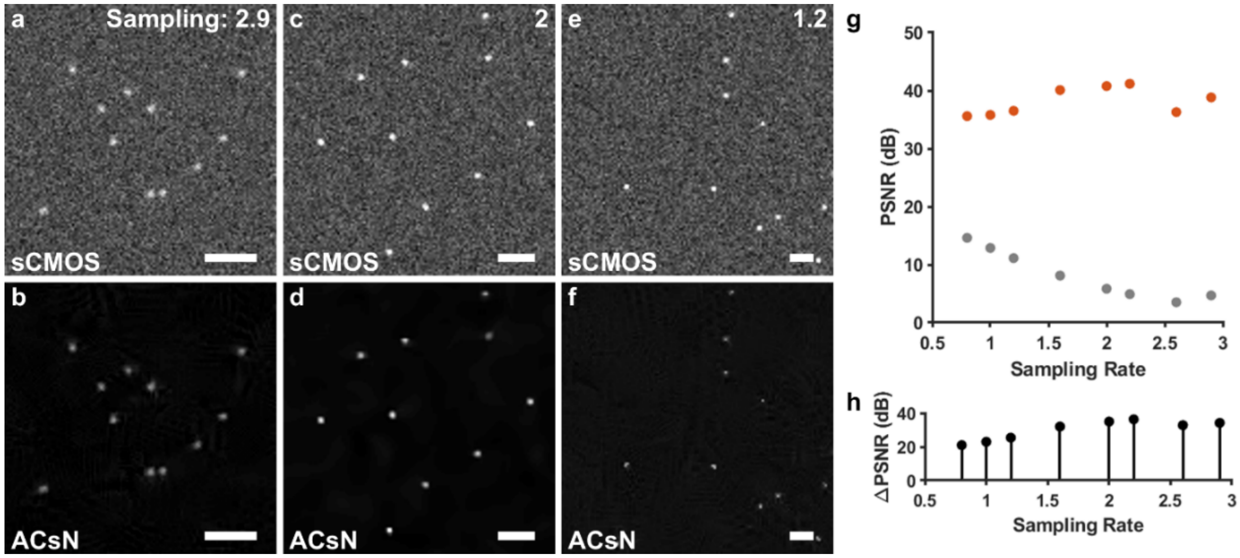

Supplementary Figure 22: ACsN denoising performance at different sampling rates. Simulated sCMOS frames and relative denoised images for various sampling rates of the detector, namely: 2.9 (a,b), 2 (c,d), and 1.2 (e,f). (g) Plot of the PSNR of simulated noisy images of beads before and after denoising, respectively in gray and red dots. (h) PSNR improvement after ACsN denoising at different sampling rates. Scale bars: 2 μm.

of the SNR of the original image correlated to the under-sampling (see Supplementary Figure 22g).

Likewise, experimental data show the robustness of ACsN for low SNR with oversampling and no significant loss of signal with under-sampling (see Supplementary Figure 23). It is important to notice that although the improvement of self-similarity due to over-sampling balances the loss of SNR, we observe a similar or better denoising performance at lower sampling rates (see Supplementary Figure 23e-f). Indeed, under-sampling increases the number of photons per pixel, thus improving the SNR of the input frames (see Supplementary Figure 24a-c). Here, the enhanced SNR compensates for the lower self-similarity so that under-

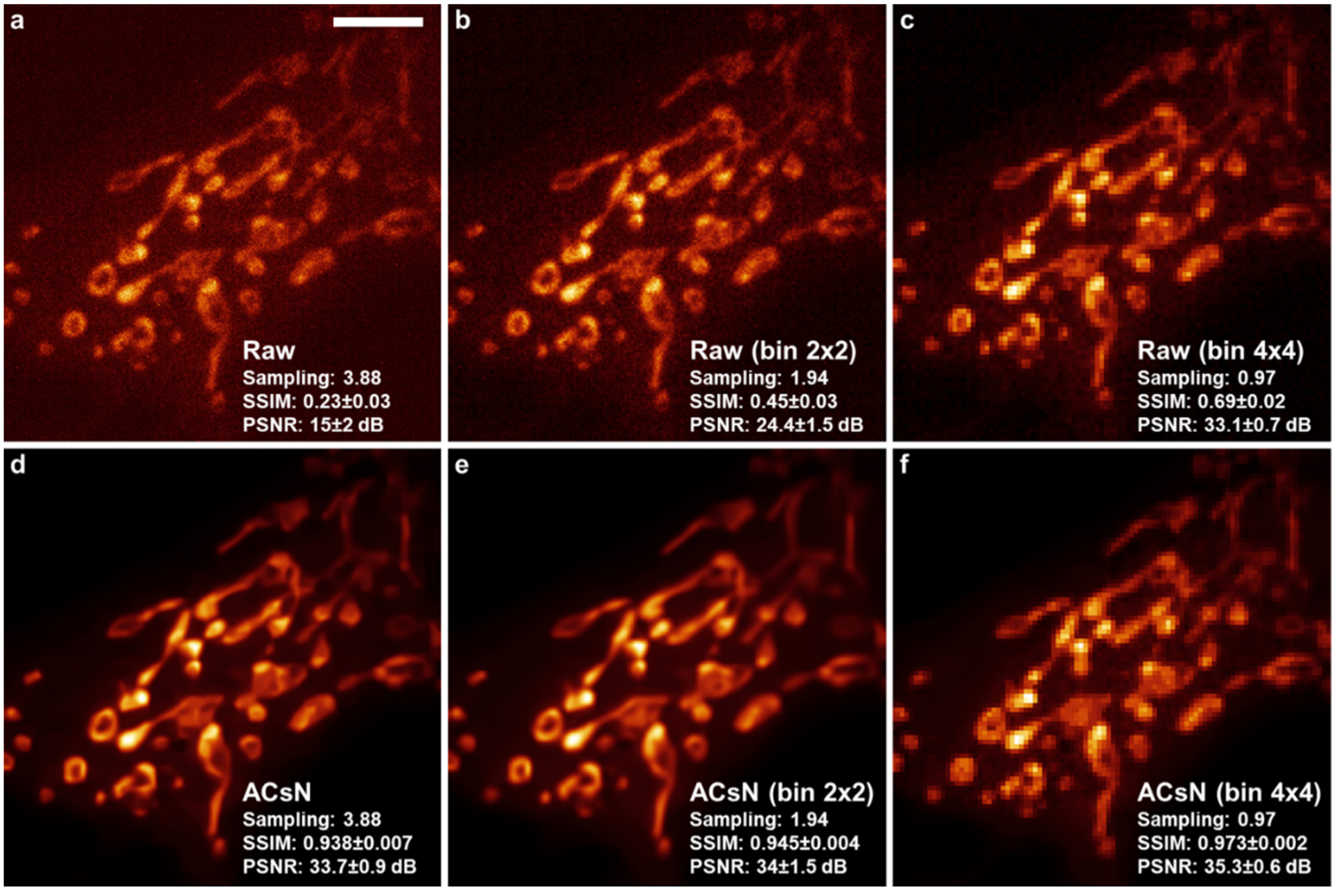

Supplementary Figure 23: ACsN denoising performance with different sampling rates. (a,d) Representative frames before (a) and after (d) ACsN denoising of a sequence of 100 frames of fixed mithocondria in bovine pulmonary artery endothelial (BPAE) cells. The images were acquired using 100× objective (NA 1.45), the fluorescence emission peak wavelength was 599 nm, and the camera pixel size 6.5 μm. This is well over-sampled with a sampling rate of 3.88. (b,e) Representative frames of the same sequence in (a) after a 2x2 pixel binning of the raw data before (b) and after (e) denoising. This effectively halves the sampling rate. (c,f) Representative frame of the same sequence in (a) after a 4x4 pixel binning of the raw data before (c) and after (f) denoising. It should be noted that even if the over-sampling in (a) and (d) is expected to enhance the image self-similarity, the image quality is also affected by the loss of SNR. On the other hand, the under-sampling in (c) and (f) benefits from the SNR improvement due to the higher photon count per pixel and shows better SSIM and PSNR values, although the performance is affected by the loss of signal details. The reference images for each sampling rate were obtained by averaging the image series of the corresponding sampling rate. Scale bar: 5 μm.

sampling does not seem to affect the quality of the reconstruction (see Supplementary Figure 24d-f). However, the image details are reduced by the larger pixel size.

In summary, experimental data together with the results obtained by the simulations support the viability of ACsN processing in a wide range of the sampling rate normally adopted for most fluorescence microscopy. The upper limit for range of acceptable sampling rates is mainly related to the photon flux necessary to maintain a feasible SNR of the input data. Whereas, the lower limit is given by the cost in terms of loss of details. For these reasons, a sampling rate as close as possible to the Nyquist limit is generally recommended as a good tradeoff between SNR and detail preservation.

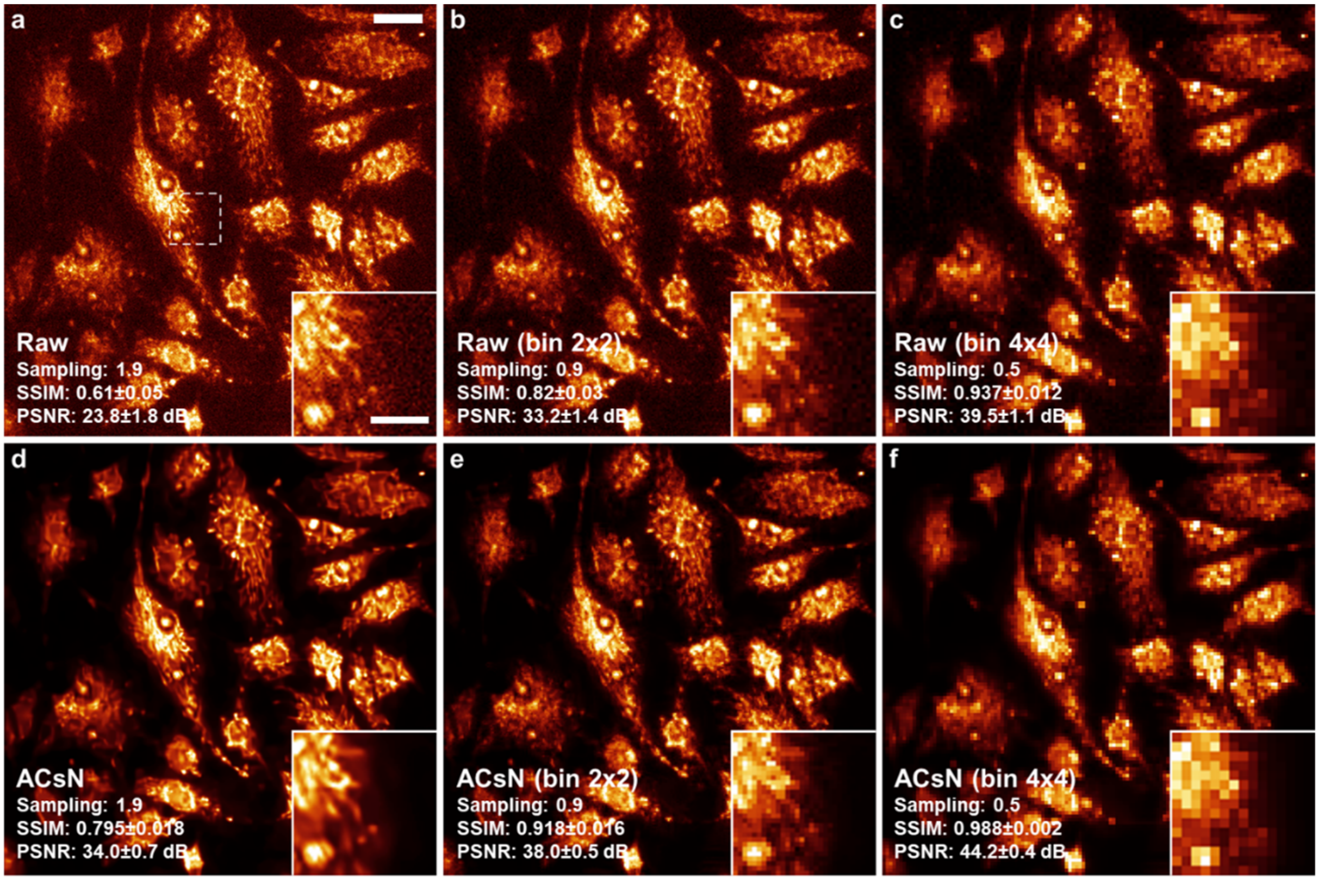

Supplementary Figure 24: ACsN denoising performance with under-sampling. (a,d) Representative frames before (a) and after (d) ACsN denoising of a sequence of 50 frames of fixed mithocondria in bovine pulmonary artery endothelial (BPAE) cells. The images were acquired using 10× objective (NA 0.3). The peak wavelength of the fluorescence emission was 599 nm, and the camera pixel size 6.5 μm. (b,e) Representative frames of the same sequence in (a) after a 2x2 pixel binning of the raw data before (b) and after (e) denoising. (c,f) Representative frames of the same sequence in (a) after a 4x4 pixel binning of the raw data before (c) and after (f) denoising. The under-sampling improves the SNR, largely suppressing the noise in (c) and (f). However, this comes at the expenses of a loss of details, which is evident in the insets compared to (a,b,d,e). The reference images for each sampling rate were obtained by averaging the image series of the corresponding sampling rate. Scale bars: 50 μm (a), 20 μm (a inset).

3.6 Self-similarity of the input image

Unlike natural images, fluorescent images of biological samples are highly specified, exhibiting precisely labeled molecular targets or structures in cells. Thus, each fluorescent image usually features only a specific object recurrent across the field of view, resulting in non-locally repeated self-similarity that can effectively enhance the performance of the ACsN algorithm (see Supplementary Figure 25). This repetition is used to generate a sparser representation of such features and, then, to improve the efficiency of denoising (see Supplementary Figure 25c and d). On the other hand, during the grouping phase, when the different patches are matched, there is *no prior assumption* about the similarity of the input image. Instead, the algorithm evaluates such similarity according to a predetermined metric, see Supplementary Equation (11) in Supplementary Note 2.3, and the estimated noise variation (see Supplementary Note 2.4). This means that only the patches that have enough similarity are grouped and processed collectively. So, if the input image has no self-similarity, all the patches are processed individually.

In this section, we demonstrate the dependence of the ACsN denoising performance on the usage of the self-similarity of an input image. To this end, it is important to notice that disabling the grouping step of ACsN is equivalent to the denoising of an image with no self-similarity. In addition, the noise level can decrease the self-similarity of the input image independently from the characteristics of the observed sample. For example, the image in Supplementary Figure 25e shows a relatively high level of noise and, as a result, a lower level of self-similarity than the real structure (see insets in Supplementary Figure 25a and e). In this case, only few patches are grouped together because patches that show a level of similarity below the threshold value are not forced to match (see Supplementary Notes 2.4 and 3.1). For this reason, when the image self-similarity is low, ACsN tends to perform as a non-collaborative sparse filter, which explains the similarity between Supplementary Figure 25f and g. Nonetheless, the extension of the search window in the temporal dimension can further improve the grouping efficiency, offering a better image restoration (see Supplementary Figure 25h and Supplementary Note 2.5). We observed the same behavior also with the different structures (lines, squares and numbers) of a USAF target (see Supplementary Figure 26). Once again, here we can see that the use of 2D and 3D grouping improves the performance of ACsN in comparison to non-collaborative filter (see Supplementary Figure 26).

We observed that self-similarity in multiple dimensions, and especially time, can be particularly beneficial for the efficiency of ACsN block matching and grouping (see Supplementary Figures 25 and 26). Furthermore, we did not observe the appearance of any feature artifacts due to imperfect matching between dissimilar patches. However, since biological samples are heterogeneous, the level of self-similarity can vary for different structures. For this reason, we implemented a no-reference metric of the denoising quality, which can help the users to assess any variability of the ACsN denoising performance (see Supplementary Note 7).

Finally, given the intrinsic redundancy of the labeling process, we expect collaborative filtering to be especially helpful with fluorescence imaging. Here, we observed this by using fluorescently stained microtubules, which are generally used as a reference structure because of their well-known features. Nonetheless, we have provided various examples of common structures imaged with fluorescence microscopy that have different features and dimensionality such as actin bundles, mitochondria, filopodia, lamellipodia, fluorescent particles, and photoswitching fluorescent dyes.

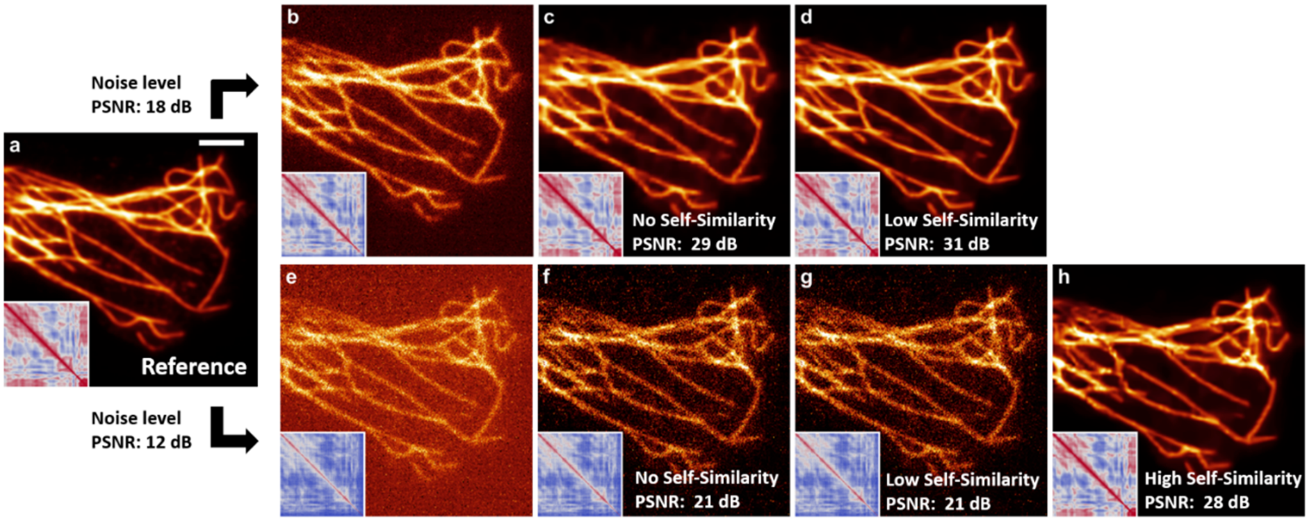

Supplementary Figure 25: Sample's self-similarity due to noise and ACsN denoising performance. (a) The reference image was obtained by averaging 100 frames of fixed microtubules of a HeLa cell recorded with an exposure time of 111 ms. (b,e) The same cell in (a) recorded with an exposure time of 10 ms and 5 ms, respectively, exhibiting different SNRs and thus influence of noise. (c,f) Denoised images of (b) and (e), respectively, obtained with ACsN without grouping (the maximum number of patches in each group was set to 1). Please, note that this is equivalent to use ACsN without considering self-similarity or an object with no self-similarity. (d,g) Denoised images of (b) and (e), repectively, obtained allowing 2D grouping. While (d) shows an improvement over (c), this does not exhibit as significantly for (g) over (f). This is related to the lower self-similarity of (e), due to the distruptive effect of the noise. The consistence between (f) and (g) also implies that the algorithm does not force any block matching where there is dissimilarity between patches. (h) The 3D grouping feature of ACsN compares patches in a bigger window, substantially improving the effectiveness of image restoration. The correlation maps of each image are reported in the corresponding insets. The values in the maps represent the Pearson's linear correlation coefficients calculated between each pair of columns. It is observed that an increased noise level leads to lower self-similarity. Scale bar: 3 μm.

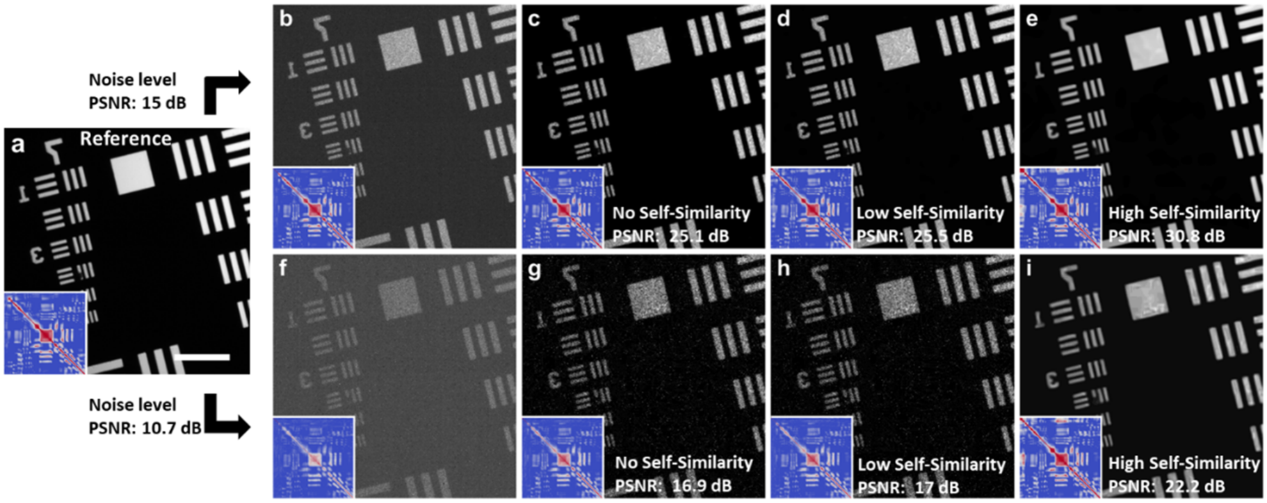

Supplementary Figure 26: Sample's self-similarity due to noise and ACsN denoising performance. (a) The reference image of a USAF target was obtained by averaging 100 frames with an exposure time of 100 ms. (b,f) The same region in (a) recorded with an exposure time of 10 ms and 2 ms, respectively. (c,g) Denoised images of (b) and (f), respectively, obtained with ACsN without grouping (the maximum number of patches in each group was set to 1). Please, note that this is equivalent to use ACsN without considering self-similarity or an object with no self-similarity. (d,h) Denoised images of (b) and (f), repectively, obtained allowing 2D grouping. While (d) shows an improvement over (c), this does not exhibit as significantly for (h) over (g). This is related to the lower self-similarity of (f), due to the distruptive effect of the noise, and shows how the algorithm does not force any block matching where there is dissimilarity between patches. (e,i) The 3D grouping feature of ACsN compares patches in a bigger window, substantially improving the effectiveness of image restoration, compared to (d) and (h), respectively. The correlation maps of each image are reported in the corresponding insets. The values in the maps represent the Pearson's linear correlation coefficients calculated between each pair of columns. It is observed that an increased noise level leads to lower self-similarity. Scale bar: 50 μm.

Supplementary Note 4. Additional application notes

4.1 Deconvolution microscopy

Image deconvolution is widely used in optical microscopy, from the restoration of low quality images to the improvement of super-resolution techniques[24]. However, the deconvolution process can be very sensitive to the noise in the input image, and a low SNR, or a non-appropriate estimation of the noise, can degrade the performance of many commonly used algorithms. In this section, we demonstrate the implementation of ACsN denoising can effectively prevent non-fluorophore-associated noisy pixels, thus significantly improving the subsequent downstream deconvolution analysis.

For example, Richardson-Lucy (RL) deconvolution is one of the most popular method appropriate to improve density estimates in fluorescence imaging[25,26]. RL deconvolution estimates the true density by an iterative procedure, improving the likelihood that the estimate is correct with every iteration assuming Poisson noise.

However, sCMOS-related noise can be detrimental for the effectiveness of RL deconvolution and can hinder any resolution recovery (see Supplementary Figure 27a, c, and e). On the other hand, using ACsN prior to deconvolution to reduce the noise enables the RL algorithm to perform in noise conditions closer to the ideal case of the pure Poisson noise (see Supplementary Figure 27b, d, and f). This is reflected also in a remarkable improvement of the Resolution Scaled Pearson coefficient (RSP), which provides a score of the global image quality[27].

Another popular analytical method for image restoration is super-resolution radial fluctuations (SRRF)[28]. This method allows the user to achieve super-resolution using wide-field, confocal or TIRF microscopes with illumination orders of magnitude lower than many super-resolution techniques. Here, the analysis of a sequence of images acquired in a standard wide-field or TIRF microscope directly reconstructs a super-resolution image without fluorophore detection and localization. SRRF assumes the image is formed of point sources convolved with a PSF that displays a higher degree of local symmetry than the background. Unlike single-molecule localization microscopy (SMLM), SRRF calculates the degree of radiality across each frame in an image sequence on a sub-pixel basis. This radiality distribution is capable of distinguishing two Gaussian PSFs separated by ~0.7 times the Gaussian standard deviation.

However, the radiality map of a full image includes also a number of non-fluorophore-associated radiality peaks, as transient local radial symmetries can occur in image noise, especially when using low-intensity illumination (see Supplementary Figure 2a-b). This issue is usually addressed by frame averaging to generate a super-resolution frame from an image sequence. Typically, to generate one single super-resolution image, a sequence of at least 100 frames is required (see Supplementary Figure 2c). The maximum temporal resolution reported is 1 Hz[28].

On the contrary, ACsN can reduce the non-fluorophore-associated peaks from wide-field images without any temporal averaging. Therefore, a high-quality super-resolution image can

be readily obtained with only a single ACsN-processed frame (RSP 0.927, see Supplementary Figure 2d-i). This implies that ACsN processing can enable us to use SRRF and wide-field microscopy to generate super-resolution images with up to two orders of magnitude of improvement in temporal resolution. Here, we demonstrated the possibility to obtain super-resolution images using frames recorded at 200 Hz, which is 200 times faster than currently reported.

Lately, an alternative approach for image restoration based on machine learning has been proposed[29]. This consists in the development of content-aware image restoration (CARE) networks, which are adapted to a specific experimental setup. This strategy is based on the hypothesis that they produce better results than content-agnostic methods.

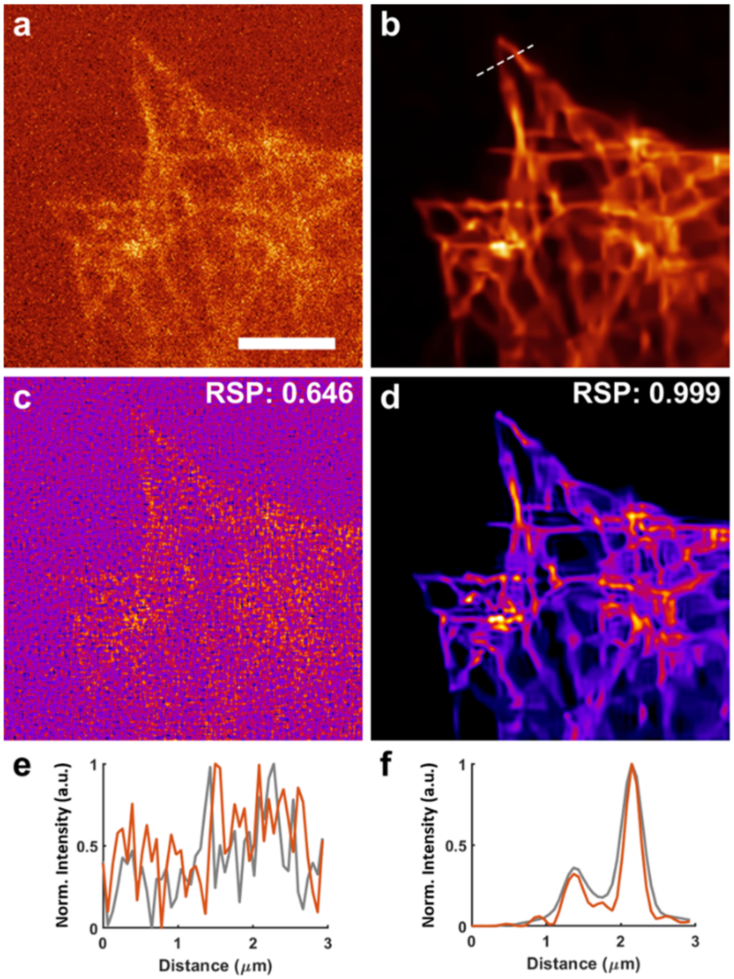

Supplementary Figure 27: ACsN improves Richardson-Lucy (RL) deconvolution. (a) TIRF image of microtubules aquired with an exposure time of 5 ms. (b) ACsN-processed image of (a). (c,d) Deconvolved images of (a) and (b), respectively. Both deconvolutions were performed using the same RL algorithm and 10 iterations. (e,f) Profiles of two microtubule filaments corresponding to the dashed line in (b), respectively without (a,c) and with (b,d) ACsN processing. Gray and red lines in (e) and (f) correspond to the profiles before (a,b) and after (c,d) RL deconvolution, respectively. The corresponding Resolution Scaled Pearson (RSP) coefficients were calculated and shown for (c) and (d). Scale bar: 4 μm.

A key point for the reusability of CARE is the possibility to use synthetic data to train the network for the restoration of a certain type of image. For example, using synthetic ground-truth images of tubular meshes resembling microtubules, it is possible to obtain super-resolution frames of microtubules from wide-field images of comparable or better quality than SRRF.

However, also in this case, the quality of image restoration deteriorates when noise becomes prominent. Even with low levels of noise, some image details start to disappear until a drastic failure of the image recovery in presence of stronger noise (see Supplementary Figure 28a-h). ACsN processing can recover low-SNR images (see Supplementary Figure 28i-l), thus enabling a subsequent CARE reconstruction for low-light-level, high-frame-rate, noisy images (see Supplementary Figure 28m-t). This is confirmed by a higher image quality of the reconstructions (RSP > 0.94) and an overall improvement of the reliability of the image restoration, which is also reflected by the general reduction of the ensemble disagreement across the images (see Supplementary Figure 28 and Supplementary Table 7). The evaluation of the ensemble disagreement is provided by the CARE software for each pixel of the deconvolved images. In Supplementary Table 7, the mean, maximum and mode of the disagreement values for each image in Supplementary Figure 28 are reported. In all the cases, the use of ACsN prior to CARE results in lower ensemble disagreement, in accord with the improvement of the RSP values.

In addition, it should be noted that the values obtained for Supplementary Figure 28h represent an exception as a result of the failure of an accurate reconstruction. This is due to the intrinsic bias of the disagreement score rather than the effective image quality, because the score evaluates the internal variance of the result but not the absolute accuracy.

Indeed, to use CARE for denoising, one should train the network with sufficient noisy data. This is actually both an essential and a challenging step in practice for learning-based systems[30]. Here, we showed that the use of ACsN prior to CARE enables high-quality image restoration even when the training on low-SNR data is lacking or insufficient.

In summary, here we have demonstrated the advantages of combining ACsN denoising with three freely-available deconvolution packages, including CARE deconvolution based on synthetic learning. This is particularly interesting because it allows using a pre-trained network for learning-based deconvolution in a wider range of imaging conditions, provided that the optical system is compatible. For further discussions about the ACsN and CARE denoising, please refer to Supplementary Note 8.

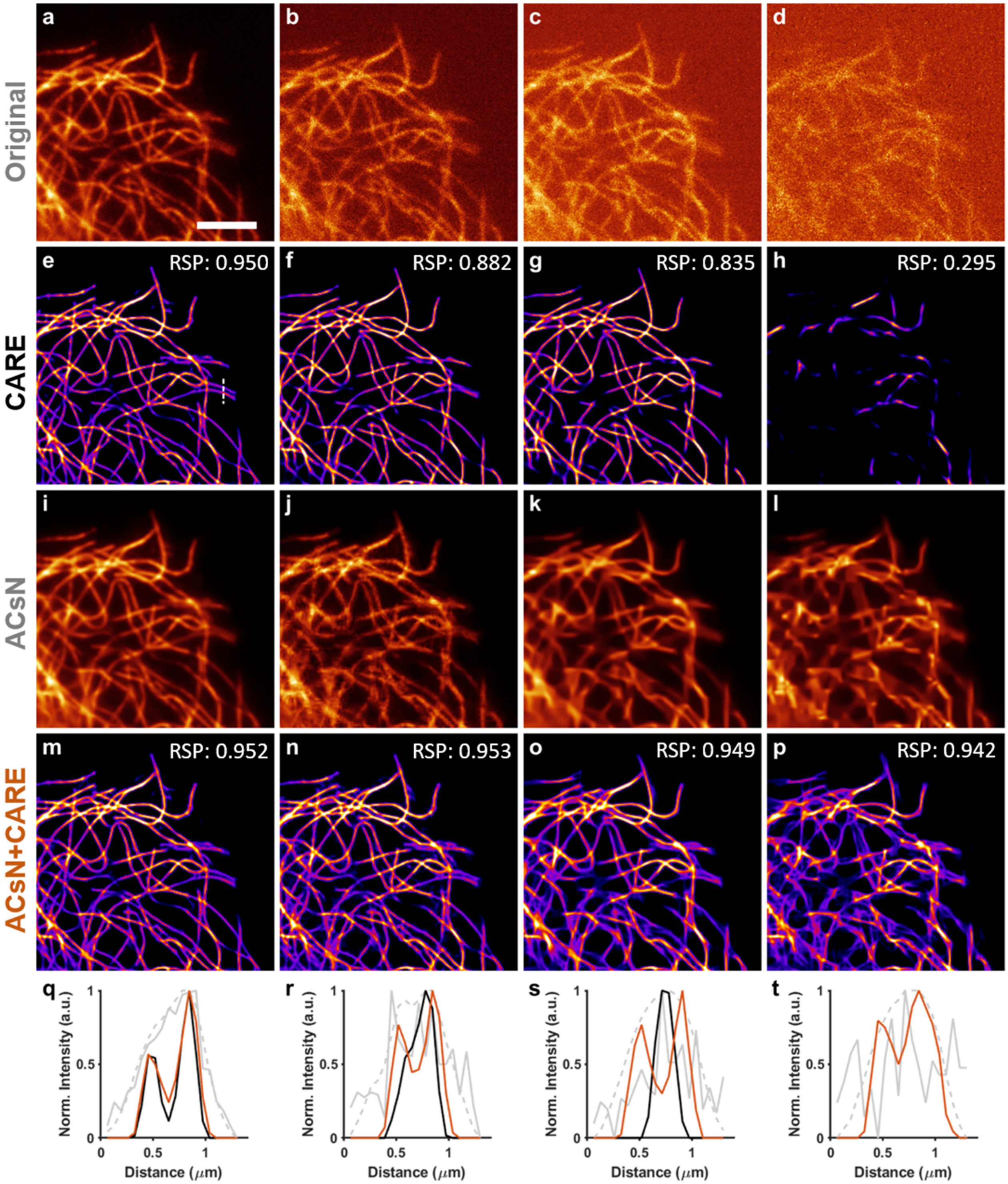

Supplementary Figure 28: ACsN denoising improves the accuracy of machine learning based deconvolution. TIRF images taken from four sequences of fixed microtubules acquired with different exposure time: (a)111 ms, (b) 10 ms, (c) 5 ms, and (d) 1 ms. (e-h) Deconvolved images obtained averaging 10 frames processed with CARE from the sequences in (a-d), respectively. Loss of details can be noticed in (f) and (g) in comparison with (e), while in (h) CARE completely fails to reveal the object. The corresponding worsening RSP values are shown. (i-l) ACsN denoising of sequences in (a-d), respectively, maintaining consistent image quality for different exposure times. Each image was obtained without frame averaging. (m-p) Deconvolved images obtained averaging 10 frames processed with CARE from the sequences in (i-l), respectively, recovering previously missing details. The corresponding consistent RSP values are shown. (q-t) Profiles of two microtubules corresponding to the white dashed line in (e). In each plot are shown the profiles measured in the original images (gray solid line), CARE (black line), ACsN (gray dashed line) and ACsN+CARE (red line). As seen, using ACsN prior to CARE enhances the quality of the deconvolution against noise and mitigates the loss of details. Scale bar: 4 μm.

Supplementary Table 7: Reliability of the learning-based deconvolution. Here are reported the mean, maximum and mode of the ensemble disagreement evaluated by CARE for each pixel of the deconvolved images in Supplementary Figure 28.

	**Ensemble Disagreement**					
	Raw + CARE			ACsN + CARE		
**t_{exp} (ms)**	Mean	Max	Mode	Mean	Max	Mode
**111**	0.0235	0.280	5.46e-4	0.0227	0.249	4.87e-4
**10**	0.0353	0.448	8.75e-4	0.0284	0.273	5.33e-4
**5**	0.0399	0.477	9.32e-4	0.0275	0.275	5.38e-4
**1**	0.0073	0.397	7.76e-4	0.0241	0.241	9.43e-4

4.2 Single-particle tracking

Single-particle tracking (SPT) is a key technology that provides researchers with the ability to directly observe molecular behaviors at the level of single molecules in living cells under certain limitations of SNR and spatial and temporal resolutions[31]. SPT provides access to single molecule behavior by detecting and following individual particles in a time series of images. However, the usefulness of the trajectories obtained depends on localization precision, temporal resolution and observation time. These three factors are interconnected and ultimately coupled to the photon budget delivered by the fluorescent label[32].

To demonstrate ACsN in SPT, we recorded the movement of a fluorescent bead diluted in water. The concentration of beads was low in order to avoid ambiguities in particle localization and simplify the tracking task. This way, any eventual error in particle localization could be ascribed to the low SNR. We analyzed a sequence of 150 frames acquired with an exposure time of 1 ms before and after ACsN processing (see Supplementary Figure 29a and b respectively, Supplementary Movie 3) using standard nearest-neighbor linking[33]. From the plot in Supplementary Figure 29c, it can be observed that localization errors and gaps in the trajectory (crimson diamonds) are related to the low SNR of the raw data. The ACsN-denoised sequence, instead, shows an enhanced SNR and no gaps in the trajectory (see Supplementary Figure 29c, black line). This is important because localization errors are more probable when the level of noise is comparable to the brightness of the observed particle. In fact, the tracking obtained using the raw data resulted in 6 different trajectories (see Supplementary Figure 29d) due to the high noise level that led to missed localizations in certain frames. In contrast, the denoised sequence yielded one single track of the entire 150 time points (see Supplementary Figure 29e). The reduction of image noise and the subsequent improvement of SNR provide a better accuracy of the particle localization and then a better estimation of the bead's lateral displacement with sub-pixel sensitivity (see Supplementary Figure 29f-h). This is in agreement with an improvement of the localization precision that we observed using a simulated sequence of fixed beads (see Supplementary Figure 30a-c). The tracking of the denoised data yielded values of the frame-by-frame displacement more peaked around the true value (zero) than the noisy sequence (see Supplementary Figure 30d). Indeed, there is a significant decrease of both the median displacement and the 3rd quartile after ACsN denoising (see Supplementary Figure 30e). We considered the outliers of all the values either 3 time the interquartile range (IQR) or more above the third quartile or 3 time the IQR or more below the first quartile.

To push further the performance of SPT, it is also possible to use ACsN in combination with more complex algorithms[34], even if that would imply a number of *a priori* assumptions about the sample and a more complicated data analysis.

Tracking molecular events in 3D is notably more challenging, but highly desirable in order to extend SPT experiments to intracellular or nuclear compartments. Therefore, much effort is focused on the implementation of optical set-ups, labeling strategies and dedicated data algorithms for tracking the motion of individual particles in 3D. Measuring the size and shape of the defocused intensity spot, it is feasible to infer the z-position of the particle. However,

with a conventional microscope, the absolute z-position (above or below the focal plane) cannot be unambiguously determined. Moreover, when the point source is close to the plane of focus, the resulting image profiles show negligible change in their shape thereby providing very little information about the z location.

Using biplane microscopy, the point source is simultaneously imaged at a second focal plane that is shifted by 500 nm from the first one[35,36]. This way, it is feasible to increase the localization precision in the axial direction even when the point source is close to the focal plane. In particular, the accuracy of the z-position determination remains relatively constant for a range of z values. Nonetheless, this accuracy depends on the precision to estimate the size of the intensity patterns in the image where the sample is defocused, which becomes deteriorated by the noise with the distance from the focal plane since the intensity patterns become weaker[37]. In fact, when these out-of-focus patterns become dimmer the localization precision depends greatly on the SNR of the image.

We have shown that the quality of the out-of-focus patterns can be maintained also in case of low SNR by ACsN denoising (see Supplementary Figure 31a-c, Supplementary Movie 4). This yields a substantial improvement of the SNR especially in the images where the particle

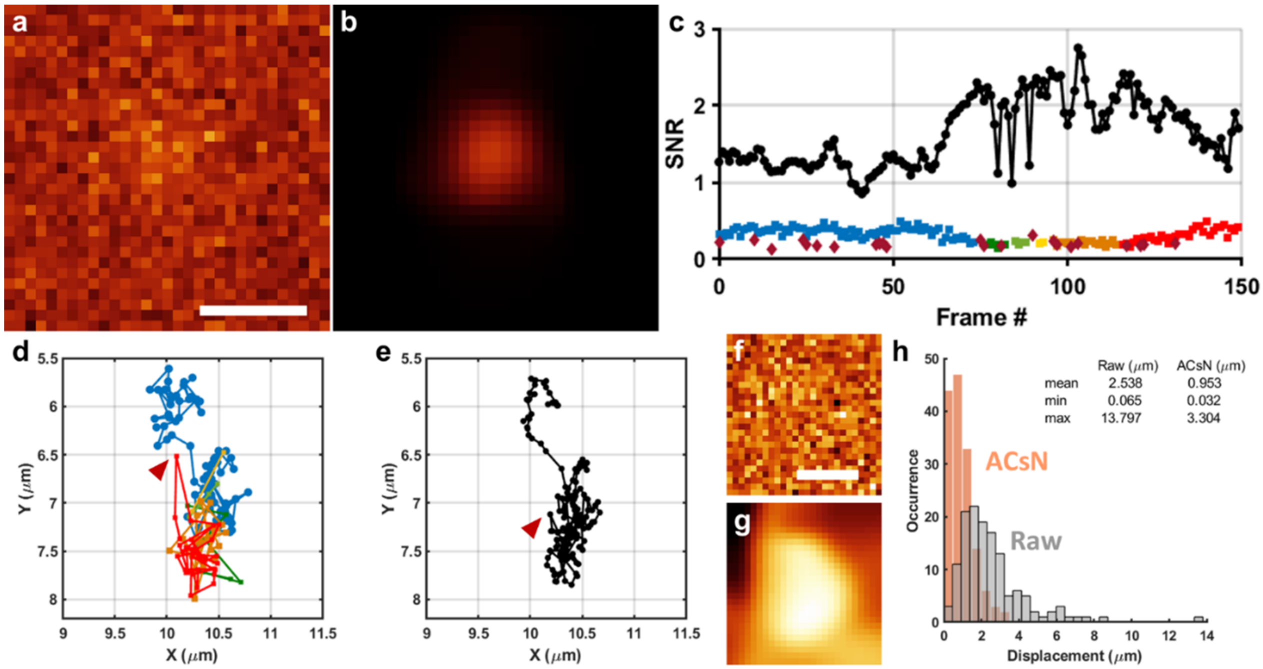

Supplementary Figure 29: ACsN denoising reduces mislocalizations in single-particle tracking. (a) Representative frame of a sequence of 150 frames following the motion of a 1 µm fluorescent bead diffusing in water. Exposure time: 1 ms. (b) The same frame shown in (a) after ACsN denoising. (c) SNR calculated for each localization in each frame. The colors correspond to the corresponding tracks in (d) and (e). The crimson diamonds correspond to mislocalizations in the analysis of the raw data. (d-e) Results of particle tracking before and after denoising, respectively. Different colors represent separate tracks. (f,g) Zoomed-in image of the tracked particle before and after denoising in the frame corresponding to the position marked by the red arrow in (d) and (e), respectively. (h) Histogram and statistical analysis showing the distribution of the displacement evaluated for each couple of linked time points in (d), gray bars, and (e), red bars. Scale bars: 1 µm.

appears more defocused, allowing for a more precise identification of the intensity pattern (see Supplementary Figure 31d-k).

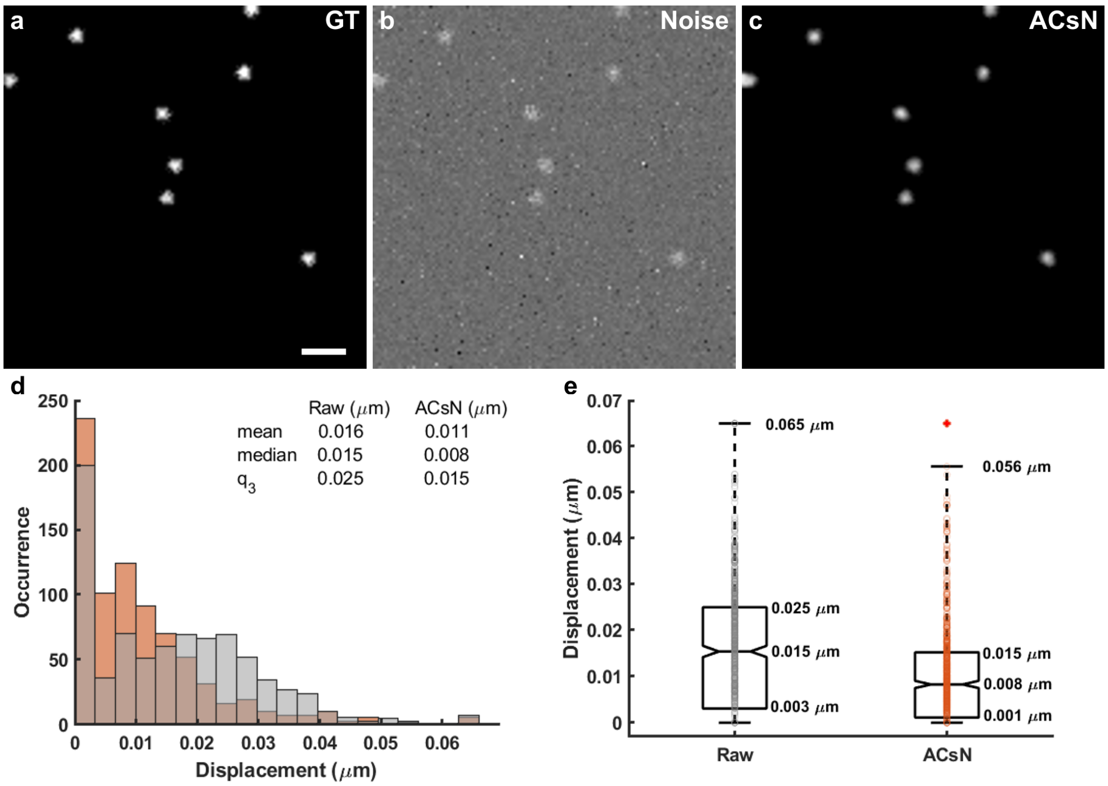

Supplementary Figure 30: ACsN denoising improves the localization precision. (a) Simulated fluorescent beads. The same frame was repeated 100 times and dynamic readout and photon noise were added to each frame to simulate the acquisition of still beads. (b) Representative frame after noise addition. (c) The same frame shown in (b) after ACsN denoising. (d) Histogram and statistical analysis of the displacements evaluated from both the noisy (gray) and denoised (red) data. q_3 indicates the third quartile, within which are contained 75% of the data. (e) Box and whiskers representation of the distributions shown in (d). It is interesting to notice that the notches around the two median values do not overlap, meaning a statistical significance in their difference (>95%). GT: Ground truth. Scale bar: 1 μm.

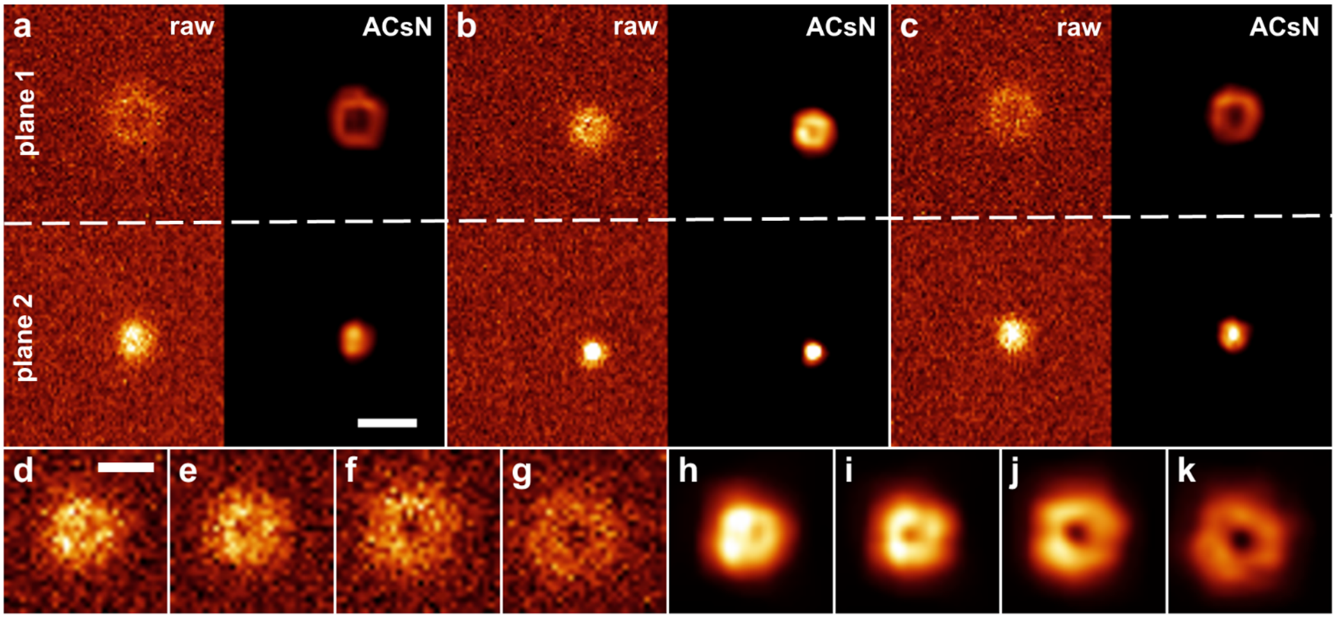

Supplementary Figure 31: Three-dimensional single-particle tracking with biplane microscopy. (a-c) Representative frames of a sequence of 500 images following the diffusion of a 1 µm fluorescent particle in water using biplane microscopy. The frame rate is 1 kHz and the distance between the two focal planes is 500 nm. (d-g) Zoomed-in images on the diffusing particle in the most defocused image (plane 1) in 4 consecutive frames. (h-k) the same particle in (d-g) after ACsN denoising. The insets in (d-k) display the comparision in the intensity profiles. Scale bars: 2 µm (a), 1 µm (d).

Supplementary Note 5. Comparison with the state-of-the-art

The noise in digital images defines the minimum expected variance in the measured intensity values, so that differences in measurements that lie within the noise variance cannot be attributed to the specimen[38]. In addition, biological specimens have a certain level of natural variability, so variance seen in measurements made on different cells results from both the biological variability and measurement errors. For this reason, it is necessary to devise denoising strategies that can reduce the inaccuracies and imprecisions introduced by the noise during acquisition with minimal assumptions about the sample itself.

Comparison with block matching algorithms. Our method is a novel implementation of non-local means for denoising, specifically designed to address sCMOS-related noise in fluorescence microscopy. The novelty of the ACsN algorithm essentially resides in the combination of a system-aware approach with content-adaptive sparse filtering. The former is achieved by camera calibration and OTF estimation, while the latter is obtained by collaborative denoising based on block matching[14,20].

During the last years, the family of block matching algorithms has been extensively adopted in the processing of natural images, but so far it has not obtained a real breakthrough in fluorescence microscopy[39]. In fact, the existing methods work in absence of any prior information concerning the imaging system or the noise statistics (non-Bayesian approaches). This way, they strictly rely on the preliminary grouping step, whose performance is dependent on the SNR. Thereby, a low SNR leads to suboptimal grouping, severely affecting the image quality[40]. This limitation is largely overlooked because of the typical high SNR of the natural scenes, but becomes restrictive for fluorescence microscopy that has an exceedingly limited photon-budget.

On the contrary, our implementation significantly advanced the approach by employing the inherent characteristics of the imaging system and biological samples of fluorescence microscopy. We solve the problem taking advantage of the knowledge of the microscopy system, which is used to obtain a continuously informed analysis of noise statistics, while collaborative filtering takes advantage of the prominent non-local similarity due to the high specificity of fluorescent samples to maximize the efficiency of the algorithm. This results in a thorough, quantitative correction of noise tailored to solve the generic problem in fluorescence microscopy and to enable fast, quantitative biological observation in a wide range of imaging conditions (see Supplementary Note 4).

This specific development allows ACsN to perform substantially better quantitative denoising of microscopy images than other block matching algorithms (see Supplementary Figure 32). In particular, for experimental data we see an improvement of two orders of magnitude in MSE (see Supplementary Figure 33). The input image used in this comparison was acquired with a TIRF microscope and shows a group of microtubules from a HeLa cell. The exposure time was 10 ms. Next, a sequence of 100 frames of the same field of view but with longer exposure time, 111 ms, was taken. This sequence was averaged in order to suppress any residual noise and used as the ground truth in the evaluation of MSE and SSIM. For fair

comparison, we used the state-of-the-art RF3D algorithm[20], because it also takes into account the presence of the fixed-pattern noise.

Comparison with sCMOS-related denoising methods. Recently, Liu et al. proposed a noise correction algorithm for sCMOS camera (NCS) in order to address the same problem and demonstrated its application for wide-field microscopy[41]. NCS comprises two main steps: low-pass filtering of the input image followed by the correction of pixel fluctuation. The latter is performed by the iterative minimization of a cost function evaluated on the estimated variance of the readout noise for every pixel. However, this approach has a few limitations. First, low-pass filtering requires a careful calibration of the OTF of the experimental setup in order to avoid the undesired loss of useful details of the signals, which results in image blurring. Next, camera noise is white noise, so it is present at all frequencies and cannot be effectively removed by low-pass filtering (but just blurred). Thirdly, the second denoising step corrects pixel values to limit signal fluctuation but does not solve the issue of lingering noise, noticeable especially in correspondence of the background (see Supplementary Figure 34 insets).

These limitations result in a loss of image quality and less-than-optimal noise correction. We evaluated this using SSIM and observed that ACsN provides an improvement up to two-fold greater than NCS (see Supplementary Figure 34). Experimental data show also a seven-fold improvement of the mean square error (MSE) (see Supplementary Figure 35a-d) and a careful observation of experimental images shows how the presence of lingering noise in NCS images can hinder the recovery of fine details (see Supplementary Figure 35e-h).

Furthermore, NCS is an iterative algorithm and this impairs its computational speed, which is approximately 100 times slower than the ACsN runtime (see Supplementary Note 6). Finally, NCS has been used for TIRF microscopy using a commonly-used sCMOS camera (Hamamatsu ORCA Flash 4 v.3) but the algorithm has not been demonstrated for other advanced microscopy techniques or different sensors like industrial-grade CMOS, which could be especially interesting because they have stronger readout noise and its correction becomes more relevant.

In summary, we observed that ACsN produces more reliable results than other general-purpose block-matching algorithms and a better suppression of camera-related noise than NCS. In addition, unlike other denoising methods for microscopy implemented for specific cases[41–43], we demonstrated the wide scope of ACsN by showing its application in many different cases, spanning from low-cost fluorescence imaging to more advanced techniques such as super-resolution or lattice light-sheet microscopy. For a discussion about the limitations and notable outlooks of ACsN, please refer to Supplementary Note 8.

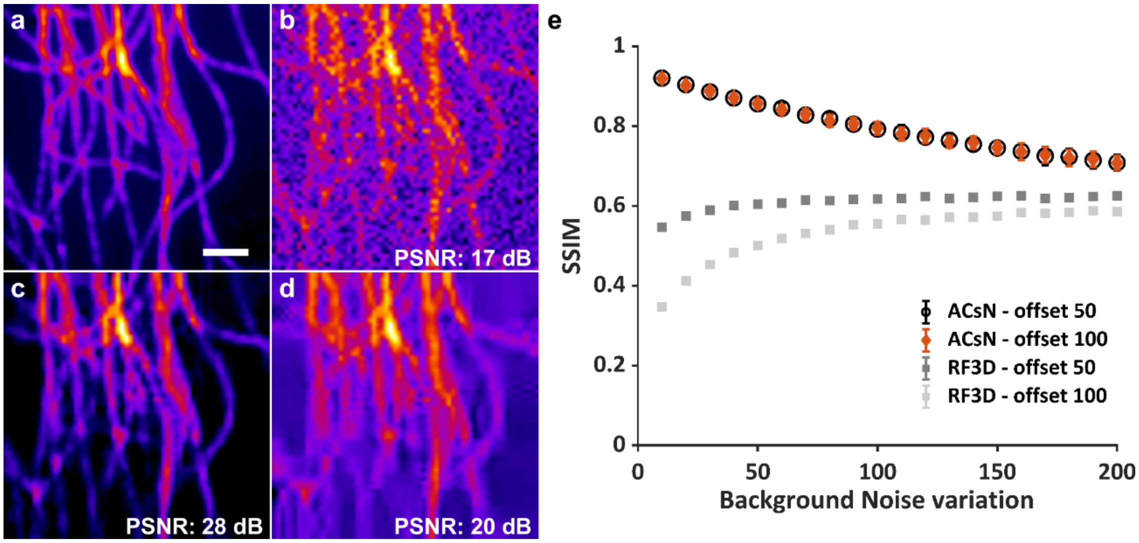

Supplementary Figure 32: Comparison of denoising results for quantitative imaging using ACsN and the state-of-the-art block-matching denoising algorithm. (a) Simulated image of fluorescent microtubules. (b) Simulated noisy image of (a). To simulate the fixed-pattern noise, we scaled pixel values according to a simulated sCMOS gain map and added an intensity offset. We simulated datasets with offsets of 50 and 100 a.u., in particular, to this image was added an offset of 100 a.u. (c) Image (b) processed with ACsN. (d) Image (b) processed with RF3D. Notice the presence of diverse artifacts in both the signal and background areas in (d). (e) Plot of the structural similarity index measurement (SSIM) as a function of the background noise variation. Each point represents the average SSIM evaluated over 50 images and each error bar the corresponding standard deviation. The data relative to the images processed with RF3D are plotted in dark and light gray, corresponding to the intensity offsets of 50 and 100, respectively. In black and red are plotted the data relative to the images processed with ACsN, with offsets of 50 and 100, respectively. Unlike ACsN, the block-matching denoising algorithm shows less satisfactory performance for microscopy images and also a dependence of the efficiency on the offset value. Scale bar: 2 µm.

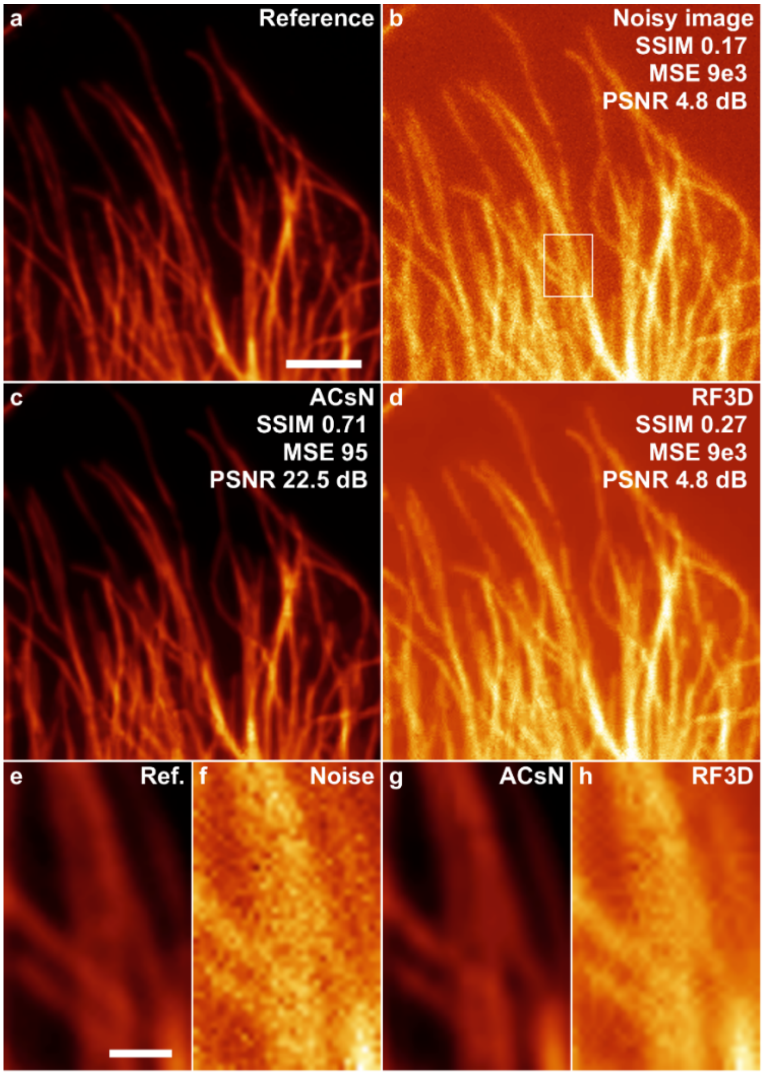

Supplementary Figure 33: Comparison with state-of-the-art block-matching denoising with experimental data. (a) TIRF image of microtubules in fixed HeLa cells. This image was obtained by averaging 100 frames of a fixed HeLa cell acquired with an exposure time of 111 ms. The intensity of (a) has been scaled by 11x for fair comparison with other images. (b) The same sample acquired with an exposure time of 10 ms. (c) Image (b) processed with ACsN. (d) Image (b) processed with RF3D. The values for SSIM, MSE and PSNR in (b, c, d) were evaluated using (a) as the reference image. (e-h) Zoomed-in images of the regions in (a-d) marked by the white box in (b), respectively. The image in (g), restored using ACsN, exhibits evidently closer to (e) compared to the RF3D (h) result, in agreement with the quantitative measurements. Scale bars: 800 nm (a), 160 nm (e).

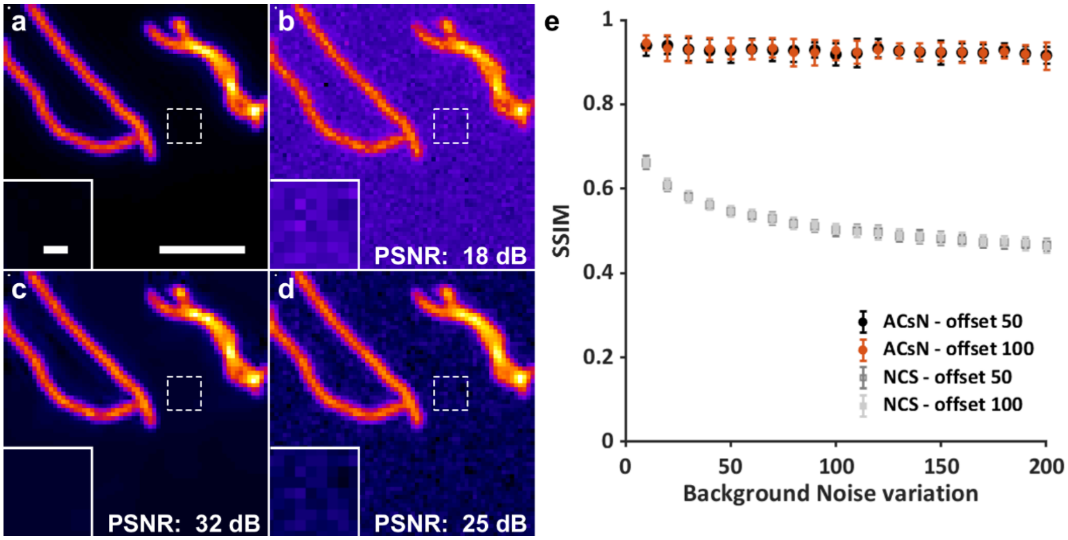

Supplementary Figure 34: Comparison with the state-of-the-art sCMOS denoising. (a) Simulated image of fluorescent microtubule filaments. (b) Simulated noisy image of (a). To simulate the fixed-pattern noise, we scaled pixel values according to a simulated sCMOS gain map and added an intensity offset. We simulated datasets with offsets of 50 and 100 a.u., in particular, to this image was added an offset of 100 a.u. (c) Denoised image using ACsN. (d) Denoised image using NCS. To better compare the residual noise in both restored image, a zoom-in of the same background region (white box) in (a-d) is shown in the corresponding insets. (e) Plot of the structural similarity index (SSIM) as a function of the background noise variation. Each point represents the average SSIM evaluated over 50 images and each error bar the corresponding standard deviation. The data relative to the images processed with NCS are plotted in dark and light gray, with the intensity offsets of 50 and 100, respectively. In black and red are plotted the data relative to the images processed with ACsN, with the offsets of 50 and 100, respectively. Each data point represents the average SSIM calculated over 100 images and the corresponding standard deviation is shown in the error bars. It is shown that an almost two-fold improvement of structural similarity using ACsN in comparison with the state-of-the-art. Scale bar: 2 µm (a), 200 nm (a, inset) .

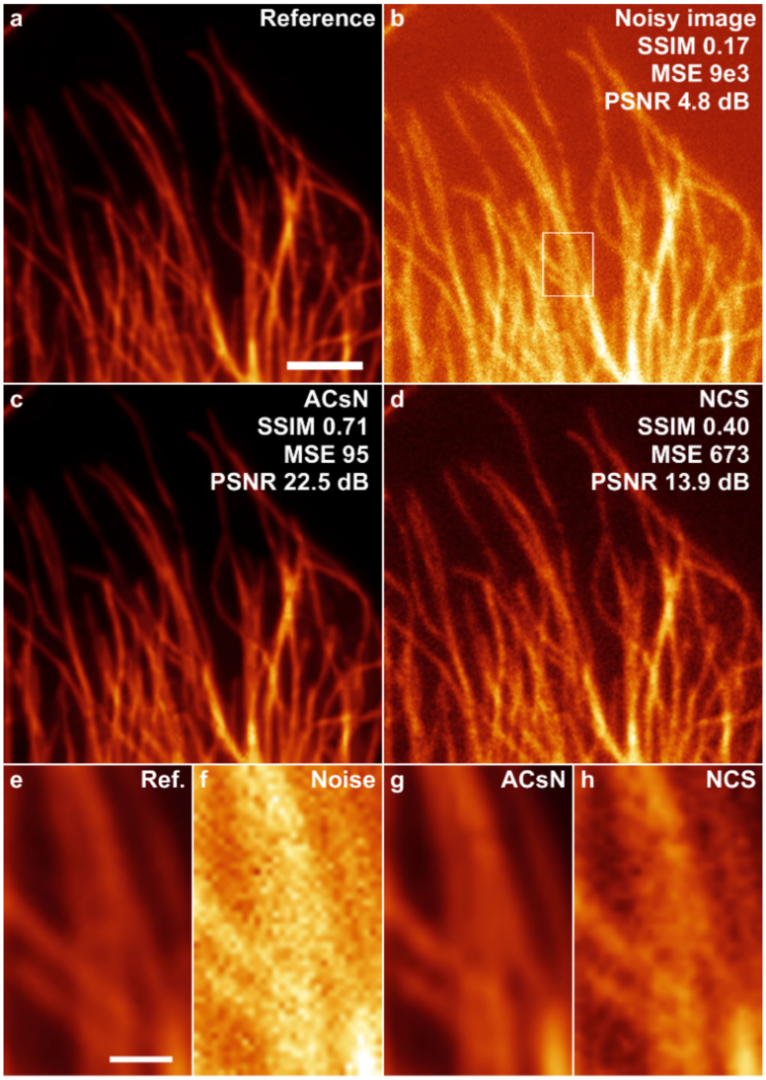

Supplementary Figure 35: Comparison with the state-of-the-art sCMOS denoising using experimental data. (a) TIRF microscopy image of microtubules in fixed HeLa cells. This image was obtained by averaging 100 frames of a fixed Hela cell acquired with an exposure time of 111 ms. The intensity of (a) has been scaled by 11x for fair comparison with other images. (b) The same sample acquired with an exposure time of 10 ms. (c) Image (b) processed with ACsN. (d) Image (b) processed with NCS. The values for SSIM, MSE and PSNR in (b, c, d) were evaluated using (a) as the reference image. (e-h) Zoomed-in images of the regions in (a-d) marked by the white box in (b), respectively. The image in (g), restored using ACsN, exhbits evidently closer to (e) compared to the NCS (h) result, in agreement with the quantitative measurements. Scale bars: 800 nm (a), 160 nm (e).

Supplementary Note 6. Runtime

The runtime of ACsN can vary depending on the image size and on image quality, which may trigger the video processing step. Here we report the performance of our software under various image sizes both with and without video processing on a desktop computer using 6 cores i7-8700, 3.2 GHz CPU (see Supplementary Table 8). Image sequences are divided by ACsN into independent sub-sequences to allow the user to run the software also in the CPU parallel processing mode. The data shown in Supplementary Table 8 have been evaluated averaging the results obtained over 100 images.

Using parallel CPU computing, the processing speed for a 256x256 image is approximately 0.09 s per frame, which is 100 times faster than NCS with default setting and similar computational power (9.03 s with 6 cores at 3.4 GHz using parallel CPU computing)[41]. When the optional video modality is active, ACsN runs an additional processing step to optimize the denoising performance and the runtime goes up to 0.43 s per frame. However, the algorithm can automatically turn the video modality on and off in order to keep the runtime as low as possible while preserving the image quality (see Supplementary Note 2.5). Furthermore, we expect that the runtime can be further improved with parallel GPU programming[17].

Supplementary Table 8: Computational speed per frame of the ACsN algorithm in different conditions.

	**Parallel mode off**		**CPU Parallel mode (6 cores)**	
**Image Size (px)**	Video off	Video on	Video off	Video on
**64x64**	0.04 s	0.09 s	0.01 s	0.04 s
**128x128**	0.10 s	0.29 s	0.03 s	0.13 s
**256x256**	0.33 s	1.12 s	0.09 s	0.43 s

Supplementary Note 7. No-reference assessment of image quality

We have quantitatively assessed the utilization of ACsN, which results in a significant reduction of the errors in intensity measurements given by noisy pixel fluctuations and a high fidelity of image restoration at varying light levels and imaging conditions. We have employed full-reference image quality assessment metrics, such as MSE, PSNR and SSIM, to quantify the performance of ACsN using both experimental and numerical results. These assessments have validated the feasibility of ACsN and provided quantitative information of the denoised image quality. However, in practice, a reference, noise-free dataset is generally not available. This section proposes a strategy that allows for no-reference evaluation of image quality in practice.

To address this problem, blind image quality metrics are emerging[44,45]. Blind image quality assessment has the aim to devise strategies that can predict quality of distorted images with as little prior information about the images or their distortions as possible. In particular, we derived and implemented in our algorithm a metric initially proposed by Kong et al.[46] for no-reference assessment of image quality (Q-score).

This metric is based on the fact that the photon shot noise and readout noise are independent in the original image. It evaluates the image quality using a method noise image (MNI) instead of the reference[15]. The MNI is evaluated as the difference between the input noisy image (I) and the denoised image ($\hat{I}$):

$$M = I - \hat{I} \tag{17}$$

Ideally, the MNI contains only the noise part so that correlation between the noisy image and the MNI, $N = \rho(I, M)$, will be higher in correspondence of the background or regions where the

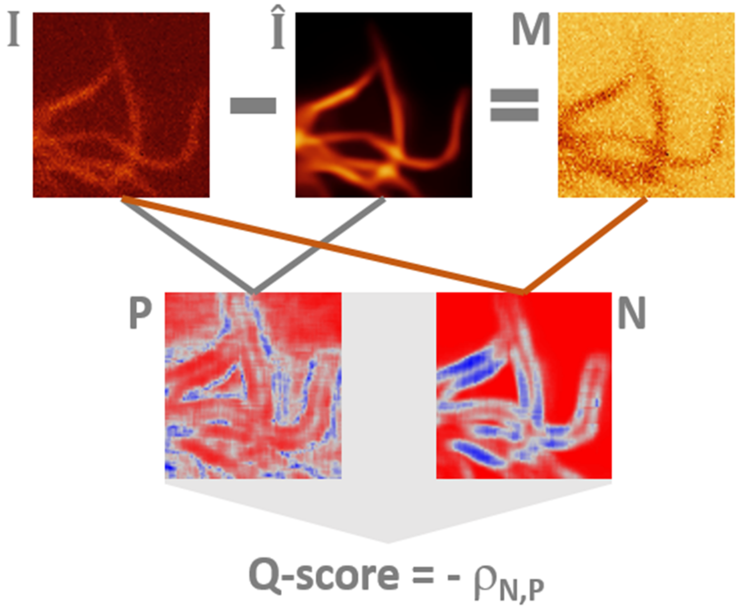

Supplementary Figure 36: Graphical depiction of no-reference assessment of image quality (Q-score). Please notice that the noise variation can be lower across small areas so that I and $\hat{I}$ can be locally correlated and P can assume positive values also in correspondence of some regions of the background.

signal coming from the sample is constant. On the other hand, the correlation between the noisy image and the denoised image, $P = \rho(I, \hat{I})$, will be high mainly in correspondence of highly-structured regions. In both cases, the comparison of the spatial distribution of the image structure is evaluated as:

$$\rho(A, B) = \frac{\sigma_{AB} + c}{\sigma_A \sigma_B + c} \tag{18}$$

where σ_A and σ_B are the standard deviations of images A and B, respectively, σ_{AB} is their cross-correlation, and c is a small constant to ensure stability when the denominator is too small.

In regions that are dominated by structures and have large P values, the term N should be as small as possible, and vice versa. We can measure such interdependent relationship between them using Pearson's linear coefficient and compute the image quality score of the denoised image $\hat{I}$ as the linear correlation coefficient of two structure similarity maps N and P:

$$\text{Q-score} = -\rho(N, P) \tag{19}$$

where the minus indicates that the quality is higher when there is negative correlation between N and P (see Supplementary Figure 36).

The experimental data have validated the consistency to reveal denoised image quality between the no-reference Q-score and the full-reference scores SSIM and PSNR (for example, see Supplementary Figure 37). This strategy provides a quantitative module for users' assessment of image restoration quality for fluorescence microscopy, where the reference image is not available in many practical cases.

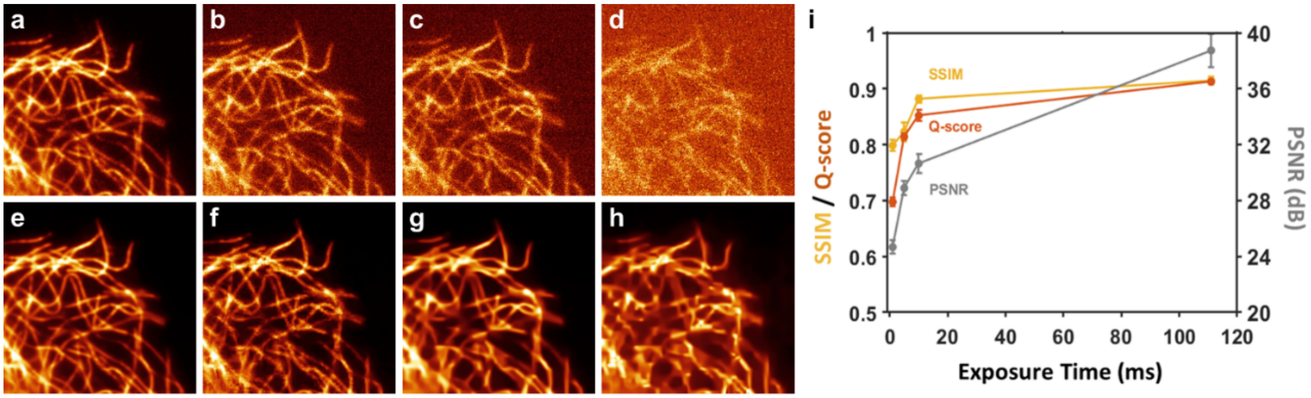

Supplementary Figure 37: Comparison of the no-reference Q-score with SSIM and PSNR. (a-d) Representative frames of 4 experimental sequences of fixed microtubules aquired with different exposure times: 111 ms (a), 10 ms (b), 5 ms (c), and 1 ms (d). (e-h) ACsN denoised images of the frames shown in (a-d), respectively. (i) Plot of SSIM, PSNR and Q-score evaluated for the images in (e-h). Each value and its corresponding error were calculated as the average and the standard deviation over 100 frames, respectively. The results of no-reference Q-score show a consistent trend as those of SSIM and PSNR using the average of 100 frames in (a) as the reference image.

Supplementary Note 8. Discussion & Outlook

We have introduced ACsN, an algorithm designed for the automatic correction of sCMOS-related noise for fluorescence microscopy. The key feature of this approach is the combined usage of camera calibration with layered sparse filtering. This results in content-adaptive image restoration aided by system parameters.

Other existing methods based on collaborative filtering require a prior knowledge of noise statistics or try to evaluate it from the input images[14,39,40,42,43,47]. Instead, ACsN targets specifically camera-related noise and thus performs such estimates taking advantage of the knowledge of the imaging system and a one-time calibration of the camera sensor. This yields a more robust and efficient performance while the layered filtering approach maximizes the correction capacity for input sequences with low SNR.

Such system-aware strategy has also been adopted by approaches based on low-pass filtering[41,48]. However, low-pass filtering by definition provides an inadequate correction of the camera noise. In fact, as white noise is present at all frequencies, it cannot be completely removed by the choice of a cutoff frequency, inevitably implying a tradeoff between noise correction and detail preservation.

Recently, learning-based image denoising has been demonstrated with wide-field[49], two-photon[50], and confocal fluorescence microscopy[29] with excellent results. Learning based algorithms provide exceptional results and maximize the information recovered, given a comprehensive training database with respect to the input image. However, its application is limited by two main factors: the loss of quantitative information of the signal due to the nonlinearity of the neural network predictions[29], and the dependency on adequate training data, which is potentially challenging for discovery of information beyond the training[30]. In contrast, ACsN adapts to the content of each image by evaluating its self-similarity and sparsity. It stands, thus, somewhat in the middle between content-aware learning-based networks and other content-agnostic methods. This makes ACsN feasible for any images, free of existing libraries or prior assumptions, while retaining true signal values, thus maintaining all quantitative information for further analysis.

We have shown how ACsN performs in diverse experimental conditions, with different sensors and for a wide range of applications, from super-resolution to low-cost and miniaturized systems (see Supplementary Table 4). We have also demonstrated that the restoration of images with moderate levels of noise can result in a major improvement of the downstream analysis of, for example, deconvolution microscopy or STORM. In the latter case, although specific algorithms for STORM reconstruction of sCMOS-based images have been proposed[48,51], ACsN gives users the advantage to address the challenge of denoising and still choose the SMLM software that better fits their experimental conditions[52]. Processing time series of both fixed and live samples, we have observed a substantial reduction of pixel fluctuations and thus of the measurement errors. Users should be aware that such errors may not be totally removed due to the photon shot noise, but we observed that they become comparable to the error level of an ideal camera (see 3.3 Reduction of Pixel Fluctuation). This

allows an acceptable denoising accuracy even at low light intensity, down to 5-10 photons per pixel (see 3.4 Dependency on the incoming photon flux). Below such imaging conditions, the efficiency of ACsN image restoration is reduced and its performance starts to decrease. This is due to both the very low SNR and the validity of our noise model. In fact, we estimate the noise variation σ_N assuming that photon noise can be considered locally Gaussian-distributed, which can be safely assumed when the photon flux is greater than 5 photons per pixel.

Importantly, the threshold for acceptable restoration accuracy can vary depending on the specific camera, specimen or imaging technique used. For this reason, we recommend to calibrate and test the algorithm before applying it to any new type of data. In particular, users should exercise a special care in their camera calibrations, because errors in this phase will propagate to the whole denoising process. To help users to avoid such circumstances, an evaluation of the restoration quality was added to the algorithm in order to identify suspicious images where denoising may not be accurate.

In some applications, the SNR of the input sequence is improved by frame averaging during acquisition or in post processing[38]. Interestingly, it has been shown that such improvement can be successfully combined with collaborative sparse filtering[40]. In agreement with such results, simulations have shown that the improvement of ACsN restoration after frame averaging of the input image is higher than an alternative algorithm based on low-pass filtering (see Supplementary Figure 38). In this case, it showed an average improvement of 10.6 dB in PSNR by averaging the input image over 50 frames before ACsN denoising. Instead, the gain for low-pass filtering is lower because of the residual blurred noise. As an outlook, it would be interesting to explore this feature with techniques like multi-photon microscopy. Indeed, in this case, photodamage responds nonlinearly to the increase in excitation power at higher order rates compared to fluorescence emission[53,54]. So, to improve the SNR of multi-photon fluorescence it is less harmful to image the sample using frame averaging of repeated scans than increasing the illumination power.

The ultimate limitation for the application of any image restoration approach in fluorescence microscopy is the intrinsic difficulty to assess precision and accuracy for each restored image[29]. In fact, often only one measurement can be taken or sample motion limits the usage of data for error analysis and, unlike cases like image deconvolution or super-resolution[52], the input image cannot be used as reference image (the PSF is a constant of the system while noise varies in each image). To address this issue, we suggest the users to adopt the same guidelines for the validation of an imaging system reported by Jost & Waters 2019[55]. For example, using fixed samples it is possible to estimate both the precision (see 3.3 Reduction of Pixel Fluctuation) and the accuracy (see 3.4 Dependency on the incoming photon flux) expected for a certain kind of measurement. However, a general method to assess the presence of restoration artifacts is still lacking. For this reason, we proposed the adoption of a no-reference metric based on the evaluation of the MNI. We have demonstrated that this can be a reliable metric and we anticipate that its reliability would be furtherly improved by the development of an application-driven variant capable to spot feature artifacts or distortions introduced by image processing. In this sense, several methods have recently been developed based on image

models[44] or deep learning[45] but, to the best of our knowledge, none has been implemented for fluorescence microscopy.

Furthermore, it should be noted that we have observed that ACsN works less efficiently in conditions of strong background fluorescence. In fact, the presence of fluorescence due to out-of-focus emitters or improper labeling is not included in the current noise model and is thus not corrected by the algorithm. On the contrary, it is treated as the signal and, depending on the SNR, can lead to an over-smoothing of the features of the sample. However, this is a common problem in fluorescence microscopy and it is good practice to avoid it as much as possible[38]. For this reason, several strategies have been devised to reduce the background fluorescence during the experimental acquisitions or in post processing[38,56]. As an outlook, such a module can be implemented into ACsN for broader applications.

Likewise, highly non-uniform illumination can be problematic during the processing of images with large field of view. This may affect the performance of the sparse filtering, which assumes the noise to be spatially-invariant. However, this is considerably mitigated by the use of patch-based processing in this work. Indeed, the enhanced intensity uniformity within individual patch groups, compared to the whole image, provides a great stability to this approach. In fact, the robustness of the block-matching denoising against spatially-varying noise has recently been shown with images corrupted by mixed Poisson-Gaussian noise[39]. Furthermore, ACsN can provide a different local estimate of the noise variation for different subsets of the image, thus limiting the effect of non-uniform illumination. Nonetheless, we regard as an outlook develop an adaptive filtering strategy that could further improve the correction of highly spatially-varying noise, e.g. dark shot noise. We consider that valid

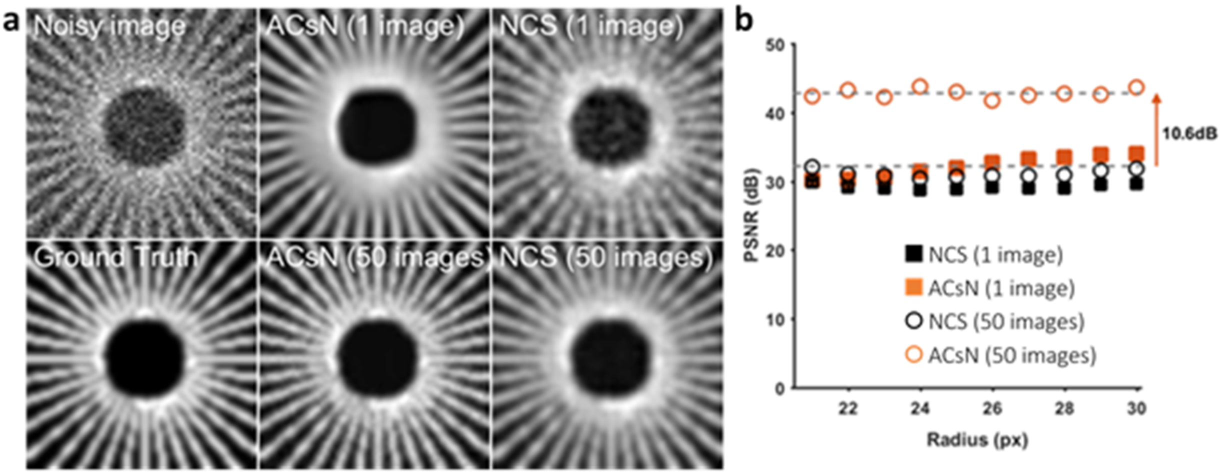

Supplementary Figure 38: Effect of frame averaging over image restoration. The simulated image of a Siemens star was used to quantify the effect of frame averaging at different spatial frequencies. (a) Illustrative frames of the simulated noisy sequence and the denoised images obtained with and without frame averaging with both ACsN and NCS algorithms. (b) Plot of the improvement in PSNR obtained by the two algorithms before and after frame-averaging as a function of the distance from the center of the image. An average improvement of 10.6 dB in PSNR is shown for ACsN after an average over 50 frames. The gain for NCS is lower because of the residual blurred noise due to low-pass filtering.

approaches would be either applying a variance stabilizing transformation in order to standardize the image noise[57], or using a modified Wiener filter based non-local weighted estimation of noise parameters[58]. Alternatively, the minimization with Lagrangian multipliers of an approximate Poisson likelihood cost function would consent us to iteratively refine the image restoration in cases where the noise is highly spatially-variant without having to change the core algorithm. These strategies would not only improve the performance of ACsN, but also extend its use to other camera types beyond CMOS, such as CCD and EM-CCD cameras.

Besides, the evaluation of image quality after 2D filtering is intensity-dependent, so the difference of intensity between the center and the periphery of the image generated by a non-uniform illumination could prevent the 3D video filtering to be activated in the automatic mode, which could produce suboptimal results. In specific cases, this can be spotted by examining the Q-score and corrected manually. For big datasets, instead, this could be solved by introducing flat-field correction hardware or monitoring algorithms in the processing pipeline[55,56].

Finally, we have shown the application to 3D and 4D data stacks of different color and spatio-temporal combinations. However, ACsN currently supports only 3D data stacks so that, in some cases such as processing lattice light sheet imaging data, we utilized either the time self-similarity or the volumetric self-similarity. This strategy may not maximize the information extraction present in volumetric time-lapses. We anticipate that the implementation of features to handle multidimensional data would be a valuable addition to the current software. In particular, the implementation of a search window in the 4D space would further improve the image restoration of volumetric time-lapse sequences.

As another outlook, we plan to investigate the performance of ACsN with industrial-grade CMOS cameras in order to optimize the image restoration and improve the quality of low-cost sensors. We anticipate that this would draw interests of the scientific community in an effort to approach the performance of sCMOS cameras at an affordable price. It would also spur the adoption of ACsN to maximize the performance in various miniaturized or lab-on-a-chip systems.

Other future developments will include the release of a Python version of the software for wider usability, and the implementation of parallel GPU computing for even faster runtimes, which would be another helpful addition that could furtherly enhance the performance and range of applications for ACsN.

Supplementary References

1. Babcock, H. P. Multiplane and spectrally-resolved single molecule localization microscopy with industrial grade CMOS cameras. *Sci. Rep.* **8**, 4–11 (2018).
2. Flusberg, B. A. *et al.* High-speed, miniaturized fluorescence microscopy in freely moving mice. *Nat. Methods* **5**, 935–938 (2008).
3. Theuwissen, A. J. Solid-state imaging with charge-coupled devices. *Solid-State Sci. Technol. Libr. (SSST), Dordrecht, Bost. Kluwer Acad. Publ. |c1995* (1995).
4. Brouk, I., Nemirovsky, A., Alameh, K. & Nemirovsky, Y. Analysis of noise in CMOS image sensor based on a unified time-dependent approach. *Solid. State. Electron.* **54**, 28–36 (2010).
5. Irie, K., McKinnon, A. E., Unsworth, K. & Woodhead, I. M. A model for measurement of noise in CCD digital-video cameras. *Meas. Sci. Technol.* **19**, (2008).
6. Moomaw, B. *Camera technologies for low light imaging: Overview and relative advantages. Methods in Cell Biology* **114**, (Elsevier Inc., 2013).
7. Foi, A., Trimeche, M., Katkovnik, V. & Egiazarian, K. Practical Poissonian-Gaussian noise modeling and fitting for single-image raw-data. *IEEE Trans. Image Process.* 1–18 (2007).
8. Aguerrebere, C., Delon, J., Gousseau, Y. & Musé, P. Study of the digital camera acquisition process and statistical modeling of the sensor raw data. *Prepr. HAL* 1–7 (2012).
9. Healey, G. E. & Kondepudy, R. Radiometric CCD Camera Calibration and Noise Estimation. *IEEE Trans. Pattern Anal. Mach. Intell.* **16**, 267–276 (1994).
10. Janesick, J. R. Photon Transfer Noise Sources. in *Photon Transfer* 21–34 (2009). doi:10.1117/3.725073.ch3
11. De La Rosa Vargas, J. I., Villa, J. J., Gonzalez, E. & Cortez, J. A tour of nonlocal means techniques for image filtering. *2016 Int. Conf. Electron. Commun. Comput. CONIELECOMP 2016* 32–39 (2016). doi:10.1109/CONIELECOMP.2016.7438548
12. Goodman, J. W. *Introduction to Fourier optics*. (Roberts & Co, 2005).
13. Hanser, B. M., Gustafsson, M. G. L., Agard, D. A. & Sedat, J. W. Phase-retrieved pupil functions in wide-field fluorescence microscopy. *J. Microsc.* **216**, 32–48 (2004).
14. Dabov, K., Foi, A., Katkovnik, V. & Egiazarian, K. Image denoising with block-matching and 3D filtering. in *Proc. of SPIE-IS&T Electronic Imaging* **6064**, 606414 (2006).
15. Buades, A., Coll, B. & Morel, J. M. Image denoising by non-local averaging. *ICASSP, IEEE Int. Conf. Acoust. Speech Signal Process. - Proc.* **II**, ii-25 (2005).
16. Kervrann, C. & Boulanger, J. Optimal spatial adaptation for patch-based image denoising. *IEEE Trans. Image Process.* **15**, 2866–2878 (2006).
17. Honzátko, D. & Kruliš, M. Accelerating block-matching and 3D filtering method for image denoising on GPUs. *J. Real-Time Image Process.* 1–15 (2017). doi:10.1007/s11554-017-0737-9
18. Lebrun, M. An Analysis and Implementation of the BM3D Image Denoising Method. *Image Process. Line* **2**, 175–213 (2012).
19. Maggioni, M., Boracchi, G., Foi, A. & Egiazarian, K. Video denoising, deblocking, and

enhancement through separable 4-D nonlocal spatiotemporal transforms. *IEEE Trans. Image Process.* **21**, 3952–3966 (2012).

20. Maggioni, M., Sánchez-Monge, E. & Foi, A. Joint removal of random and fixed-pattern noise through spatiotemporal video filtering. *IEEE Trans. Image Process.* **23**, 4282–4296 (2014).
21. Shan Zhu & Kai-Kuang Ma. A new diamond search algorithm for fast block-matching motion estimation. *IEEE Trans. Image Process.* **9**, 287–290 (2000).
22. Gonzalez, R. C., Woods, R. E. & Eddins, S. L. *Digital Image Processing Using MATLAB*. (Gatesmark Publishing, 2003).
23. Heintzmann, R. Band Limit and Appropriate Sampling in Microscopy. *Cell Biol. Four-Volume Set* **3**, 29–36 (2006).
24. Sarder, P. & Nehorai, A. Deconvolution methods for 3-D fluorescence microscopy images. *IEEE Signal Process. Mag.* **23**, 32–45 (2006).
25. Richardson, W. H. Bayesian-Based Iterative Method of Image Restoration. *J. Opt. Soc. Am.* **62**, 55 (1972).
26. Lucy, L. B. An iterative technique for the rectification of observed distributions. *Astron. J.* **79**, 745 (1974).
27. Culley, S. *et al.* Quantitative mapping and minimization of super-resolution optical imaging artifacts. *Nat. Methods* **15**, 263–266 (2018).
28. Gustafsson, N. *et al.* Fast live-cell conventional fluorophore nanoscopy with ImageJ through super-resolution radial fluctuations. *Nat. Commun.* **7**, 1–9 (2016).
29. Weigert, M. *et al.* Content-aware image restoration: pushing the limits of fluorescence microscopy. *Nat. Methods* **15**, 1090–1097 (2018).
30. Belthangady, C. & Royer, L. A. Applications, promises, and pitfalls of deep learning for fluorescence image reconstruction. *Nat. Methods* (2019). doi:10.1038/s41592-019-0458-z
31. Kusumi, A., Tsunoyama, T. A., Hirosawa, K. M., Kasai, R. S. & Fujiwara, T. K. Tracking single molecules at work in living cells. *Nat. Chem. Biol.* **10**, 524–532 (2014).
32. Manzo, C. & Garcia-Parajo, M. F. A review of progress in single particle tracking: from methods to biophysical insights. *Rep Prog Phys* **78**, 124601 (2015).
33. Tinevez, J. Y. *et al.* TrackMate: An open and extensible platform for single-particle tracking. *Methods* **115**, 80–90 (2017).
34. Chenouard, N. *et al.* Objective comparison of particle tracking methods. *Nat. Methods* **11**, 281–9 (2014).
35. Juette, M. F. & Bewersdorf, J. Three-dimensional tracking of single fluorescent particles with submillisecond temporal resolution. *Nano Lett.* **10**, 4657–4663 (2010).
36. Juette, M. F. *et al.* Three-dimensional sub-100 nm resolution fluorescence microscopy of thick samples. *Nat. Methods* **5**, 527–529 (2008).
37. Ober, R. J., Ram, S. & Ward, E. S. Localization accuracy in single-molecule microscopy. *Biophys. J.* **86**, 1185–1200 (2004).
38. Waters, J. C. Accuracy and precision in quantitative fluorescence microscopy. *J. Cell Biol.* **185**, 1135–1148 (2009).

39. Meiniel, W., Olivo-Marin, J. C. & Angelini, E. D. Denoising of microscopy images: A review of the state-of-the-art, and a new sparsity-based method. *IEEE Trans. Image Process.* **27**, 3842–3856 (2018).
40. Bianco, V. *et al.* Quasi noise-free digital holography. *Light Sci. Appl.* **5**, e16142 (2016).
41. Liu, S. *et al.* sCMOS noise-correction algorithm for microscopy images. *Nat. Methods* **14**, 760–761 (2017).
42. Coupé, P., Munz, M., Manjón, J. V., Ruthazer, E. S. & Louis Collins, D. A CANDLE for a deeper in vivo insight. *Med. Image Anal.* **16**, 849–864 (2012).
43. Yang, L. *et al.* An adaptive non-local means filter for denoising live-cell images and improving particle detection. *J. Struct. Biol.* **172**, 233–243 (2010).
44. Mittal, A., Soundararajan, R. & Bovik, A. C. Making a 'Completely Blind' Image Quality Analyzer. *IEEE Signal Process. Lett.* **20**, 209–212 (2013).
45. Xie, F., Lu, Y., Bovik, A. C., Jiang, Z. & Meng, R. Application-Driven No-Reference Quality Assessment for Dermoscopy Images With Multiple Distortions. *IEEE Trans. Biomed. Eng.* **63**, 1248–1256 (2016).
46. Kong, X., Li, K., Yang, Q., Wenyin, L. & Yang, M. H. A new image quality metric for image auto-denoising. *Proc. IEEE Int. Conf. Comput. Vis.* 2888–2895 (2013). doi:10.1109/ICCV.2013.359
47. Carlton, P. M. *et al.* Fast live simultaneous multiwavelength four-dimensional optical microscopy. *Proc. Natl. Acad. Sci.* **107**, 16016–16022 (2010).
48. Huang, F. *et al.* Video-rate nanoscopy using sCMOS camera-specific single-molecule localization algorithms. *Nat. Methods* **10**, 653–658 (2013).
49. Krull, A., Buchholz, T.-O. & Jug, F. Noise2Void - Learning Denoising from Single Noisy Images. 2129–2137 (2018).
50. Zhang, Y. *et al.* A Poisson-Gaussian Denoising Dataset with Real Fluorescence Microscopy Images. (2018).
51. Zhang, Z. *et al.* Localization-based super-resolution microscopy with an sCMOS camera. *Opt. Express* **19**, 19156 (2011).
52. Sage, D. *et al.* Quantitative evaluation of software packages for single-molecule localization microscopy. *Nat. Methods* **12**, 1–12 (2015).
53. Patterson, G. H. & Piston, D. W. Photobleaching in two-photon excitation microscopy. *Biophys. J.* **78**, 2159–2162 (2000).
54. Pinkard, H., Corbin, K. & Krummel, M. F. Spatiotemporal rank filtering improves image quality compared to frame averaging in 2-photon laser scanning microscopy. *PLoS One* **11**, 1–13 (2016).
55. Jost, A. P. & Waters, J. C. Designing a rigorous microscopy experiment: Validating methods and avoiding bias. *J. Cell Biol.* **218**, 1452–1466 (2019).
56. Wolf, D. E., Samarasekera, C. & Swedlow, J. R. *Quantitative Analysis of Digital Microscope Images. Methods in Cell Biology* **114**, (Elsevier Inc., 2013).
57. Azzari, L. & Foi, A. Variance Stabilization for Noisy+Estimate Combination in Iterative Poisson Denoising. *IEEE Signal Process. Lett.* **23**, 1086–1090 (2016).

58. Bindilatti, A. A., Vieira, M. A. C. & Mascarenhas, N. D. A. Poisson Wiener filtering with non-local weighted parameter estimation using stochastic distances. *Signal Processing* **144**, 68–76 (2018).
