# Supplementary material for: Fast and accurate sCMOS noise correction for fluorescence microscopy
Source: Nat Commun. 2020 Jan 3;11:94. doi: 10.1038/s41467-019-13841-8 (PMC6941997; doi:10.1038/s41467-019-13841-8)
Supplement: Supplementary file 2 — Description of Additional Supplementary Files [file 41467_2019_13841_MOESM2_ESM.pdf]

Description of Additional Supplementary Files

File name:	Description:
**Supplementary Movie 1**	**Imaging of mitochondria in live human embryonic kidney (HEK) cells.** Video sequence before (left) and after (right) ACsN denoising of mitochondria moving in live HEK cells. The exposure time is 20 ms. Scale bar: 5 μm.
**Supplementary Movie 2**	**Imaging of mitochondria in live human embryonic kidney (HEK) cells.** Video sequence before (left) and after (right) ACsN denoising of mitochondria moving in live HEK cells. The exposure time is 20 ms. Scale bar: 5 μm.
**Supplementary Movie 3**	**Single-particle tracking of a 1-μm fluorescent bead diffusing in water.** Video sequence before (left) and after (right) ACsN denoising. The positions of the localized particles are indicated by purple circles. The estimated trajectories are overlapped to the video. Different colors represent different trajectories. The exposure time is 1 ms. Scale bar: 3 μm.
**Supplementary Movie 4**	**Single-particle tracking with biplane microscopy.** Sequence of 500 frames recording the diffusion of a 1-μm fluorescent particle in water using biplane microscopy. The frame rate is 1 kHz and the exposure time is 1 ms. The distance between the two focal planes is 500 nm. Scale bar: 2 μm.
**Supplementary Movie 5**	**Three-dimensional rendering of a brine shrimp's tail acquired with light-sheet microscopy.** 3D rendering of the volumetric scan of a brine shrimp's tail acquired with SPIM. Each frame of the video corresponds to a rotation of 12 degrees.
**Supplementary Movie 6**	**Maximum intensity projection video of live human lung cancer cells acquired with lattice light-sheet microscopy.** Time-lapse sequence of volumetric scans of live NCI-H1299 NSCLC cells before (left) and after (right) ACsN denoising.
**Supplementary Movie 7**	**Three-dimensional rendering of live human lung cancer cells observed with lattice light-sheet microscopy.** Time-lapse sequence of volumetric scans of live NCI-H1299 NSCLC cells. Each volumetric scan was constituted by 91 slices, with a 104 × 104 × 561 nm voxel, and an interval between scans of 18.444 s. The 3D rendering was created using Imaris 9.3 (Oxford Instruments).
**Supplementary Movie 8**	**Three-dimensional rendering of a live mouse embryonic fibroblast (MEF) observed with lattice light-sheet microscopy.** 3D rendering of a time-lapse sequence of volumetric scans of a live MEF before and after ACsN denoising. Each volumetric scan was constituted by 101 slices, the voxel was 104 × 104 × 200 nm, and the interval between scans of 1.806 s. The 3D rendering was created using Imaris 9.3 (Oxford Instruments).
**Supplementary Software**	ACsN software package and unprocessed example data.
