# Supplementary figures and images for: Fast and accurate sCMOS noise correction for fluorescence microscopy
Source: Nat Commun. 2020 Jan 3;11:94. doi: 10.1038/s41467-019-13841-8 (PMC6941997; doi:10.1038/s41467-019-13841-8)

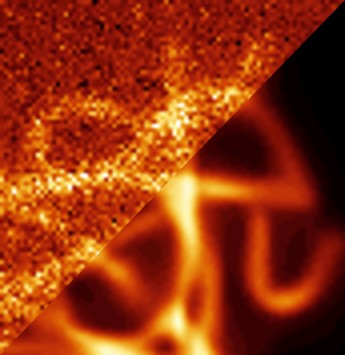

Supplement: Supplementary file 11 — Supplementary Software [file 41467_2019_13841_MOESM11_ESM.zip › ACsN/Picture2.jpg]

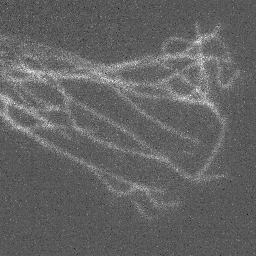

Supplement: Supplementary file 11 — Supplementary Software [file 41467_2019_13841_MOESM11_ESM.zip › ACsN/Test Images/TIRF_01_05ms.tif]

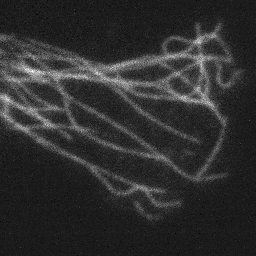

Supplement: Supplementary file 11 — Supplementary Software [file 41467_2019_13841_MOESM11_ESM.zip › ACsN/Test Images/TIRF_01_10ms.tif]

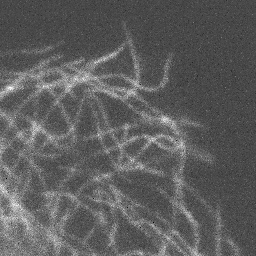

Supplement: Supplementary file 11 — Supplementary Software [file 41467_2019_13841_MOESM11_ESM.zip › ACsN/Test Images/TIRF_02_05ms.tif]

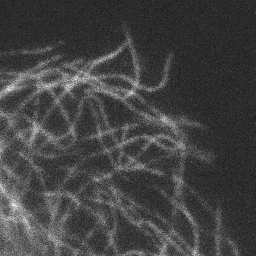

Supplement: Supplementary file 11 — Supplementary Software [file 41467_2019_13841_MOESM11_ESM.zip › ACsN/Test Images/TIRF_02_10ms.tif]
